# High-dose, short-course primaquine after point-of-care G6PD testing for the radical cure of *Plasmodium vivax* malaria: a safety study in Papua New Guinea and Indonesia

**DOI:** 10.1016/j.lanwpc.2026.101903

**Published:** 2026-06-11

**Authors:** Liony Fransisca, Maria Ome-Kaius, Moses Laman, Jeanne Rini Poespoprodjo, Ayodhia P. Pasaribu, Inge Sutanto, Mary Malai, Erni Nelwan, Jacklyn Adella, Cynthia Abegini, Framita Ainur, Yolyne Amdara, Sherley Angeline, Katelyn Brown, Faustina H. Burdam, Ella Curry, Martin Daimen, Paul Daly, Thy Do, Annie Dori, Nicholas M. Douglas, Heike Huegel, Azkarunia P. Hutagalung, Elodie Jambert, Vincent Jimanto, Vali Karo, Enny Kenangalem, Trevor Kelebi, Jacob Kisomb, Ida Laksono, Grant Lee, Benedikt Ley, Kylie Mannion, Julius Plinduo, Irene Pukai, Adela Putri, Megha Rajasekhar, Sharol Ronkentou, Evelien Rosens, Vanessa S. Sakalidis, Ari Satyagraha, Julie A. Simpson, Minerva Theodora, Reynold Ubra, Norman Vakore, Tanyaporn Wansom, Stephan Duparc, Leanne J. Robinson, Ric N. Price

**Affiliations:** a Yayasan Pengembangan Kesehatan dan Masyarakat Papua, Timika, Papua, Indonesia; b Global and Tropical Health Division, Menzies School of Health Research, Charles Darwin University, Darwin, Northern Territory, Australia; c Papua New Guinea Institute of Medical Research, Port Moresby, Papua New Guinea; d Centre for Child Health, Faculty of Medicine, Public Health and Nursing, Universitas Gadjah Mada, Yogyakarta, Indonesia; e Department of Pediatrics, Faculty of Medicine, Universitas Sumatera Utara, Medan, Indonesia; f Tridarma Healthcare Empowerment Foundation, Medan, Sumatera Utara, Indonesia; g Department of Parasitology, Faculty of Medicine, University of Indonesia, Jakarta, Indonesia; h Hanura Primary Health Centre (Puskesmas), Lampung, Indonesia; i Madang Provincial Health Authority, Papua New Guinea; j Burnet Institute, Melbourne, Victoria, Australia; k Medicines for Malaria Venture, Geneva, Switzerland; l Department of Infectious Diseases, Christchurch Hospital, Te Whatu Ora Waitaha, Christchurch, New Zealand; m Department of Medicine, University of Otago, Christchurch, New Zealand; n National Malaria Control Program, Papua New Guinea National Department of Health, Papua New Guinea; o National Research and Innovation Agency (BRIN), Jakarta, Indonesia; p Centre for Epidemiology and Biostatistics, Melbourne School of Population and Global Health, The University of Melbourne, Melbourne, Australia; q Indonesian National Malaria Control Program, Jakarta, Indonesia; r Mimika Health Office, Timika, Papua, Indonesia; s East New Britain Provincial Health Authority, Papua New Guinea; t Centre for Tropical Medicine and Global Health, Nuffield Department of Clinical Medicine, University of Oxford, Oxford, United Kingdom; u Chayun Consulting, Bangkok, Thailand; v Nossal Institute for Global Health, Melbourne School of Population and Global Health, The University of Melbourne, Melbourne, Australia; w Mahidol-Oxford Tropical Medicine Research Unit, Faculty of Tropical Medicine, Mahidol University, Bangkok, Thailand

**Keywords:** *Plasmodium vivax*, Radical cure, Primaquine, G6PD testing, Pharmacovigilance

## Abstract

**Background:**

High total dose primaquine (PQ 7 mg/kg) over 7 days can improve treatment adherence and reduce *Plasmodium vivax* malaria recurrences. We evaluated the safety of pre-treatment glucose-6-phosphate dehydrogenase (G6PD) testing followed by high-dose primaquine for *P. vivax* malaria in Indonesia and Papua New Guinea.

**Methods:**

Patients with *P. vivax* malaria presenting to four community health clinics were screened for G6PD deficiency using the STANDARD G6PD (SD Biosensor, Republic of Korea) and treated with PQ7 (1 mg/kg/day, 7 days) if G6PD normal, PQ14 (0.5 mg/kg/day, 14 days) if G6PD intermediate, and PQ8W (0.75 mg/kg/week, 8 weeks) if G6PD deficient. Safety and tolerability were assessed through community-based follow-up by study teams on days 3 and 7.

**Findings:**

Between October 2023 and September 2024, 800 patients were enrolled: 626 (78.3%) received PQ7, 148 (18.5%) PQ14, and 26 (3.3%) PQ8W. Of those patients who did not withdraw, 97.8% (773/790) were followed up on day 3 and 97.7% (765/783) on day 7. A total of 27 adverse events of special interest (AESIs) were reported in 26 patients; the risk of an AESI was 2.7% (17/626) of patients treated with PQ7, 4.1% (6/148) of patients treated with PQ14 and 11.5% (3/26) of patients treated with PQ8W. Overall, 24 AESIs (88.9%) were related to haemolysis, with the majority (n = 21) due to a fall in haemoglobin >3 g/dL, all without clinical compromise. Serious adverse events (SAEs) occurred in 16 (2.0%) patients, mostly gastrointestinal intolerance (68.8%; 11/16) in patients receiving PQ7. Of the 626 patients treated with PQ7, 9 (1.4%) experienced SAEs that were probably or possibly related to primaquine.

**Interpretation:**

The implementation of G6PD testing to guide primaquine treatment of patients with *P. vivax* malaria was feasible and the primaquine regimens had acceptable safety profiles. The study paves the way for large scale implementation studies of the intervention.

**Funding:**

UNITAID and Australian National Health and Medical Research Council.

**Research in context**

**Evidence before this study**

Primaquine remains the first-line treatment of *Plasmodium vivax* malaria in most endemic countries, usually prescribed at a low total dose (3.5 mg/kg) administered over 14 days. A recent systematic review identified 23 antimalarial clinical trials published between January 1, 2000, and June 8, 2023, that assessed the anti-relapse efficacy and safety of primaquine in patients with *P. vivax* malaria. A total of 6879 patients from 23 trials were included in two individual patient data meta-analyses. In the efficacy analysis, a high total dose (7 mg/kg) of primaquine reduced the cumulative incidence of recurrences by 55% compared to the low-dose (3.5 mg/kg) regimen. The same high total dose of primaquine (7 mg/kg) administered over 7 days had similar efficacy to the same total dose over 14 days. In the safety analysis, a high daily dose of primaquine (1 mg/kg/day) had a low risk of haemolysis in G6PD normal patients, but there was a greater risk of gastrointestinal adverse events compared to those patients treated with 0.25–0.5 mg/kg/day. In 2024 the World Health Organization revised its antimalarial treatment guidelines to recommend a high total dose of primaquine administered over 7 or 14 days for patients with *P. vivax* malaria after screening for G6PD deficiency. To date, no studies have assessed the safety, tolerability and feasibility of high dose primaquine in routine clinical practice.

**Added value of this study**

The SCOPE study was conducted at public health clinics in Indonesia and Papua New Guinea to assess the feasibility, safety and cost effectiveness of a revised case management package for the radical cure of patients with *P. vivax* malaria. Patients were screened for G6PD deficiency using the STANDARD G6PD point of care test, and treated with a high total dose of primaquine (7 mg/kg) prescribed over 7 days, 14 days or 8 weeks, dependent upon the patients’ G6PD activity. This paper presents the results of Stage 1 of the study, which focuses on the safety of high daily dose primaquine. The study highlights that G6PD testing prior to prescription of high dose primaquine is feasible. Large absolute falls in haemoglobin occurred infrequently in patients with high haemoglobin concentration at presentation, but there were no serious haemolytic events in G6PD normal patients. The most frequent serious adverse events were due to gastrointestinal intolerability reported in patients treated with the high daily dose primaquine regimen, almost all of which occurred in a site with food insecurity.

**Implications of all the available evidence**

The evidence from this study supports the 2024 revision of the WHO guidelines recommending the use of a high total dose of primaquine and highlights the haemolytic safety of the 1 mg/kg/day primaquine regimen for the radical cure of *P. vivax* in patients with G6PD activity >70%. On review of the data, the Indonesian and PNG Ministries of Health approved continuation of the study to Stage 2, in which the intervention will be rolled out to 10 clinics across Indonesia and PNG, delivered by routine clinic staff. However, in sites with low food security, an option will be given to split the daily dose of primaquine into a twice daily regimen to reduce gastrointestinal side effects.

## Introduction

*Plasmodium vivax* remains a significant global health challenge causing between 6 and 9 million cases of malaria each year. Outside of sub-Saharan Africa, *P. vivax* has become the predominant cause of malaria, associated with significant morbidity and mortality.^1^ *P. vivax* is more difficult to eliminate than *Plasmodium falciparum* because it forms dormant liver stages (hypnozoites) that can reactivate weeks to months after the initial infection, resulting in recurrent blood stage infections, known as relapses. The frequency and number of relapses vary with geographical location and host immunity.^2^ The risk of early relapse is particularly high in tropical areas, with subsequent relapses occurring every 3–4 weeks. These relapses result in a higher cumulative risk of anaemia making patients more susceptible to concomitant infections such as pneumonia and diarrhoea and increase the risk of mortality.^3^

*P. vivax* radical cure refers to the administration of a combination of antimalarial drugs to target both the blood-stage parasites that cause acute febrile illness and the liver-stage parasites that give rise to relapsing infection. The 8-aminoquinoline compounds, primaquine and tafenoquine, are the only licensed antimalarial drugs that kill hypnozoites and thus prevent subsequent relapses of *P. vivax*. Primaquine has been the primary treatment for preventing *P. vivax* relapses for over 70 years, but its effectiveness is hindered by suboptimal dosing, poor adherence of patients to prolonged treatment courses (typically 14 days), and the reluctance of healthcare providers to prescribe the drug due to the risk of severe haemolysis in G6PD-deficient patients.^4^^,^^5^ Higher total doses of primaquine reduce the risk of recurrence but increase the risk of adverse events.^6^^,^^7^ Haemolytic reactions can be reduced by screening patients for G6PD deficiency prior to administration of primaquine or tafenoquine; however, in resource-limited settings, routine point-of-care G6PD testing is rarely available.^5^ In these areas, the risk of severe adverse drug reactions needs to be balanced against the benefit of reducing the morbidity associated with recurrent episodes of *P. vivax* malaria.

Tafenoquine was prequalified by the World Health Organization (WHO) in 2024 for the radical cure of malaria. It is prescribed as a single dose and thus has potential to overcome issues of adherence inherent to prolonged primaquine regimens. However, tafenoquine is only recommended in combination with chloroquine and in patients with >70% G6PD enzyme activity.

In 2024, the World Health Organization (WHO) updated its antimalarial treatment guidelines to recommend a higher total dose of primaquine for radical cure of *P. vivax* malaria (7 mg/kg) and also endorsed a 7-day regimen to improve adherence.^8^ Delivery of the high-dose, short-course primaquine regimen requires a daily dose of 1 mg/kg/day and is associated with an increased risk of haemolysis, hence it is currently only recommended in patients with >70% G6PD activity. In January 2025, the WHO prequalified a novel point-of-care quantitative assay to diagnose G6PD deficiency; STANDARD G6PD (SD Biosensor, Republic of Korea).^9^ The availability of the STANDARD G6PD in *P. vivax* endemic countries will facilitate safer and more effective radical cure, including single-dose tafenoquine and high-dose primaquine regimens.

Malaria remains highly endemic in parts of Indonesia and PNG. The governments of both countries have committed to eliminating the disease by 2030. This ambitious goal will require innovative strategies to ensure widespread use of safe and effective radical cure. Artemisinin combination therapy (ACT) is the first-line treatment for uncomplicated malaria due to all species in both Indonesia and PNG, because of high rates of chloroquine resistant *P. vivax* and *P. falciparum*.^10^ Since tafenoquine can only be administered with chloroquine it is not currently an option for radical cure in either country. High-dose primaquine over seven days (PQ7) can be given in combination with an ACT and has the potential to improve adherence and effectiveness of radical cure, thus reducing recurrent parasitaemia, associated morbidity and mortality, and ongoing transmission of the parasite.^11^

The SCOPE study (Short COurse PrimaquinE after G6PD testing for the radical cure of *P. vivax* malaria) aims to evaluate a revised case management package for the radical cure of patients with *P. vivax* malaria in Indonesia and PNG. This paper presents the results of Stage 1 of this study, which focuses on the safety of this case management package.

## Methods

### Study overview

The SCOPE study is a staged, pragmatic, binational, multicentre, before-and-after implementation study to determine the safety, feasibility, and cost-effectiveness of a revised package of case management interventions for improved *P. vivax* radical cure. The study was conducted at sites in Indonesia and PNG, with diverse transmission intensity, with the ultimate objective being to generate evidence that would inform the implementation of safe and effective radical cure regimens in other *P. vivax* endemic sites. The interventions include: i) pre-treatment testing of patients for G6PD deficiency using a semi-quantitative point-of-care device STANDARD G6PD from SD Biosensor (Republic of Korea); ii) prescription of one of three different primaquine regimens dependent upon the patients' G6PD activity; iii) improved patient education processes (Supplementary File 1); iv) routine community-based review on days 3 and 7, and v) enhanced malariometric surveillance and community pharmacovigilance. The full SCOPE Study protocol is available online.^12^

### Study sites

**Fig. 1:**
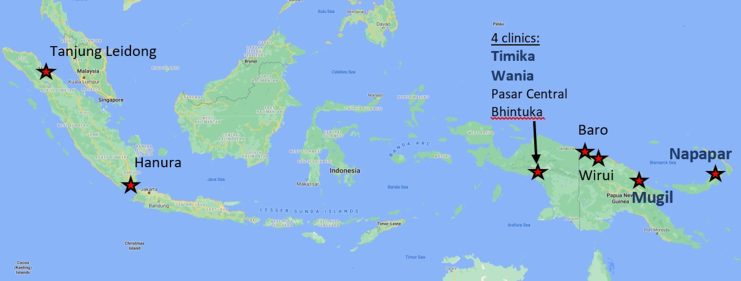
**Locations of the SCOPE study clinics.** Footnote: The four clinics participating in Stage 1 are shown in bold.

Stage 1 of the SCOPE study was conducted at two community health clinics in Indonesia (Timika and Wania clinics) and two in PNG (Mugil and Napapar clinics) (Fig. 1). In the year preceding the start of the study, the total annual case workload across the four clinics was 15,827 cases (Supplementary Table S1). These clinics were purposively selected based on their high number of endemic *P. vivax* malaria cases, adequate staffing, existing research capacity and ability to provide strong safety oversight.

### Enrolment and study procedures

Patients with *P. vivax* malaria were enrolled sequentially at the four clinics. To ensure rigorous adherence to safety protocols, a maximum of 4–6 patients per day were recruited depending on multiple factors affecting each clinic’s capacity. Eligible participants were older than six months or weighed more than 5 kg in Indonesia or older than one year in PNG, who had uncomplicated *P. vivax* mono- or mixed-species infections confirmed by microscopy in Indonesia or rapid diagnostic test (RDT) in PNG according to standard clinic procedures. In Indonesia, microscopy was performed by trained microscopists as part of routine clinical practice. Pregnant or lactating women nursing infants (under six months in Indonesia or one year in PNG), patients with severe malaria or who had a previous severe reaction to primaquine were excluded. Eligible patients were provided details of the study and enrolled after they or their guardians provided written consent. For participants under 12 years of age, informed consent was provided by a parent or legal guardian. For those aged 12 to under 18 years, consent was similarly obtained from a parent or legal guardian, along with the participant's assent (Supplementary File 2).

Following informed consent, patients’ G6PD activity and haemoglobin (Hb) were measured using the STANDARD G6PD. Those with a Hb concentration of less than 8 g/dL (measured using the STANDARD G6PD) were excluded from the study. All study procedures were conducted by the study team.

Patients excluded from the study were treated according to standard national protocols. Baseline data were recorded from all patients enrolled, including demographics, *Plasmodium* species of infection (determined using light microscopy or RDT), G6PD activity and Hb concentration.

### Treatment

Patients were treated with high total dose primaquine using different regimens determined by their G6PD activity. Those with G6PD activity ≥6.1 U/gHb (corresponding to ‘normal’ enzyme activity, >70%) were prescribed high-dose (1.0 mg/kg/day) primaquine for 7 days (PQ7), those with activity between 4.1 and 6.0 U/gHb (corresponding to ‘intermediate’ enzyme activity, 30–70%) were prescribed 0.5 mg/kg/day of primaquine for 14 days (PQ14) and those with activity ≤4.0 U/gHb (corresponding to ‘deficient’ enzyme activity, <30%) were prescribed 0.75 mg/kg primaquine once a week for 8 weeks (PQ8W). Whilst in the clinic, the first doses of primaquine and ACT (artemether-lumefantrine in PNG and dihydroartemisinin-piperaquine in Indonesia) were supervised and the patients were provided with education on malaria, the side effects of primaquine, and the importance of taking primaquine with food and completing a full course of treatment. The protocol procedures are presented in Supplementary Table S2.

### Monitoring for adverse events

Community-based follow-up assessments were conducted by a study nurse either at the patient’s home or at the clinic. These reviews took place between days 3 and 5 (termed ‘day 3 review’) and between days 6 and 10 (termed ‘day 7 review’) of treatment. G6PD activity and Hb concentration were measured again on both days. At each follow-up visit, a study nurse completed a two-page clinical record form and assessed the severity of symptoms, looking for signs of three key potential adverse events of special interest (AESI): acute haemolysis, gastrointestinal upset, and methaemoglobinaemia. The severity of adverse events was graded according to the NCI Common Terminology Criteria for Adverse Events (CTCAE vs. 5.0).^13^ A haemolytic AESI was defined by the presence of one or more of the following: i) grade 3 or 4 fatigue, breathlessness or dizziness, ii) severe pallor or jaundice, iii) dark urine (Hillmen urine colour score > 7), iv) a fall in Hb from baseline >3 g/dL, and/or v) a fall in Hb to <7 g/dL. A gastrointestinal AESI was defined as grade 3 or 4 abdominal pain, nausea, anorexia or vomiting. A methaemoglobinaemia AESI was defined as methaemoglobin concentration >10% associated with grade 3 or 4 breathlessness or dizziness.

Serious Adverse Events (SAEs) were defined as any untoward medical occurrence, regardless of cause, that occurred after the commencement of primaquine treatment and within 7 days of completion and met at least one of the following criteria: resulted in death, was life threatening, required inpatient hospitalisation or prolonged existing hospitalisation, resulted in persistent or significant disability/incapacity, was a congenital anomaly/birth defect or required medical intervention to prevent permanent impairment or damage.^14^

At the day 3 and day 7 reviews, any patient with signs or symptoms meeting the definition for an AESI or SAE, or in whom there was clinical concern, were ‘flagged’ and referred to the clinic for a health practitioner-led clinical review (Supplementary File 3) or referred directly to the hospital for medical management if appropriate.

### Sample size

The sample size of 800 patients for stage 1 was based on an assumption that 764 (95.5%) of these patients would have a G6PD activity >70% and be eligible for PQ7, and that the proportion of patients receiving high-dose short-course primaquine who had to stop therapy within 7 days would be 5%. This sample size would provide the true proportion of patients needing to stop treatment with a 95% confidence interval of ±1.55%.

### Statistical analysis

The statistical analysis was conducted according to an a *priori* statistical analysis plan (Supplementary File 4 which outlines the analysis of Stage 1 and Stage 2). Baseline characteristics were summarised using descriptive statistics. Continuous variables were presented as medians with interquartile ranges (IQR), and categorical variables were expressed as frequencies and percentages. Safety outcomes included the proportion of patients who experienced at least one SAE or AESI (with further categorisation into haemolysis, gastrointestinal intolerance, and/or methaemoglobinaemia-related events), as well as the proportion of patients who permanently discontinued primaquine before the end of treatment. Study performance and feasibility were assessed by presenting the proportion of patients (95% Confidence Interval (CI)) who received the correct treatment based on G6PD activity and who completed clinical reviews on days 3 and 7.

Protocol deviations were quantified and categorised as being related to prescription errors, administration of primaquine to ineligible patients (i.e., pregnant or breastfeeding women or infants), or patients with incomplete follow-up. The following Hb and G6PD-related outcomes were quantified: the mean changes in Hb from baseline on days 3, and 7, the maximum absolute fall in Hb from baseline within the first ten days, and the number and proportion of patients with clinically significant reductions in haemoglobin (Hb fall >3 g/dL, or Hb fall >25% to a concentration of <7 g/dL). These quantitative endpoints were analysed overall and stratified by clinic and primaquine treatment regimen. All statistical analyses were performed using Stata Statistical Software (Release 18, StataCorp, Texas, USA).

### Ethical considerations

The intervention package represented improvements to existing practices that were expected to reduce the risk of recurrent *P. vivax* malaria through improved effectiveness of primaquine radical cure and a reduced risk of adverse events due to pre-treatment G6PD testing and the day 3 clinical review. If the intervention package was demonstrated to be feasible in remote rural clinics, the expectation was that a similar intervention could be rolled out more broadly across malaria-endemic areas in both countries. Prior to commencing the study, community leaders, including chiefs and religious leaders were engaged and permission to introduce the new strategies in their respective communities was obtained. Community education and awareness sessions were conducted by the study and health facility teams to increase awareness of *P. vivax* malaria and the need for more effective case management approaches and community-based follow-up. All patients enrolled in the Stage 1 safety study were made aware of the potential benefits and risks of the short-course high-dose primaquine regimen and only those who provided written informed consent were enrolled into the study.

The study protocol was approved by the World Health Organization Ethics Committee (Indonesia: ERC 0003810, PNG: ERC 0003892), Menzies School of Health Research (HREC: 2023-4524), Alfred Health (Burnet Institute) (Project No: 18/23), University of Gadjah Mada (KE/FK/0079/EC/2023), University of Indonesia (KET-347/UN2.F1/ETIK/PPM.00.02/2023), University of North Sumatera (80/KEPK/USU/2023), Institute of Tropical Medicine (1655/23), PNG Institute of Medical Research (PNGIMR) (IRB: 22.02), and the PNG National Department of Health Medical Research Advisory Committee (MRAC: 22.66). The study was registered on clinicaltrials.gov for Indonesia (NCT05879224) and PNG (NCT05874271).

### Role of the funding source

The funders of the study had no role in study design, data collection, data analysis, data interpretation, or writing of the report.

## Results

### Screening and enrolment

**Fig. 2:**
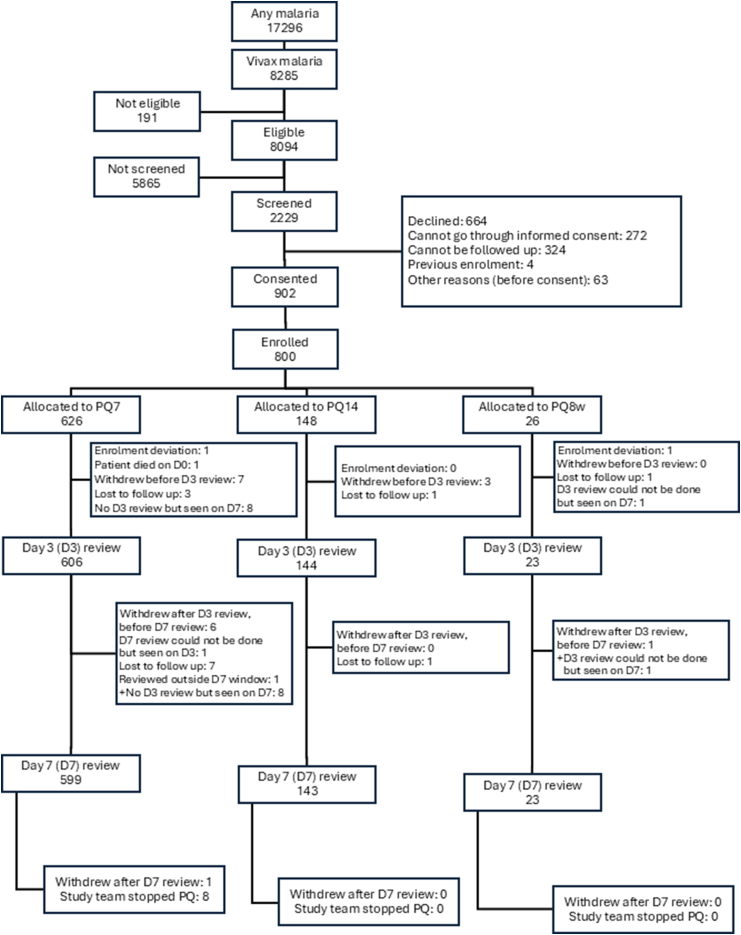
**Study enrolment**.

Between 11th October 2023 and 9th September 2024, a total of 8285 patients with *P. vivax* malaria (mono or mixed species infection) presented to one of the four study clinics, of whom 2229 (26.9%) were screened for enrolment and 800 (9.7%) were enrolled into the study. The study profile and reasons for exclusion are presented in Fig. 2.

### Baseline patient characteristics

Of the 800 patients enrolled in the study, 459 (57.4%) were male, and the median age was 18.0 years (IQR: 10.0–29.0 years). In PNG 57.7% (173/300) of patients enrolled were children 15 years of age or younger compared to 34.8% (174/500) in Indonesia. All patients in Indonesia were diagnosed by microscopy, with *P. vivax* mono-infection identified in 88% (440/500) of patients and mixed species infections in 12% (60/500). In PNG, where patients were diagnosed using an RDT, 19.7% (59/300) of infections due to non-*P. falciparum* infection and 80.3% (241/300) *P. falciparum* plus non-*P. falciparum* infections.

Overall, 626 (78.3%) patients had normal G6PD activity (≥6.1 U/gHb), 148 (18.5%) had intermediate G6PD activity (4.1–6.0 U/gHb), and 26 (3.3%) were G6PD deficient (≤4.0 U/gHb); Table 1. The proportion of patients with intermediate G6PD activity was 26.8% (134/500) in Indonesia and 4.7% (14/300) in PNG; overall 67.6% (100/148) of patients with intermediate activity were male (Table 1). The overall median Hb concentration at enrolment was 12.3 g/dL (IQR: 10.8–14.0) and was lower in patients enrolled in PNG (11.2, IQR: 9.8–12.6 g/dL) compared to those in Indonesia (13.1, IQR: 11.5–14.6 g/dL). In PNG, 27.7% (83/300) of patients had moderate anaemia (Hb < 10 g/dL) compared to 7.6% (38/500) in Indonesia. The median Hb concentration measured using the STANDARD G6PD (SD Biosensor) at enrolment was 12.0 (IQR: 10.4–13.5) g/dL in patients receiving PQ7, 13.6 (IQR: 12.2–14.9) g/dL in patients receiving PQ14, and 13.0 (10.9–14.6) g/dL in patients receiving PQ8W.

**Table 1:** Baseline characteristics of patients enrolled in the study by health facility site.

	Overall	Timika	Wania	Napapar	Mugil
Total enrolled	N = 800	N = 258	N = 242	N = 150	N = 150
Age (years), Median (IQR)	18.0 (10.0–29.0)	21.0 (12.0–31.0)	23.0 (12.0–32.0)	12.0 (6.0–25.0)	13.0 (9.0–19.0)
Age N (% of all enrolled patients)					
<5 yrs	60 (7.5%)	12 (4.7%)	8 (3.3%)	27 (18.0%)	13 (8.7%)
5–15 yrs	287 (35.9%)	82 (31.8%)	72 (29.8%)	55 (36.7%)	78 (52.0%)
>15 yrs	453 (56.6%)	164 (63.6%)	162 (66.9%)	68 (45.3%)	59 (39.3%)
Sex N (% of all enrolled patients)					
Male	459 (57.4%)	152 (58.9%)	141 (58.3%)	78 (52.0%)	88 (58.7%)
Female	341 (42.6%)	106 (41.1%)	101 (41.7%)	72 (48.0%)	62 (41.3%)
Malaria diagnosis N (% of all enrolled patients)					
*Pv* mono infection (Microscopy)	440 (55.0%)	241 (93.4%)	199 (82.2%)	0 (0.0%)	0 (0.0%)
*Pv* mixed infection (Microscopy)	60 (7.5%)	17 (6.6%)	43 (17.8%)	0 (0.0%)	0 (0.0%)
*Pv* mono/mixed (RDT)	300 (37.5%)	0 (0.0%)	0 (0.0%)	150 (100.0%)	150 (100.0%)
Total *Pv* (mono or mixed)	800 (100.0%)	258 (100.0%)	242 (100.0%)	150 (100.0%)	150 (100.0%)
G6PD value N (% of all enrolled patients)					
Normal (≥6.1 U/gHb)	626 (78.3%)	166 (64.3%)	181 (74.8%)	134 (89.3%)	145 (96.7%)
Intermediate All (4.1–6.0 U/gHb)	148 (18.5%)	77 (29.8%)	57 (23.6%)	14 (9.3%)	0 (0.0%)
Intermediate Males	100 (12.5%)	53 (20.5%)	40 (16.5%)	7 (4.7%)	0 (0.0%)
Intermediate Females	48 (6.0%)	24 (9.3%)	17 (7.0%)	7 (4.7%)	0 (0.0%)
Deficient (≤4.0 U/gHb)	26 (3.3%)	15 (5.8%)	4 (1.7%)	2 (1.3%)	5 (3.3%)
Hb Concentration, Median (IQR)					
All	12.3 (10.8–14.0)	13.0 (11.4–14.4)	13.2 (11.8–14.6)	12.4 (11.2–13.7)	10.1 (9.2–11.1)
Patients treated with PQ7	12.0 (10.4–13.5)	12.5 (10.9–14.2)	12.9 (11.5–14.2)	12.4 (11.2–13.7)	10.1 (9.2–11.1)
Patients treated with PQ14	13.6 (12.2–14.9)	13.4 (12.2–14.7)	14.3 (12.7–15.2)	12.4 (11.2–13.6)	.
Patients treated with PQ8W	13.0 (10.9–14.6)	13.8 (12.3–14.7)	11.9 (10.8–14.4)	13.7 (13.5–13.8)	10.0 (9.1–11.7)
Treatment N (% of all enrolled patients)					
PQ7	626 (78.3%)	166 (64.3%)	181 (74.8%)	134 (89.3%)	145 (96.7%)
PQ14	148 (18.5%)	77 (29.8%)	57 (23.6%)	14 (9.3%)	0 (0.0%)
PQ8W	26 (3.3%)	15 (5.8%)	4 (1.7%)	2 (1.3%)	5 (3.3%)

### Study performance metrics

As per the study protocol, all patients enrolled had *P. vivax* malaria (either alone or mixed with another species), were neither pregnant nor lactating, and not infants (<6 months old in Indonesia or <1 year old in PNG). All participants were prescribed the correct treatment and dose of primaquine according to their G6PD activity. Protocol deviations are presented in Supplementary Table S3.

Three patients (one in Wania, Indonesia and two in Mugil, PNG) were incorrectly enrolled into the study despite meeting the study exclusion criterion of having a baseline Hb < 8 g/dL as measured by the STANDARD G6PD. The Indonesian patient completed their PQ treatment whereas the two PNG patients had treatment discontinued by the study team on day 3. All three participants were followed until day 7 with no adverse events reported.

A total of 18 (2.3%) participants withdrew from the study (Fig. 2). The rate of withdrawal was 2.2% (14/626) [95% CI: 1.2, 3.7%] in those treated with PQ7, 2.0% (3/148) [0.4, 5.8%] with PQ14, and 3.8% (1/26) [0.1, 19.6%] with PQ8W. Of the participants who did not withdraw, 97.8% (773/790) were reviewed on day 3 (range: 3–5 days), and 97.7% (765/783) were reviewed on day 7 (range: 6–10 days). An additional 8 patients (1.0%), all treated with PQ7, had primaquine ceased by the study team before completion of treatment, due to adverse events or other comorbidities, including occurrence of a haemolytic AESI (two patients), high methaemoglobinaemia (two patients), gastrointestinal SAE (two patients), and one female who was later identified as being four weeks pregnant with worsening gastrointestinal symptoms. Overall, 29 patients 3.6% (29/800) prematurely ceased primaquine prior to the end of their prescribed treatment course.

### Haematological profiles

**Fig. 3:**
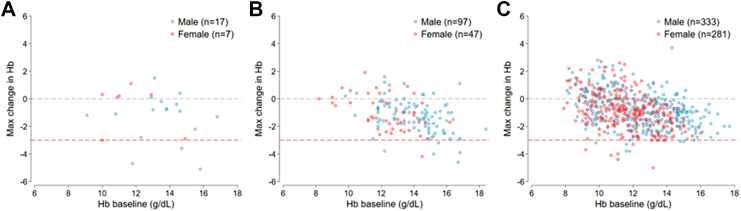
**Maximum change in haemoglobin (g/dL) from baseline during the first 10 days for patients with G6PD deficiency treated with PQ8W (A), patients with intermediate G6PD activity treated with PQ14 (B), and patients with normal G6PD activity treated with PQ7 (C).** Footnote: The maximum change in haemoglobin was defined as the change from Hb at baseline to the minimum recorded Hb between day 3 and day 10. Positive change indicates a rise in haemoglobin from baseline. Red horizontal dotted lines reflect the threshold used in this study to define haemolysis – a reduction in Hb > 3 g/dL.

The mean maximum change in Hb during the first 10 days of treatment was −0.8 g/dL (range: −5.0 to 3.7) in the PQ7 group, −1.2 g/dL (range: −4.6 to 1.9) in the PQ14 group, and −1.2 g/dL (range: −5.1 to 1.5) in the PQ8W group; Fig. 3. The maximum change in Hb during follow-up according to the patient’s G6PD activity is presented in Supplementary Fig. S1.

### Adverse events of special interest

Of the patients who had routine follow-up, 2.8% (22/773) required practitioner-led review at day 3, and 1.5% (12/765) at day 7; Table 2. Overall, the risk of requiring any practitioner-led review was 3.6% (22/615) in the PQ7 arm, 4.2% (6/144) in the PQ14 arm and 12.5% (3/24) in the PQ8W arm. A total of 27 AESIs were reported in 26 patients. A 5-year-old female from Napapar, PNG treated with PQ7 had both a haemolytic and gastrointestinal AESI. Two AESIs were detected between days 0–2, 17 AESIs were detected between days 3–5, and 8 AESIs were detected between days 6–9. In total 4 of the 26 patients with an AESI were not reviewed by a practitioner, all of whom had haemolytic AESIs and none were clinically compromised. The risk of having an AESI was 2.9% (18/626) in patients treated with PQ7, 4.1% (6/148) in patients treated with PQ14, and 11.5% (3/26) in patients treated with PQ8W.

**Table 2:** Number of patients with at least one event by treatment arm.

	Overall	PQ7	PQ14	PQ8W
Total enrolled	N = 800	N = 626	N = 148	N = 26
Patients with day 3 (D3) review	773 (96.6%)	606 (96.8%)	144 (97.3%)	23 (88.5%)
Flagged day 3–5 N (%)^a^	22 (2.8%)	16 (2.6%)	5 (3.5%)	1 (4.2%)
Patients with day 7 (D7) review	765 (95.6%)	599 (95.7%)	143 (96.6%)	23 (88.5%)
Flagged day 6–10 N (%)^a^	12 (1.5%)	7 (1.1%)	3 (2.1%)	2 (8.3%)
Other day flagged N (%)^a^	1 (0.1%)	0 (0.0%)	0 (0.0%)	1 (4.2%)
Adverse event of special interest (AESI) N (%)^a^				
Haemolysis N (%)				
AESI (SAE)	4 (0.5%)	4 (0.7%)	0 (0.0%)	0 (0.0%)
AESI (non-SAE)	20 (2.6%)	11 (1.8%)	6 (4.2%)	3 (12.5%)
Total	24 (3.1%)	15 (2.4%)	6 (4.2%)	3 (12.5%)
Gastrointestinal N (%)				
AESI (SAE)	3 (0.4%)	3 (0.5%)	0 (0.0%)	0 (0.0%)
AESI (non-SAE)	0 (0.0%)	0 (0.0%)	0 (0.0%)	0 (0.0%)
Total	3 (0.4%)	3 (0.5%)	0 (0.0%)	0 (0.0%)
Methaemoglobinaemia N (%)				
AESI (SAE)	0 (0.0%)	0 (0.0%)	0 (0.0%)	0 (0.0%)
AESI (non-SAE)	0 (0.0%)	0 (0.0%)	0 (0.0%)	0 (0.0%)
Total	0 (0.0%)	0 (0.0%)	0 (0.0%)	0 (0.0%)
Serious adverse events (SAEs) N (%)^b^				
SAE – Related (Possibly, Probably, Definitely)	9 (1.1%)	9 (1.4%)	0 (0.0%)	0 (0.0%)
SAE – Unrelated (not related, unlikely)	7 (0.9%)	7 (1.1%)	0 (0.0%)	0 (0.0%)
Total	16 (2.0%)	16 (2.6%)	0 (0.0%)	0 (0.0%)
Related SAE, which is also an AESI	2 (0.3%)	2 (0.3%)	0 (0.0%)	0 (0.0%)
Related SAE Other	7 (0.9%)	7 (1.1%)	0 (0.0%)	0 (0.0%)

The AESI data stratified by country are presented in Supplementary Table S4.

a Denominator is the number of patients reviewed by the study team at any day (Overall N = 783; PQ7 N = 615; PQ14 N = 144 PQ8W N = 24).

b Denominator is the number of patients enrolled and treated with the primaquine regimen.

Overall, 24 (88.9%) of the 27 AESIs were related to haemolysis, of which 15 (62.5%) were detected within three days of starting primaquine (see Supplementary Table S5). Twenty-one patients experienced a fall in Hb of greater than 3 g/dL (mean 3.9 g/dL, range 3.1–5.1 g/dL), of whom 4 subsequently fell to below 7.0 g/dL and two decreased by ≥5.0 g/dl. None of these 21 patients reported severe (grade 3 or 4) clinical symptoms or clinical compromise. All but two of these 21 patients continued taking primaquine, completed treatment and had a recovery of haemoglobin within 7 days of treatment. Of the remaining patients with haemolytic AESIs, one had a fractional Hb drop of >25%, falling below 7 g/dL (from 9.3 to 6.4 g/dL) and one experienced grade 3 fatigue on day 9 without any fall in Hb. One patient reported grade 3 breathlessness and was subsequently hospitalised with a diagnosis of dengue haemorrhagic fever and community-acquired pneumonia. Over the course of the illness, the patient’s haemoglobin decreased by 0.7 g/dL. Following discharge from the hospital, the patient continued PQ7 therapy and completed the course without further clinical issues.

Three AESIs were related to gastrointestinal intolerability, all of the affected patients had been treated with PQ7; Supplementary Table S6. One 24-year-old male from Indonesia, reported recurrent vomiting on day 0 that started before commencing primaquine treatment. The patient was hospitalised later on the same day, and the event was categorised as a serious adverse event (SAE). One 13-year-old male experienced grade 4 abdominal pain on day 1. Another 5-year-old female from PNG had grade 3 nausea and vomiting on day 8, resulting in treatment cessation. No patients had an AESI related to methaemoglobinaemia.

### Serious adverse events (SAE)

Sixteen (2.0%) patients experienced one or more SAE, all of whom were treated with PQ7; Supplementary Table S7. Two cases were deemed to be probably related to treatment, seven possibly related, four unlikely related, and three unrelated. None of the patients had a SAE attributable to haemolysis.

#### Gastrointestinal SAE

Eleven (1.8%) of the 626 patients treated with PQ7 experienced gastrointestinal SAEs. Three of these patients, all from PNG, had symptoms that began after completing PQ7 and were considered unlikely to be related to primaquine. Of the eight patients with gastrointestinal SAEs related to PQ, one was an adult male from Indonesia who presented with vomiting and nausea, and vomited again after his first PQ7 dose. He was subsequently admitted on day 0 for intravenous fluids and the event was considered an AESI and SAE and probably exacerbated by primaquine. The other seven gastrointestinal SAE cases related to primaquine occurred in PNG, of whom one was a child (<15 years old) who presented with grade 3 gastrointestinal symptoms and moderate dehydration requiring admission for intravenous fluids. Three of these children had already completed their prescribed PQ7 treatment within the preceding 4 days and the other had completed 5 out of 7 doses of PQ7. The remaining three primaquine related gastrointestinal SAEs occurred in adults, two of whom had completed PQ7 treatment and one who had completed 5 doses.

#### Methaemoglobinaemia SAE

One 31-year-old female had an SAE related to methaemoglobinaemia associated with an oxygen saturation of 92% on air and a methaemoglobin concentration of 16.3%. She presented at routine follow-up on day 7, with a dry cough and grade 1 epigastric pain, and thus did not qualify as an AESI. She had completed her PQ7 regimen but was hospitalised on day 8 when her oxygen saturation fell to 88% on room air, although she had no associated symptoms such as dizziness or breathlessness. She was discharged after 5 days with diagnoses of acute pharyngitis, dyspepsia, and methaemoglobinaemia which was classified as probably related to primaquine.

One study participant died during the study. The patient was a 13-year-old female from Indonesia, diagnosed with *P. vivax* mono-infection with a parasitaemia of 14,400/μL, Hb concentration of 10.2 g/dL, and normal G6PD activity (7.7 U/gHb) on the day of malaria diagnosis. She was prescribed primaquine 60 mg daily (0.99 mg/kg) and dihydroartemisinin-piperaquine for three days. Her first doses of both medications was administered at the clinic, and she was also prescribed domperidone 10 mg three times daily. On the same day, 8 h after taking her antimalarial medications, she was taken to the local district hospital by her family and was pronounced dead on arrival. According to the family, she had experienced sudden dizziness or a headache before losing consciousness. A verbal autopsy was conducted, but a physical autopsy and postmortem evaluation could not be conducted due to cultural reasons. The study Safety Review Team and an independent clinical review concluded that the event was not related to primaquine toxicity; the likely cause was determined to be either a cardiac arrhythmia or a cerebral vascular haemorrhage (Supplementary File 5).

## Discussion

Primaquine has been used for the radical cure of *P. vivax* and *Plasmodium ovale* malaria for over 70 years. Its tolerability profile is well described and includes three important potential adverse effects: drug-induced haemolysis, gastrointestinal intolerance, and methaemoglobinaemia. Historically, the greatest concern amongst healthcare providers relates to severe haemolysis in patients with G6PD deficiency. The availability of a handheld device to test G6PD activity now allows point-of-care diagnosis of patients at risk of haemolysis so that the appropriate dose of primaquine can be prescribed. Whilst higher daily doses of primaquine are also associated with gastrointestinal symptoms, this can be mitigated by encouraging patients to take their medication with food.

The Stage 1 SCOPE study aimed to investigate the safety of a novel intervention package to improve the effectiveness of primaquine radical cure in four remote locations in Papua, Indonesia and PNG. Our findings highlight that whilst there were significant challenges in implementing the revised care package at local health facilities, well-trained health staff delivered appropriate care, with no prescribing errors and a high proportion of patients receiving early clinical review.

A total of 27 (3.4%) AESIs were reported; a risk of 2.9% in patients treated with PQ7, 4.1% in those treated with PQ14 and 11.5% following PQ8W. Almost all of these events were related to haemolysis, according to a laboratory definition of a fall in Hb > 3 g/dL. Following the treatment of acute malaria, attributing decreases in haemoglobin to drug or parasite-induced haemolysis is challenging. A large fall in Hb (>3 g/dL) has been used as a marker of severe primaquine haemolysis. Patients experiencing the greatest acute falls in haemoglobin are generally those initially presenting with a high haemoglobin concentration.^15^ We observed a similar pattern in the SCOPE study; however, patients with >3 g/dL haemoglobin drops had minimal or no associated clinical symptoms and showed rapid haemoglobin recovery, even when primaquine was continued. The fall in haemoglobin concentrations in patients treated with PQ7 were similar to those observed in patients treated with PQ14, however there was a greater risk of haemolysis in G6PD deficient patients treated with the weekly primaquine regimen recommended by WHO. The risk of haemolysis increases substantially as G6PD activity falls below 30%, and can be particularly severe with certain variants.^4^ The risks of haemolysis in G6PD deficient patients even with the weekly regimen of primaquine, has been observed previously, and highlights the need for caution and early clinical review when prescribing primaquine to these vulnerable patients.^16^^,^^17^

Serious adverse events were reported in sixteen patients (Supplementary Table S7), all of whom were treated with PQ7. Nine SAEs were classified as being related to PQ, representing an overall risk after PQ7 of 1.4%, of which 8 cases were associated with gastrointestinal intolerance and one with methaemoglobinaemia. Nearly all (87.5%, 7/8) of the gastrointestinal events occurred in PNG (4 Mugil, 3 Napapar). In these sites, food security was low and likely reflected reduced ability of patients to take their medication with food. Three cases began after day 7, following the completion of primaquine treatment, and may have been due to concomitant gastrointestinal infections, which are frequently reported in children recovering from *P. vivax* malaria.^18^

In a recent systematic review and individual patient data meta-analysis of patients enrolled in more than 27 clinical trials and treated with primaquine, a greater proportion of patients treated with a high daily dose of primaquine (1 mg/kg/day) reported gastrointestinal symptoms, and this was particularly apparent in young children.^6^ In this review, abdominal pain was the most commonly reported event occurring in 1.8% of patients not treated with primaquine, 6.8% of patients treated with 0.5 mg/kg/day primaquine, and 11.6% of patients treated with 1 mg/kg/day primaquine. Gastrointestinal symptoms, in conjunction with the relatively high pill burden required to achieve 1 mg/kg, may contribute to poor adherence and reduce effectiveness of the regimen. Our findings highlight the importance of taking high-dose primaquine with food. However, when food is not readily available, or patients are unable to tolerate food during acute malaria, alternative strategies could be applied, including delaying administration of the first dose of primaquine until the acute symptoms of malaria have abated or splitting the once daily dose to a twice daily regimen.

Primaquine induces oxidative stress, and is associated with a rise in methaemoglobin concentration, which is correlated with better antirelapse efficacy (lower risk of recurrence). In a recent review, 8.7% of patients treated with 1 mg/kg/day primaquine had a methaemoglobin concentration >15%, but these patients were almost all asymptomatic.^19^ In SCOPE Stage 1, no patients met the criteria for a methaemoglobin AESI, although one patient experienced prolonged methaemoglobinaemia and desaturation without significant symptoms.

The risks of primaquine treatment need to be weighed against the benefits of preventing multiple *P. vivax* recurrences.^20^ In PNG and Papua, Indonesia, there is a high risk of hospitalisation following treatment for malaria, particularly in patients not treated with primaquine. In Papua, Indonesia, almost 3% of patients presenting as outpatients with *P. vivax* malaria who were not treated with primaquine subsequently required admission to hospital within 28 days, and half of these cases were related to recurrent episodes of *P. vivax* malaria. The risk of hospital admission was almost halved in patients who were treated with primaquine.^21^ In this context, the current study demonstrated that whilst rates of hospitalisation following the treatment of *P. vivax* malaria were relatively frequent, occurring in 2% of patients, they were lower than expected. The risks of related SAEs and AESIs were also lower than predicted.

Effective implementation of the intervention package required training of clinic staff in G6PD testing, and it took time for them to attain proficiency. Intermediate activity was defined as being between 4 and 6 U/gHb to identify and exclude heterozygous females from being treated with the 1 mg/kg daily dose. Notably 68% (100/148) of the patients with this degree of enzyme activity were males, suggesting a conservative threshold that also detects male individuals at the lower end of the normal distribution. New laboratory procedures needed to be discussed and implemented, and there was a period during which best practices in each clinic were developed. Complementary qualitative studies on the acceptability and feasibility of these processes will be reported separately and will inform expansion of the intervention to new clinics and wider scale-up. Both patients and community health workers required training and education to understand why G6PD testing was required and would result in different treatment regimens. This relied on appropriate communication tools to ensure that the message was well-received and understood so that patients could make informed decisions about their health care.

Stage 1 of the SCOPE study was conducted primarily to demonstrate the safety of implementing high daily dose primaquine regimens in patients following G6PD testing. Despite the comparatively large sample size, follow-up rates were excellent with few protocol deviations. Limitations of the study include the restricted diagnostic capabilities for investigating adverse events potentially unrelated to primaquine. Additionally, it was not possible to clearly distinguish the relative contributions of primaquine and malaria to observed haemoglobin drops.

In conclusion, the Stage 1 SCOPE study provides reassuring evidence on the safety of a high daily dose short-course primaquine regimen for the radical cure of *P. vivax* in patients with G6PD activity >70%. The intervention package included pre-treatment point-of-care G6PD activity assessment, enhanced patient education, early clinical review, and community pharmacovigilance. After review of the data, both the Indonesian and PNG Ministries of Health approved continuation of the study to Stage 2, in which the intervention will be rolled out to 10 clinics across Indonesia and PNG, delivered by routine clinic staff. A key recommendation from Stage 1 was that the intervention package should be revised in sites with low food security, with twice daily primaquine dosing to reduce gastrointestinal side effects. Stage 2 of the SCOPE study is now underway and will determine the feasibility, acceptability, and cost-effectiveness of the intervention package to inform national policy and practice in both countries.

## Contributors

ML, JRP, APP, IS, NMD, LJR and RNP conceived the study. ML, JRP, LJR, RNP, NMD, LF, MOK, MM, EN, JA, YA, CA, IP, SR, FA, VJ, AP, AS, SA, FHB, APH, EK, IL, BL, ER, VSS, and KM were involved in data collection and training. KM, EC, PD provided project administration. MT, RU, MD, AD, CK, TK, JK, NV, TW, EJ, SD, HH, TD, JRP, LF, MS, MM, MOK, LR, RNP, NMD, JA, KM, EC, PD, ER, VSS, provided study oversight. GL,MR, KB, JAS conducted data analysis. ML, JRP, LJR and RNP have accessed and verified the data. LF, MOK, VSS, ER, NMD, developed the first draft of the manuscript, all authors reviewed and approved the final version of the manuscript. LF, MOK, ML, JRP, SD, LJR and RNP were responsible for the decision to submit the manuscript. The corresponding authors had full access to all the data in the study and had final responsibility for the decision to submit for publication.

## Data sharing statement

The SCOPE study protocol is published and available in an open-access article. The statistical analysis plan, informed consent forms and educational material are available in the Supplementary Files. The deidentified data supporting the conclusion of this article will be available for researchers after approval by the study Steering Committee. For all data-sharing enquiries, please contact the corresponding authors at ric.price@menzies.edu.au or leanne.robinson@burnet.edu.au.

## Editor note

The Lancet Group takes a neutral position with respect to territorial claims in published maps and institutional affiliations.

## Declaration of interests

All other authors declare no competing interests.

## Appendix A Supplementary data

Supplementary Fig. S1 and Tables S1–S7

SCOPE Protocol
