# Supplementary material for: High-dose, short-course primaquine after point-of-care G6PD testing for the radical cure of Plasmodium vivax malaria: a safety study in Papua New Guinea and Indonesia
Source: Lancet Reg Health West Pac. 2026 Jun 11;71:101903. doi: 10.1016/j.lanwpc.2026.101903 (PMC13276568; doi:10.1016/j.lanwpc.2026.101903)
Supplement: Supplementary File 1 [file mmc1.pdf]

Education checklist for research staff treating patients

No.	Item	Checklist
1	**Explain the blood and liver stages of *P. vivax* malaria, and why it is important to take primaquine for the length of the prescription:**<br>• *Plasmodium vivax* malaria has blood stage that causes fever and acute symptoms, and liver stage that cause relapse.<br>• Relapses caused by the liver stage may make the patient feel ill in the future and experience complications of malaria, such as anemia, just like the initial acute illness.<br>• The blue tablet(s) DHP are prescribed for 3 days (administered once per day) to kill the blood stage parasites.<br>• Only the small brown tablet primaquine can kill the liver parasites, but to prevent relapse the patient needs to finish the whole treatment.<br>• ***Refer to flip chart image of blood and liver stage in* P. vivax *life cycle***	
2	**Explain why it is important to know your G6PD status:**<br>• Primaquine is an important drug but can cause haemolysis (destroy red blood cells) in patients who have low levels of an enzyme call G6PD.<br>• Primaquine-induced haemolysis can be a life-threatening condition, potentially requiring blood transfusion and dialysis in patients with low G6PD activity.<br>• Knowing the G6PD status of a patient prior to prescribing primaquine can help healthcare workers choose the best treatment for patients and minimize the risk of haemolysis.<br>• The SD Biosensor can determine the G6PD status and can provide results within a few minutes.<br>• ***Refer to flip chart images of haemolysis and G6PD testing***	☐
3	**Explain the changes to *P. vivax* treatment in this study:**<br>• Research has shown that higher primaquine dose (7 mg/kg) is better in killing liver parasites. In this SCOPE study, we are assessing whether taking a higher daily dose of primaquine will reduce the likelihood of malaria relapse.<br>• Before giving primaquine, the clinic staff will obtain a capillary blood sample (one fingerprick test), to test the patient's G6PD level.<br>• If patients have normal G6PD levels, they will be offered a higher primaquine dose than usual for 7 days (1mg/kg/day) - a shorter period than current practice - which should make it easier for the patients to complete the whole course of treatment.<br>Those who have intermediate G6PD levels will receive the same total dose but over 14 days (0.5mg/kg/day), and those who have very low G6PD levels will receive treatment over 8 weeks (0.75mg/kg/week).<br>• G6PD testing and the 7-day primaquine regimen are not currently part of routine practice in Indonesia.	☐

	• ***Refer to flip chart images different treatment, based on G6PD status***	
4	**Explain the drug administration procedure: how many tablets per day, how many times per day, how many days:**<br>• Based on patients' G6PD level, they will be offered primaquine treatment for 7 days, 14 days, or 8 weeks. The total number of tablets recommended will be calculated based on their body weight. (**Drug Administration SOP**).<br>• The tablets should be taken once per day.<br>• On the first day, tablets should be taken at the clinic and observed by the research or clinical team.<br>• If the participant vomits < 30 minutes after ingesting their antimalarial tablets, then the whole dose should be re-administered. If the participant vomits > 30 minutes after ingesting the antimalarial tablets, there is no need to re-administer the tablets.<br>• Patients should contact the research staff to arrange replacement tablets should they be needed (e.g. due to vomiting or loss).<br>• If the participant misses one or more days of antimalarial tablets at home, they should continue to take the next dose of medications as normal. They should not take a double dose to make up for a missed dose.<br>• Patients should complete their entire treatment even if they feel better. Not finishing the course will increase the chance of relapse with malaria again.<br>• ***Refer to flip chart images of treatment, based on their G6PD status, and taking tablets with food***<br>• ***Show treatment card detailing their G6PD status, treatment prescribed, and research staff contact details***	☐
5	**Educate on the possible side effects (symptoms relating to stomach upset, haemolysis, and other symptoms)**<br>• Side effects due to primaquine may happen, but severe ones are rare.<br>• The most common symptoms are:<br>**Gastrointestinal**<br>▪ Stomach pain or back pain<br>▪ Nausea, not wanting to eat<br>▪ Vomiting<br>**Haemolysis**<br>▪ Looking pale<br>▪ Blood in urine causing dark urine<br>▪ Dizziness, breathlessness, or fatigue<br>▪ Jaundice<br>Other symptoms<br>▪ Blue lips/cyanosis<br>• ***Refer to flipchart images of symptoms to watch for***	☐
6	**Advice patients to take primaquine with food to minimize side effects:**<br>• When primaquine is taken on an empty stomach it can cause stomach pains, discomfort, and vomiting.<br>• To minimise the risk of these side effects, patients should be encouraged to take their primaquine tablets with food or during a meal such as breakfast, lunch, or dinner e.g., a portion of rice with a side.<br>• ***Refer to flipchart images of taking tablets with food again***	☐
7	**Explain the difference of mild and severe side effects/adverse effects:**	☐

	• Patients may experience unpleasant symptoms due to malaria, their medication, or another disease. Patients should be encouraged to report these side events to the nurse/doctor.<br>• Mild side effects can be treated with simple changes to their medication (e.g. splitting the dose into a morning dose and an evening dose), (**Drug Administration SOP).**<br>• Severe side effects can disturb the patient's activities and must be treated immediately (See 8. Follow-up).<br>• A summary table and range of side effects table are provided below for a detailed list of symptoms and the range of severity.<br>• ***Refer to flipchart images of mild vs severe symptoms***<br>• ***Show treatment card detailing their G6PD status, treatment and clinic contact details***	
