# Supplementary material for: High-dose, short-course primaquine after point-of-care G6PD testing for the radical cure of Plasmodium vivax malaria: a safety study in Papua New Guinea and Indonesia
Source: Lancet Reg Health West Pac. 2026 Jun 11;71:101903. doi: 10.1016/j.lanwpc.2026.101903 (PMC13276568; doi:10.1016/j.lanwpc.2026.101903)
Supplement: Supplementary File 2 [file mmc2.pdf]

**PROJECT TITLE:**

**SCOPE**

**Short COurse PrimaquinE for the radical cure of *P. vivax***

## INFORMED CONSENT FORM for PARTICIPANTS

**who are invited to contribute in the research titled**

**Short course Primaquine for the radical cure of P. vivax (Stage 1)**

Investigator: Dr. Rini Poespoprodjo, Prof. Inge Sutanto, Dr. Ayodhia Pasaribu

Organization: Universitas Gadjah Mada, Yogyakarta, Indonesia, Universitas Indonesia, Depok, Indonesia, Universitas Sumatra Utara, Medan, Indonesia

Sponsor: Menzies School of Health Research, Australia

**This Informed Consent Form has two parts:**

- **Information Leaflet (to share information about the study with you)**
- **Certificate of Consent (for signatures if you choose to participate)**

**You will be given a copy of the full Informed Consent Form (information sheet and Certificate of Consent)**

**INFORMATION LEAFLET**

**"THIS IS FOR YOU TO KEEP"**

**You can say no**

**For parents or guardians of minors read "you" as "your child"**

## Introduction

The University of Gadjah Mada, University of Indonesia, University Sumatra Utara, and Menzies School of Health Research in Australia are doing a research study to identify better ways to treat people with vivax malaria in your country. The National Department of Health of Indonesia support this study.

We invite you to participate in this study. Before you decide whether to take part, it is important for you to understand why the study is being done and what it will involve.

Please take the time to read the following information carefully. There may be some words that you do not understand. Ask us if there is anything that is not clear and if you would like more information.

## Why is this study done?

You have vivax malaria. Malaria can be a serious disease if it is not treated quickly with good medication . Vivax malaria can make you ill more than once – primaquine is the only drug available in your country that prevents this from happening. It Is given in addition to other drugs to treat malaria. At the moment, people with vivax malaria are given 14 days of primaquine. Sometimes people do not take all  the tablets and the malaria is not cured and comes back after some time.

We think that if people take more primaquine tablets every day but only for 7 instead of 14 days, they are more likely to get all the treatment they need, and therefore the malaria is less likely to come back.

Primaquine can be harmful to patients who have low levels of an enzyme called G6PD. People who do not have sufficiently high G6PD levels can fall very sick from primaquine The biggest risk is severe anaemia, an important drop of proteins carrying the oxygen in your blood, that can make you unwell and even result in you needing to go to hospital.

People with low G6PD levels can be healthy and do not differ from everyone else. It is difficult to identify these people, but there is a new test that can measure G6PD levels. Currently people with vivax malaria are not tested for G6PD levels, and everybody receives the same primaquine treatment regimen.

In this study we will measure G6PD levels in everybody before we give primaquine. People who have sufficiently high G6PD levels will be given a higher dose than normal for 7 days. Those who have lower G6PD levels will receive the same, high dose over 14 days, and those who have very low G6PD levels will receive treatment over 8 weeks. This is to protect them from dangerous side effects.

G6PD testing and the three higher primaquine dose regimens are not currently part of routine practice in your country..

**What kind of study is this?**

In this study we want to know if primaquine can be given over 7 days at a high dose to patients who have normal G6PD levels. We want to know if people fall sick from the new primaquine dosing and if people who receive the new treatment have less vivax malaria than those who receive the standard treatment (0.25mg/kg per day for 14 days).

**Why are you asking me?**

You have vivax malaria and need to receive treatment, including primaquine. We would like to invite you take part in this study.

You can take part in this study if:

- You agree to be part of the study
- You are fine with us visiting you at your house in 3 days and then again in 7 days

You cannot be part of the study if:

- You are pregnant or breast feeding
- You are less than 6 months of age >
- You are severely ill as decided by your doctor
- You have had severe side effects from primaquine before
- Your haemoglobin level (a measure of how much of the protein carrying the oxygen in your blood) is very low

**Do I have to participate?**

No, you don't have to participate if you don't want to. If you choose not to participate, all the services you receive will be as always at the clinic. Your G6PD levels will not be tested and you will receive treatment as is standard practice and we will not collect any information from you.

If you join the study and at any time in the future change your mind and decide that you don't want to participate anymore, please tell us. You will not have to pay a penalty and nobody will be upset with you. You don't have to tell us why you have changed your mind. We will not collect any further information from you, but will keep the information we have already collected.

**What happens if I agree to participate?**

We will ask you to sign a form saying that you agree to participate. You can find the form at the end of this document.

If you agree to join this study, we will ask you some questions about your health and you will be examined.

We will then measure your G6PD and haemoglobin level. When we have your test results we will decide if you can participate and how many tablets you should take and for how long

Someone from the team will come to your house in 3 and in 7 days to make sure you are ok. It is very important that we can contact you on these days.

**What procedures will you be doing on me if I participate?**

We will prick you in the finger and collect a drop of blood from you to measure your G6PD and haemoglobin level. Depending on your G6PD level we will then give you primaquine tablets for:

- 7 days at 1 mg/kg per day (if your G6PD level is normal)
- 14 days at 0.5 mg/kg per day (if your G6PD level is low)
- 8 weeks at 0.75 mg/kg per week (if your G6PD level is very low)

We will explain exactly how and when you should take how many tablets. Someone form the study team will visit you 3 and 7 days after you started taking primaquine. The team member will ask you how you feel, what colour your urine is (dark urine is a sign of severe side effects), and whether you have taken your tablets. If they think you may be developing side effects from primaquine, they will take you back to the clinic and ask you to stop taking your primaquine tablets until we know exactly how you are doing. If they think you are fine, they will remind you to continue taking your primaquine tablets as advised when you were enrolled. They will also ask you on one of these visits what you think about taking primaquine.

Information about your malaria episode, the G6PD and haemoglobin test results, your treatment and any side effect you experience will be collected. If you do not wish to participate in the study your data will only be collected in the routine health facility forms.

**How long will I be in the study?**

You will be in the study as long as you take primaquine (7 or 14 days, or 8 weeks). If you have any problems with your treatment, the study team will continue to follow-up with you until you make a full recovery.

**What risks are there if I take part in the study?**

Primaquine can cause side effects. People who have low G6PD levels can be harmed if they take primaquine and can become veryill and may need to go to hospital. The primaquine treatment we offer to you will depend on your G6PD level. We will therefore test your G6PD level before we decide what treatment is best for you. The test works very well but is not perfect. There is a slight chance that the test does not identify a G6PD deficient person, putting them at risk of dangerous side effects, in particular a drop in proteins carrying the oxygen in the blood. That is why we will check up on you 3 and 7 days after enrolment to make sure that you are ok.

In addition, primaquine can cause abdominal pain, diarrhoeaor vomiting in everybody. This happens more often and is more severe for people when they receive a high dose of primaquine over 7 days. Feeling sick happens less often if you take primaquine together with a meal.

When we take blood, you may get a small bruise and mild pain on the finger where the blood is collected. There is also a very small risk of infection; the risk is very small because we always use clean materials.

**What do I get if I participate?**

We cannot give you any money for participating. You will receive treatment that can cure of your malaria infection. It is still possible that you will be infected with malaria again sometime in the future.

The education provided as part of the study will increase your knowledge of malaria and its treatment. Information from this study will enable your government to develop better guidelines on how to treat malaria and this will be beneficial for you and your community.

### What is the Study Cost?

Your participation in the study is free. All additional tests (G6PD and Haemoglobin tests) and treatment are free. If you fall sick or get injured because of this study, emergency medical treatment is available at the nearest provincial hospital and transport to the hospital and hospital fees will be paid by the study team.

### Who will find out if I participate?

We will not share your name or identity with anyone outside the research team. However, the research being done in the health centers may draw attention and if you participate you may be asked questions by other people in the health center or in the community. We will not be sharing identifiable information about you with anyone outside of the research team. A study team member will come and visit you at your place in 3 and 7 days and it is likely that your household members and neighbours will notice the visit and find out that you are participating in the study. Nobody from the team will tell anyone outside of the study team about your test results or anything you said to a study team member.

### Who will see my de-identified study data?

Any information about you will be identified by a number only, but not your name. Only the study team will know what your number is and we will protect that information with a lock and key Nothing will be attributed to you by name.

People working in the following organisation will have access to your anonymised medical information:

- The local study investigators and Ministry of Health.
- The study sponsors
- The Ethics Committees or Investigational Review Boards overseeing this study
- Insurers
- The independent committee overseeing the study conduct
- MMV Medicines for Malaria Venture (who provides technical and financial support)
- Unitaid (who is funding the study)

The knowledge that we get from this research will be made widely available to the public. Information generated from this study will be published and presented, but your identity will not be revealed. Your information will be stored for a minimum period of 5 years after study end in a secure place. The knowledge that we get from doing this study will be shared with you through community meetings.

**Who to contact**

If you have any questions, you can ask them now or later. If you wish to ask questions later, you may contact:

| | |
|---|---|
| Papua | Dr. Rini Poespoprodjo, Ph +62811 90738, Universitas Gadjah Mada, Yogyakarta, Indonesia |
| Lampung | Prof Inge Sutanto, Ph +62 812 961 1034, Universitas Indonesia, Depok, Indonesia |
| North Sumatra | Dr. Ayodhia Pasaribu, Ph +62 812 602 4392, Universitas Sumatra Utara, Medan, Indonesia. |

Questions to elucidate understanding

- *Can you tell me if you have understood correctly why the study is taking place?*
- *Can you tell me if you have understood that you do not have to join the study*
- *Do you have any other questions?*

**Thank you very much for your time. Would you like to join the study?**

| | |
|---|---|
| Papua | Universitas Gadjah Mada Ethics Committee<br>Email: mhrec_fmugm@ugm.ac.id or Ph +62 (274) 588 688 ext. 17225 |
| Lampung | Universitas Indonesia FKUI/RSCM Ethics Committee Email: komite-etik@fk.ui.ac.id or Ph: 021 315 7008, Whatsapp: 0856-8701-608 |
| North Sumatra | Universitas Sumatra Utara Ethics Committee<br>Email: komiteetik@usu.ac.id or Ph. +62 (61) 8211 045, 821 0555 |
| Australia | NT Department of Health and Menzies School of Health Research<br>Email: ethics@menzies.edu.au, Ph. +61 8946 8600 |

**Concerns or Complaints**

The sponsor of this study is committed to repair any damage you may experience within the frame of this study. To that end, an insurance has been contracted, which will cover all medical expenses for all study-related damages.

If you have any concerns or complaint about:

- the way you are invited to take part in this project,
- how the project is explained to you and your involvement,
- any privacy and confidentially issues related to this project, or
- the information you provide to the project and how it is used,

you are invited to contact the Ethics Administration, NT Department of Health and Menzies School of Health Research Department of Health and Menzies School of Health Research on +61 8946 8600 or email ethics@menzies.edu.au

**You can ask me any more questions about any part of the research study if you wish to. Do you have any questions?**

**PROJECT TITLE:**
**SCOPE**

**Short COurse PrimaquinE for the radical cure of *P. vivax***

## CERTIFICATE OF CONSENT

**"This means that you can say NO"**

| This consent if for myself / my child | Myself | My child |
|---|---|---|
| I Know what the study is about and what will happen if I participate. | YES | NO |
| I have asked all questions I had, and all questions I had have been answered. | YES | NO |
| I agree to participate | YES | NO |

**Print Name of Participant:**

_______________________________________________

**Signature of Participant:**

_______________________________________________

**Date:** ____ / ____ / ________

Day/ Month/ Year

***If illiterate*** [1]

I have witnessed the accurate reading of the consent form to the potential participant, and the individual has had the opportunity to ask questions. I confirm that the individual has given consent freely.

**Thumb print of participant** ☐

**Print name of witness:** _______________________________________________

**Signature of witness:** _______________________________________________

**Date:** ____ / ____ / ________

Day/month/year

[1] A literate witness must sign (if possible, this person should be selected by the participant and cannot have a connection to the research team). Participants who are illiterate must include their thumb print as well.

**PROJECT TITLE:**
**SCOPE**

**Short COurse PrimaquinE for the radical cure of *P. vivax***

**Statement by the researcher/person taking consent**

I have accurately read out the information sheet to the potential participant, and to the best of my ability made sure that the participant understands that the following will be done if they agree and fulfill all the criteria:

1. They will be asked some questions about their health.
2. They will then undergo physical and laboratory examinations to measure their G6PD activity and haemoglobin level
3. Based on these results, their treatment and medication dosage will be explained to them in detail
4. One of the research team will come to their house on day 3 and 7 to check how they are feeling and do some follow-up tests.

I confirm that the participant was given an opportunity to ask questions about the study, and all the questions asked by the participant have been answered correctly and to the best of my ability. I confirm that the individual has not been coerced into giving consent, and the consent has been given freely and voluntarily.

**A copy of this Information Consent Form has been provided to the participant.**

**Print Name of Researcher/person taking the consent:**________________________________

**Signature of Researcher /person taking the consent:** ________________________________

**Date:** ____ **/** ____ **/** ________

Day/ Month/ Year

**PROJECT TITLE:**

**SCOPE**

**Short COurse PrimaquinE for the radical cure of *P. vivax***

## ASSENT FORM for PARTICIPANTS

**who are invited to contribute in the research titled**

**Short course Primaquine for the radical cure of *P. vivax***

| | |
|---|---|
| Investigator: | Dr. Rini Poespoprodjo, Prof. Inge Sutanto, Dr. Ayodhia Pasaribu |
| Organization: | Universitas Gadjah Mada, Yogyakarta, Indonesia, Universitas Indonesia, Depok, Indonesia, Universitas Sumatra Utara, Medan, Indonesia |
| Sponsor: | Menzies School of Health Research, Australia |

**This Assent Form has two parts:**

- **Information Leaflet (to share information about the study with you)**
- **Certificate of Consent (for signatures if you choose to participate)**

**You will be given a copy of the full Informed Consent Form (information sheet and Certificate of Consent)**

**INFORMATION LEAFLET for ASSENT - Stage 1**

**"THIS IS FOR YOU TO KEEP"**

**<u>You can say no</u>**

You have vivax malaria. Malaria can be a severe disease if not treated immediately and with good drugs. Don't worry, you will receive good treatment in a couple of minutes. You have a form of malaria that is tricky to treat (vivax malaria). While most of the malaria bugs in your body are in your blood and make you feel sick, some are hiding and will make you sick again in a couple of weeks if they are not treated. Accordingly, you need two types of drugs. One that makes you feel better immediately and another one called primaquine that removes the hidden malaria bugs from your body. Primaquine is usually taken over 14 days, but many people forget to take all tablets. If people don't take all tablets, malaria may come back because not all hidden malaria bugs have been treated. We think that taking more tablets over 7 days instead might be easier and people are less likely to forget to take them. The current practice is 14 days of treatment. Taking the tablets over 7 days means a higher daily dose and there is an increased risk that you become ill because of the drug.

We want to try this out together with you. If you agree to be part of this study, we will first test that you can take the tablets without having dangerous side effects. The test requires a couple of drops of blood which we will collect from a finger prick. The finger prick is going to hurt a bit. A doctor will then examine you and we will ask you and your parents some questions. We will then give you all the tablets you need to take and explain to you and your parents how to take them.

In some people, the drug can make you ill and you may require going to hospital. The test will tell us if this might happen. If this is the case, we will treat you with fewer tablets per day but over 14 days. We will explain exactly to you and your parents how to take the tablets so that you won't get sick. The test is not perfect. To make sure that you are ok, we will visit you in 3 days and again in 7 days and each time we will prick you in the finger for another test.

After taking the tablets some people feel sick, their stomach hurts, they have diarrhoea and vomit. This occurs more frequently if you take a higher dose. It is important you take the tablets with food to avoid being sick.

**If you don't want to be part of this study, nobody will be angry at you. You will not be punished, and you will receive the usual good treatment, over 14 days and will be treated as friendly as everybody else who comes to this clinic.** You do not have to tell us why you don't want to participate, and you can always change your mind later without anyone being upset. If you agree to participate, we will not tell anyone that you are participating.

If you have any questions, you can ask them now or later. If you wish to ask questions later, you can contact:

| | |
|---|---|
| Papua | Dr. Rini Poespoprodjo, Ph +62811 90738, Universitas Gadjah Mada, Yogyakarta, Indonesia |
| Lampung | Prof. Inge Sutanto, Ph +62 812 961 1034, Universitas Indonesia, Depok, Indonesia |
| North Sumatra | Dr. Ayodhia Pasaribu, Ph +62 812 602 4392, Universitas Sumatra Utara, Medan, Indonesia. |

**Thank you very much for your time. Would you like to join the study?**

**INFORMATION LEAFLET for ASSENT - Stage 2**

**"THIS IS FOR YOU TO KEEP"**

**<u>You can say no</u>**

You have vivax malaria. Malaria can be a severe disease if not treated immediately and with good drugs. Don't worry, you will receive good treatment in a couple of minutes. You have a form of malaria that is tricky to treat (vivax malaria). While most of the malaria bugs in your body are in your blood and make you feel sick, some are hiding and will make you sick again in a couple of weeks if they are not treated. Accordingly, you need two types of drugs. One that makes you feel better immediately and another one called primaquine that removes the hidden malaria bugs from your body. Primaquine is usually taken over 14 days, but many people forget to take all tablets. If people don't take all tablets, malaria may come back because not all hidden malaria bugs have been treated. We think that taking more tablets over 7 days instead might be easier and people are less likely to forget to take them. The current practice is 14 days of treatment. Taking the tablets over 7 days means a higher daily dose and there is an increased risk that you become ill because of the drug.

We want to try this out together with you. If you agree to be part of this study, we will first test that you can take the tablets without having dangerous side effects. The test requires a couple of drops of blood which we will collect from a finger prick. The finger prick is going to hurt a bit. A doctor will then examine you and we will ask you and your parents some questions. We will then give you all the tablets you need to take and explain to you and your parents how to take them.

In some people, the drug can make you ill and you may require going to hospital. The test will tell us if this might happen. If this is the case, we will treat you with fewer tablets per day but over 14 days. We will explain exactly to you and your parents how to take the tablets so that you won't get sick. The test is not perfect. To make sure that you really are ok, we will visit you in 3 days.

After taking the tablets some people feel sick, their stomach hurts, they have diarrhoea and vomit. This occurs more frequently if you take a higher dose. It is important you take the tablets with food to avoid being sick.

**If you don't want to be part of this study, nobody will be angry at you. You will not be punished, and you will receive the usual good treatment, over 14 days and will be treated as friendly as everybody else who comes to this clinic.** You do not have to tell us why you don't want to participate, and you can always change your mind later without anyone being upset. If you agree to participate, we will not tell anyone that you are participating. If you

have any questions, you can ask them now or later. If you wish to ask questions later, you can contact:

| | |
|---|---|
| Papua | Dr. Rini Poespoprodjo, Ph +62811 90738, Universitas Gadjah Mada, Yogyakarta, Indonesia |
| Lampung | Prof. Inge Sutanto, Ph +62 812 961 1034, Universitas Indonesia, Depok, Indonesia |
| North Sumatra | Dr. Ayodhia Pasaribu, Ph +62 812 602 4392, Universitas Sumatra Utara, Medan, Indonesia. |

**Thank you very much for your time. Would you like to join the study?**

**PROJECT TITLE:**
**SCOPE**

**Short COurse PrimaquinE for the radical cure of *P. vivax***

**CERTIFICATE OF ASSENT stages 1-2**
**needs to be accompanied by a consent form from a legal guardian**

**"This means that you can say NO"**

| | | |
|---|---|---|
| I know what the study is about and what will happen if I participate. I realize that I will be pricked in the finger at least 3x / 1x*. I realize that the treatment may make me sick. | YES | NO |
| I have asked all questions I had, and all questions have been answered | YES | NO |
| I agree to participate | YES | NO |

***Number of times depends on study stage**

**Print Name of Participant:**

______________________________________________

**Signature of Participant:**

______________________________________________

**Date:** ____ **/** ____ **/** ________

Day/ Month/ Year
