# Supplementary material for: High-dose, short-course primaquine after point-of-care G6PD testing for the radical cure of Plasmodium vivax malaria: a safety study in Papua New Guinea and Indonesia
Source: Lancet Reg Health West Pac. 2026 Jun 11;71:101903. doi: 10.1016/j.lanwpc.2026.101903 (PMC13276568; doi:10.1016/j.lanwpc.2026.101903)
Supplement: Supplementary File 3 [file mmc3.pdf]

Staff ID Number: _____________ Study Site: ________________

Level 3 Review Form - MEDICAL EVALUATION

To be completed for all patients referred after Level 2 review with suspected SAE / AESI

Date/Time of Level 3 Review: ___ / ___ / 202__ Time: ____:____ (24hr)

Location: ☐ Clinic ☐ Hospital ☐ Other: ____________________

Report Type: ☐ Initial ☐ Follow-up FU #: ____

PATIENT DETAILS

**SCOPE Study ID:** ____________	**Malaria Card #:** ____________	**Medical Record #:** ____________
**Patient Initials:**	**Sex:** ☐ M ☐ F	**Age:** ____ yrs ____ mths

Initial Treatment: ☐ PQ7 ☐ PQ14 ☐ PQ8W ☐ Low dose PQ14

CLINICAL PRESENTATION

**Date Referred:**	___ / ___ / 202__	**Temperature:** ____.___ °C
**Pulse:** _____ per min	**Resp rate:** _____ per min	
**Symptom**	**Severity (see criteria table)**	**If present, date of onset**
**Abdominal pain**	None 1 2 3 4	___ / ___ / 202__
**Nausea**	None 1 2 3 4	___ / ___ / 202__
**Unable to eat**	None 1 2 3 4	___ / ___ / 202__
**Vomiting**	None 1 2 3 4	___ / ___ / 202__
**Back pain**	None 1 2 3 4	___ / ___ / 202__
**Breathlessness**	None 1 2 3 4	___ / ___ / 202__
**Dizziness**	None 1 2 3 4	___ / ___ / 202__
**Fatigue**	None 1 2 3 4	___ / ___ / 202__
**Fever**	Yes / No	___ / ___ / 202__
**Severe pallor**	Yes / No	___ / ___ / 202__
**Jaundice**	Yes / No	___ / ___ / 202__
**Cyanosis (Blue lips)**	Yes / No	___ / ___ / 202__
**Dark (red or black) urine (Hillmen 7-10)**	Yes / No<br>Colour #:_____	___ / ___ / 202__
**Other:**	________________________	___ / ___ / 202__

NARRATIVE:

____

____

____

____

____

____

Staff ID Number: __________ Study Site: _____________ SCOPE Study ID: _____________

INVESTIGATIONS			
**Haemoglobin**	**Date**	**Result**	**Method**
Baseline (A)	___ /___ / 202__	Hb: ___ . __ g/dL	☐ Biosensor
Today (B)	___ /___ / 202__	Hb: ___ . __ g/dL	☐ Biosensor ☐ Hemocue ☐ CBC
Nadir (C)	___ /___ / 202__	Hb: ___ . __ g/dL	☐ Biosensor ☐ Hemocue ☐ CBC
**Max Fall Hb:** ____ . __ g/dL (C-A)		**Max % Fall Hb:** ____.__ %	
**G6PD:** Baseline	___ /___ / 202__	___. ___ U/g Hb	
	___ /___ / 202__	___. ___ U/g Hb	
	___ /___ / 202__	___. ___ U/g Hb	
**Met Hb:**	___ /___ / 202__	____. ___ %	Measured: ☐ Yes ☐ No
**Sp0₂:**	___ /___ / 202__	____ %	

ADVERSE EVENT CLASSIFICATION	
**Severity**	Max Graded Symptom: **Date of onset**: ___ /___ / 202__<br>☐ None ☐ Grade 1 ☐ Grade 2 ☐ Grade 3 ☐ Grade 4 ☐ Grade 5
**For Follow Up Visits**	Complete for any previously flagged symptoms: ☐ Ongoing<br>☐ Recovered/resolved ☐ Recovering/resolving ☐ Worsening
**AESI - Haemolysis**<br>☐ Yes ☐ No	Any of the following: **Date of onset**: ___ /___ / 202__<br>☐ Grade 3 or 4: Fatigue, Dizziness, Breathlessness (onset after starting PQ)<br>☐ Severe Pallor or Jaundice<br>☐ Dark Urine: Hillmen >7<br>☐ Fall in Hb > 3 g/dL<br>☐ Hb < 7g/dl
**AESI – Gastrointestinal**<br>☐ Yes ☐ No	**Date of onset**: ___ /___ / 202__<br>☐ Grade 3 / 4: Abdominal Pain, Nausea, Anorexia or Vomiting
**AESI - MetHb**<br>☐ Yes ☐ No	☐ MetHb: >10% **Date of onset**: ___ /___ / 202__<br>**Plus**: Grade 3 or 4: Breathlessness or Dizziness
**SAE**<br>☐ Yes ☐ No	☐ Death<br>☐ Life threatening<br>☐ Hospitalisation or prolongation of hospitalisation<br>☐ Persistent or significant disability<br>☐ Congenital abnormality/birth defect

	☐ Other__________________________

CLINICAL MANAGEMENT

**Changes to PQ**	☐ Continued ☐ Withheld ☐ Finished ☐ Ceased ☐ Restarted ☐ Modified
	Dose: ___.__ mg ☐ 1x Day ☐ 2x Day ☐ 1x Week.<br>Duration of treatment remaining (including today): ___ ☐ Days ☐ Wks

NARRATIVE for AESI: If meets criteria for SAE, complete SAE form ☐

____

____

____

____

____

____

____

____

**Planned Review**	☐ No further follow required<br>☐ Review again on Day ____ Date: __ / __ / 202__<br>☐ Referred to Hospital: ☐ Yes ☐ No<br>If Referred: Date: __ / __ / 202__ Time: ___ : ___

NOTIFICATION ☐ Yes ☐ No

Any confirmed AESI must be notified to the PIs and GMM as soon as possible

If an SAE, complete an SAE form and send report to PIs, GMM and MMV within 24 hrs

**Time of Notification**	Date: __ / __ / 202___ Time: ___ : ___
**Completion of SAE Form**	☐ Yes ☐ Not Applicable
**Process**	☐ WhatsApp<br>☐ Email scanned Level 3 Form +/- SAE Form
**People Notified**	☐ Local PI<br>☐ Study MD<br>☐ Menzies / Burnet PI<br>☐ Global Medical Monitor<br>☐ MMV Chief Medical Officer

Staff ID Number: __________ Study Site: _____________ SCOPE Study ID: _____________

Term	Grade 1	Grade 2	Grade 3	Grade 4
Abdominal pain	Mild pain	Moderate pain; limiting instrumental ADL	Severe pain; limiting self-care ADL	
Nausea	Loss of appetite without alteration in eating habits	Oral intake decreased without significant weight loss, dehydration or malnutrition	Inadequate oral caloric or fluid intake; tube feeding, or hospitalisation indicated	
Anorexia	Loss of appetite without alteration in eating habits	Oral intake altered without significant weight loss or malnutrition; oral nutritional supplements indicated	Associated with significant weight loss or malnutrition	Life-threatening consequences; urgent intervention indicated
Vomiting	Intervention not indicated	Outpatient IV hydration; medical intervention indicated	Tube feeding, or hospitalisation indicated	Life-threatening consequences
Back Pain	Mild pain	Moderate pain; limiting instrumental ADL	Severe pain; limiting self-care ADL	
Breathlessness	Shortness of breath with moderate exertion	Shortness of breath with minimal exertion; limiting instrumental ADL	Shortness of breath at rest; limiting self-care ADL	Life-threatening consequences; urgent intervention indicated
Dizziness	Mild unsteadiness or sensation of movement	Moderate unsteadiness or sensation of movement; limiting instrumental ADL	Severe unsteadiness or sensation of movement; limiting self-care ADL	
Fatigue	Fatigue relieved by rest	Fatigue not relieved by rest; limiting instrumental ADLs	Fatigue not relieved by rest; limiting self care ADLs	

HILLMEN URINE CHART

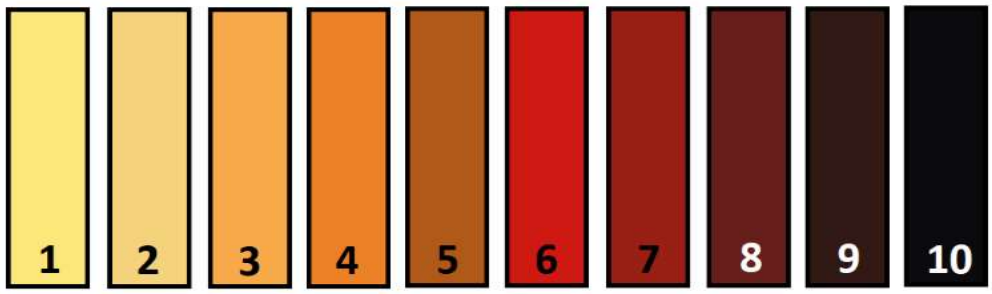

Staff ID Number: ______________ Study Site: _________________

SERIOUS ADVERSE EVENT FORM – MD

**Date of Assessment:** __ / __ / 202__	**Time:** ___ : ___ (24hr)

Report type: □ Initial □ Follow-up FU #: ____

Location of Assessment (Hospital/Clinic name):

PATIENT DETAILS

SCOPE ID: ________ **Patient Initials**: _________ **Medical Record Number:** ________________

Sex: □ M □ F **Age:** ________yrs ______ mths **Date of Birth:** __ /__ /202___

Weight: ________ . __ kg

Country: _______________________

MALARIA DETAILS - Only complete for the initial SAE report

Date of Malaria Diagnosis: __ / __ / 202__ **Method:** □ Microscopy □ RDT □ Other___

Malaria Species: □ Pf □ Pv □ Pm □ Po □ Pv/PAN □ Unknown

Baseline Parasitaemia: ________________ per uL □++++ □+++ □ ++ □+ □ Unknown

TREATMENT DETAILS - Only complete for the initial SAE report

Schizontocidal Treatment: □ CQ □ AL □ DP □ Quinine □ Other: ____________

Date PQ Commenced: ___ /___ /202____

Daily Dose of PQ: ________.__ mg **Calculated mg/kg Dose:** ___________

PQ Dosing schedule: □ 1x day □ 2x day □ 1x week □ No Tablets taken

Planned duration of PQ: □ 7 days □ 14 days □ 8 Weeks……..□ 8 Weeks

Number of PQ doses taken before adverse event detected: _________

Did the patient take their last PQ tablets with food? □ Yes □ No □ Unsure

CONCOMITANT MEDICATION □ Yes □ No

**Medication**	**Start Date** dd/mm/yyyy	**Stop Date** dd/mm/yyyy	**Dose**	**Frequency**
	__ / __ / 202__	__ / __ / 202__		
	__ / __ / 202__	__ / __ / 202__		
	__ / __ / 202__	__ / __ / 202__		
	__ / __ / 202__	__ / __ / 202__		
	__ / __ / 202__	__ / __ / 202__		
	__ / __ / 202__	__ / __ / 202__		

Staff ID Number: ___________ Study Site: ______________ SCOPE Study ID: ______________

CLINICAL PRESENTATION, TIMING AND PROGRESS

ADVERSE EVENT (AE): Main Diagnosis:____________________________________

Date AE Started:	__ / __ / 202__	
Date AE Met Serious Criteria:	__ / __ / 202__	
Date SAE Detected:	__ / __ / 202__	
Date of Hospitalisation:	__ / __ / 202__	☐ Not applicable
Date of Discharge:	__ / __ / 202__	☐ Still in hospital

NARRATIVE

____

____

____

____

____

____

____

____

RELEVANT MEDICAL HISTORY ☐ Yes ☐ No

Only complete for the initial SAE report

**Medical condition**	Start Date dd/mmm/yyyy	Stop Date dd/mmm/yyyy	Ongoing
	__ / __ / 202__	__ / __ / 202__	☐
	__ / __ / 202__	__ / __ / 202__	☐
	__ / __ / 202__	__ / __ / 202__	☐
	__ / __ / 202__	__ / __ / 202__	☐

INVESTIGATIONS

**Met Hb:**	___ /___ / 202_	____. ___ %	Measured: ☐ Yes ☐ No
**SpO_2:**	___ /___ / 202_	____. ___ %	
**Haemoglobin**			
Baseline (A)	__ / __ / 202__	Hb: __ . __ g/dL	
At time of Event Detection(B)	__ / __ / 202__	Hb: __ . __ g/dL	☐ Biosensor ☐ Hemocue ☐ CBC
Current (C)	__ / __ / 202__	Hb: __ . __ g/dL	☐ Biosensor ☐ Hemocue ☐ CBC
Nadir (D)	__ / __ / 202__	Hb: __ . __ g/dL	☐ Biosensor ☐ Hemocue ☐ CBC
		**Max Fall in Hb:** (D-A)	Hb: __ . __ g/dL

**Maximum % Fall in Hb:** 100*(D-A)/A			____.__ %
Complete for repeated investigations			
**Met Hb:**	___/___/ 202__	____. ___ %	
	___/___/ 202__	____. ___ %	
	___/___/ 202__	____. ___ %	
	___/___/ 202__	____. ___ %	
**Haemoglobin**	___/___/ 202__	Hb: ____. ___ g/dL	☐ Biosensor ☐ Hemocue ☐ CBC
**Check for nadir (D)**	___/___/ 202__	Hb: ____. ___ g/dL	☐ Biosensor ☐ Hemocue ☐ CBC
	___/___/ 202__	Hb: ____. ___ g/dL	☐ Biosensor ☐ Hemocue ☐ CBC
	___/___/ 202__	Hb: ____. ___ g/dL	☐ Biosensor ☐ Hemocue ☐ CBC

OTHER RELEVENT INVESTIGATIONS

G6PD STATUS - Only complete for the initial SAE report

Date of Testing: __ / __ / 202__

Quantitative Result: ____ . __ U/g Hb ☐ Deficient ☐ Intermediate ☐ Normal

Genotyping: ☐ Not done ☐ Normal ☐ Variant ______________

LABORATORY TESTS – If follow up report just add relevant updates

**Test**	**Date**	**Result**	**Other:**
**WBC:**	___/ ___/ 202__	_________ x10^9	
**Plt:**	___/ ___/ 202__	_________ x10^9	
**Na:**	___/ ___/ 202__	______ µmol/L	
**K:**	___/ ___/ 202__	______ µmol/L	
**Urea:**	___/ ___/ 202__	______ µmol/L	
**Total Bili:**	___/ ___/ 202__	______ µmol/L	
**Unconj Bili:**	___/ ___/ 202__	______ µmol/L	
**ALP**	___/ ___/ 202__	______ µmol/L	
**ALT**	___/ ___/ 202__	______ µmol/L	
**LDH:**	___/ ___/ 202__	______ µmol/L	

CLASSIFICATION

**SAE**<br>The reason why classified as Serious	☐ Death:<br>☐ Life threatening:<br>☐ Hospitalisation or prolongation of hospitalisation:<br>☐ Persistent or significant disability<br>☐ Is a congenital abnormality / birth defect

SERIOUS ADVERSE EVENT (SAE) – MD

Staff ID Number: ___________ Study Site: ______________ SCOPE Study ID: ______________

	☐ Is an important and significant medical event
**Organ Systems Involved**	☐ Haematological ☐ Gastrointestinal ☐ Respiratory<br>☐ Cardiological ☐ Renal ☐ Liver ☐ Neurological
**Relationship (Causality) to PQ**	☐ Not related ☐ Unlikely related ☐ Possibly related<br>☐ Probably related ☐ Definitely related

**CLINICAL MANAGEMENT**		
**IV Fluids**	☐ Yes ☐ No	
**Blood transfusion**	☐ Yes ☐ No	Number of units: ______ Date: __/__/202_
**Dialysis**	☐ Yes ☐ No	☐ Peritoneal dialysis ☐ Hemodialysis
**Changes to PQ**	☐ Continued ☐ Withheld ☐ Finished<br>☐ Ceased ☐ Restarted ☐ Modified	
	If Restarted: Date Restarted: __/__/202_	
	Dose: ___.__ mg ☐ 1x day ☐ 2x day<br>Duration of treatment remaining (including today): _______ ☐ Days ☐ Wks	

NARRATIVE:

____

____

____

____

____

____

____

____

____

____

____

____

____

____

____

____

Staff ID Number: __________ Study Site: _____________ SCOPE Study ID: _____________

OUTCOME	
☐ Recovered / Resolved	
☐ Recovering / Resolving	
☐ Not recovered / Not resolved	
☐ Recovered / Resolved with sequelae Specify:	
☐ Fatal: Date of death: ___ / ___ / 202__<br>Cause of death:	
☐ Unknown	
☐ AE Ongoing	
AE Stopped (Last date AE was present) ___ / ___ / 202__	
**NOTIFICATION ☐ Yes ☐ No**<br>Inform PIs, GMM and MMV within 24 hrs	
**Time of Notification**	Date: __ / __ / 202__ Time: ___ : ___
**Process**	☐ WhatsApp<br>☐ Email scanned Level 3 Form +/- SAE Form
**People Notified**	☐ Local PI<br>☐ Study MD<br>☐ Menzies / Burnet PI<br>☐ Global Medical Monitor<br>☐ MMV Chief Medical Officer

CLINICIAN RESPONSIBLE FOR THE REVIEW
**Name :** Date: __ / __ / 202__
**Role :**
**Email** :
**Signature:**
