# Supplementary material for: High-dose, short-course primaquine after point-of-care G6PD testing for the radical cure of Plasmodium vivax malaria: a safety study in Papua New Guinea and Indonesia
Source: Lancet Reg Health West Pac. 2026 Jun 11;71:101903. doi: 10.1016/j.lanwpc.2026.101903 (PMC13276568; doi:10.1016/j.lanwpc.2026.101903)
Supplement: Supplementary File 4 [file mmc4.pdf]

SCOPE: Statistical Analysis Plan

Protocol title: Feasibility of high daily dose short course primaquine after G6PD testing for the radical cure of *Plasmodium vivax* malaria

Short title: **S**hort **CO**urse **P**rimaquin**E** for the radical cure of P. vivax (SCOPE)

Version: 9.0

Authors: Professor Julie A Simpson

Assoc/Professor Amalia Karahalios

Dr Alistair McLean

Centre for Epidemiology and Biostatistics, and Methods & Implementation Support for Clinical and Health (MISCH) research Hub
University of Melbourne, Melbourne, Australia

Dr Nicholas Douglas

Menzies School of Health Research, Darwin, Australia

Date: 11/11/2025

This document has been written based on information contained in the SCOPE_PQFS_Master Protocol (version 4.0, 28/04/2023, Protocol Identifying Number - MMV_PQ_MASTER_21_01).

SAP Revision History

Version	Reason(s) for change	Date
1.0	Original	21-JUL-2023
2.0	Specified pre-defined analyses, reconfigured dummy tables, added data dictionary.	14-NOV-2023
3.0	Added *post hoc* analyses of comparative performance of HemoCue and SD Biosensor haemoglobin measurements following anecdotal evidence of some seemingly erratic SD Biosensor haemoglobin results	7-FEB-2024
4.0	Added table for *post hoc* analyses of comparative performance of HemoCue and SD Biosensor haemoglobin measurements in Appendix 2 and 3	27-FEB-2024
5.0	Updated Stage 2 power and precision calculations given change in predicted patient recruitment in Stage 2	21-JUN-2024
6.0	Updated Stage 2 power calculations for detection of a reduction in incidence due to an error found in initial calculation. Also provide power calculations for detection of individual-level reduction in risk of recurrence in Stage 2.<br><br>Two additional analyses added to assess the association between haemoglobin and G6PD activity, and the additional exploratory endpoint, methaemoglobin levels on days 3 and 7.	23-OCT-2024
7.0	Added dummy Table S1a for protocol violations in Stage 2 in appendix 3	10-FEB-2025
8.0	Added to Section11:<br><br>i) exploratory analyses for a new Stage 2 effectiveness endpoint (S2.SE18) - cumulative risk of representation to the same health facility with symptomatic *P. vivax* malaria within 6 months of patients enrolled and not enrolled in SCOPE in post-implementation stages.	07-JUL-2025
9.0	-deletion of exploratory analysis for haemoglobin change (S2.EE1 absolute & S2.EE2 proportional in the first 3 days) as haemoglobin data only routinely collected at baseline in Stage 2.<br><br>-addition of subgroup of Sumatra compared to PNG & Indonesian Papua.<br><br>-sensitivity analysis of S2.SE15 changed from pre-implementation period of 3 years (data not available across all clinics) to including only incidence data from previous 12 months.	11-NOV-2025

	-sensitivity analysis added which includes the stage 1 data in the post-implementation period for S2.SE15.<br><br>-S2.SE15 – analysis models for both level and slope change previously had indicator variables marking the pre- and post-implementation periods differently. These have been made uniform, so that estimates are based on the same set of assumptions.<br><br>-addition of sensitivity analysis for S2.SE18 for Indonesian sites, including only patients with a malaria card number.<br><br>-addition of clarifying detail for S2.SE18; propensity scores only to be used when an imbalance in baseline characteristics exists, deletion of subgroup analysis for patients <30% G6PD activity, addition of stage 1 data in SCOPE-enrolled group.	

SAP Signatures

I give my approval for the attached SAP entitled SCOPE_SAP_v9.0_2025-07-10 dated 11-11-2025:

On behalf of the Principal Investigators

Name	Signature	Date
Prof Ric N Price<br>(Principal investigator - Indonesia)		24/11/25
Prof Leanne Robinson<br>(Principal investigator – PNG)		24/11/25

SAP Authors

Name	Signature	Date
Prof Julie Simpson<br>(Senior statistician)		24/11/25
Dr Nicholas Douglas<br>(Senior Epidemiologist)		24/11/25
A/Prof Amalia Karahalios<br>(Senior statistician)		24/11/25

Dr Alistair McLean<br>(Statistician)		

Table of Contents

1 INTRODUCTION 9
1.1 Preface 9
1.2 Purpose of the analyses 9
2 Study Objectives and Endpoints 10
2.1 Study objectives 10
2.1.1. Stage 1 – Preliminary safety and feasibility study 10
2.1.2. Stage 2 – Large-scale implementation and feasibility study 11
2.2 Endpoints 12
2.2.1. Stage 1 – Preliminary safety and feasibility study 12
2.2.2. Stage 2 – Large-scale implementation and feasibility study 13
3 Study Methods 15
3.1 Study design 15
3.1.1. Stage 1 – Preliminary safety and feasibility study 16
3.1.2. Stage 2 – Large-scale implementation and feasibility study 17
3.2 Inclusion and exclusion criteria 18
4 Sample size 19
4.1 Stage 1 19
4.2 Stage 2 19
4.2.1 Revised and corrected Stage 2 power and precision calculations 19
4.2.2 Original power and precision calculations – now superseded 22
5 Analysis Objectives 23
5.1. Stage 1 – Preliminary safety and feasibility study 23
5.2. Stage 2 – Large-scale implementation and feasibility study 23
6 Analysis sets/Subgroups 24
6.1. Stage 1 – Preliminary safety and feasibility study 24
6.2. Stage 2 – Large-scale implementation and feasibility study 24
7 Outcomes and Covariates 25
8 Handling of Missing Values 25
9 Statistical Methodology 25
9.1. Stage 1 – Preliminary safety and feasibility study 26
9.2. Stage 2 – Large-scale implementation and feasibility study 26
9.3 Measures to Adjust for Confounders, Heterogeneity and Autocorrelation 28
10 Sensitivity Analyses 29

11 Exploratory analyses comparing patients enrolled and not enrolled in SCOPE 29

12 Quality Control Plans 31

12 References 32

Appendix 1 33

Appendix 2 39

Appendix 3 – Dummy tables and figures 40

Appendix 4 - Implementation definitions...55

LIST OF ACRONYMS

Acronym	Definition
ACT	Artemisinin-based Combination Therapy
ADL	Activity of Daily Living
AE	Adverse Event
AESI	Adverse Event of Special Interest
AMM	Adjusted Male Median
ANZCTR	Australian New Zealand Clinical Trials Registry
BRIN	*Badan Riset dan Inovasi Nasional* (Indonesia)
CHW	Community Health Worker
CI	Confidence Interval
CQI	Continuous Quality Improvement
DFAT	Department of Foreign Affairs and Trade (Australia)
FDA	Food and Drug Administration
FGD	Focus Group Discussion
G6PD	Glucose-6-Phosphate Dehydrogenase
G6PDd	G6PD deficiency
GI	Gastrointestinal Intolerance
GST	Global Study Team
HCP	Healthcare Provider
MoH	Ministry of Health
NDA	National Drug Authority
NDoH	National Department of Health
NMCP	National Malaria Control Program
PCR	Polymerase Chain Reaction
PNG	Papua New Guinea
PQ	Primaquine
PQ7	Primaquine 1.0 mg/kg/day for 7 days
PQ14	Primaquine 0.5 mg/kg/day for 14 days
PQ8W	Primaquine 0.75mg/kg/week for 8 weeks
RDT	Rapid Diagnostic Test
SAE	Serious Adverse Event
SCOPE	**S**hort **CO**urse **P**rimaquin**E** for the radical cure of *P. vivax*
SMC	Safety Monitoring Committee
SRT	Safety Review Team

YPKMP	*Yayasan Pengembangan Kesehatan dan Masyarakat Papua* (Papua Health and Community Development Foundation)

1 INTRODUCTION

1.1 Preface

Outside of subsaharan Africa, *Plasmodium vivax* is becoming the predominant cause of malaria. Although prevention of relapses is crucial for reducing transmission of this important disease, only two drugs (primaquine and tafenoquine) are licensed for treatment of hypnozoites (the latent liver stages) of *P. vivax* and thus have potential to prevent relapses. Primaquine is the anti-relapse drug available in most endemic regions, but its widespread impact on health outcomes is limited by poor adherence to the standard 14-day course and concerns over the risk of drug-induced haemolysis in patients with glucose-6-phosphate dehydrogenase (G6PD) deficiency. Over the last few years, new tools and treatment strategies have been developed for better tolerated and more effective radical cure regimens. Short-course (7 days), high daily-dose primaquine has been shown to have similar efficacy compared with the same WHO-recommended total dose of 7mg/kg body weight administered over 14 days (Taylor W.J. et al. 2019) in areas with high risk of relapse. However, the higher daily dose in the 7 day regimen (1mg/kg) is associated with more adverse events, particularly an increased risk of haemolysis and gastrointestinal events. A semi-quantitative point-of-care G6PD test (SD Biosensor, ROK) has been developed and registered in Indonesia and the process of registration is underway in Papua New Guinea (PNG).

1.2 Purpose of the analyses

The aim of the SCOPE study is to determine the effectiveness and safety of introducing a revised case management package to facilitate well-tolerated and effective radical cure of patients with vivax malaria at 10 public health care facilities in Indonesia and Papua New Guinea.

The revised case management package has been prioritised and endorsed by the respective Ministries of Health and will include: 1) semi-quantitative point-of-care glucose-6-phosphate dehydrogenase (G6PD) activity assessment prior to treatment with primaquine, 2) prescription of high dose (7mg/kg body weight total) primaquine over 7 (**PQ7**), 14 (**PQ14**) days or 8 weeks (**PQ8W**), to all eligible patients presenting with *P. vivax* malaria, 3) patient education and counselling prior to treatment and supervision of the first dose of primaquine at the health care facility, 4) community-based clinical review at day 3 of primaquine treatment to encourage treatment adherence and facilitate detection and management of adverse events to treatment and 5) improved malariometric surveillance and pharmacovigilance.

2 Study Objectives and Endpoints

2.1 Study objectives

The overall objective of the SCOPE study is to determine the operational feasibility and cost-effectiveness of introducing the revised case management package for patients with vivax malaria to inform national antimalarial policy and practice.

This SAP is focused solely on the quantitative measures of operational feasibility, pharmacovigilance, safety and effectiveness in the Stage 1 and Stage 2 studies. A separate document will be prepared for the qualitative measures of operational feasibility and a health economics analysis plan (HEAP) will be prepared for the cost-effectiveness analyses.

2.1.1. Stage 1 – Preliminary safety and feasibility study

Primary objective:

S1.PO1 To determine the safety of PQ in patients prescribed different treatment regimens

S1.PO2 Determine the risk of patients experiencing any AESI during treatment

Secondary objectives:

S1.SO1 Determine the risk of patients experiencing severe GI AESI during treatment

S1.SO2 Determine the risk of patients experiencing severe haemolysis AESI during treatment

S1.SO3 Determine the risk of patients experiencing severe methaemoglobinaemia AESI during treatment

S1.SO4 Determine the tolerability of PQ in patients prescribed different treatment regimens

S1.SO5 Determine primaquine dosing accuracy by G6PD activity category

S1.SO6 Determine the proportion of patients receiving a clinical review on day 3 and day 7 of PQ treatment

2.1.2. Stage 2 – Large-scale implementation and feasibility study

Primary objective:

S2.PO1	To determine the operational feasibility of implementing revised case management for patients with *P. vivax* malaria.

Secondary objectives:

Quantitative measures of operational feasibility

S2.SO1	Determine the proportion of health care practitioners who comply with the revised radical cure treatment algorithm
S2.SO2	Determine the proportion of patients receiving a SD Biosensor G6PD test
S2.SO3	Determine primaquine dosing accuracy by G6PD activity category
S2.SO4	Determine the proportion of vivax malaria patients who are ineligible for daily primaquine and are incorrectly given primaquine (including infants, pregnant females and G6PD deficient patients)
S2.SO5	Determine the proportion of vivax malaria patients that are reviewed on day 3
S2.SO6	Determine the proportion of vivax malaria patients that adhere to their prescribed primaquine regimen

Pharmacovigilance

S2.SO7	Determine the proportion of community-based health workers who correctly act on early signs of haemolytic anaemia and GI events
S2.SO8	Determine the number of patients identified through the community surveillance system with serious adverse events

Safety

S2.SO9	Determine the safety of PQ under real-world conditions
S2.SO10	Determine the risk of patients experiencing at least one AESI during treatment
S2.SO11	Determine the risk of patients experiencing severe haemolysis AESI during treatment
S2.SO12	Determine the risk of patients experiencing severe GI AESI during treatment
S2.SO13	Determine the risk of patients experiencing severe methaemoglobinaemia AESI during treatment
S2.SO14	Determine the difference in severe anaemia prevalence between the pre- and post-implementation periods of the revised case management

Effectiveness

S2.SO15 Determine if the incidence of symptomatic *P. vivax* malaria decreases after the introduction of the revised case management

S2.SO16 Determine the difference in prevalence of *P. vivax* parasitaemia in patients presenting with fever between the pre- and post-implementation periods of the revised case management

S2.SO17 Determine if the cumulative risk of representation to the same health facility with symptomatic *P. vivax* malaria within 6 months differs between the pre- and post-implementation periods of the revised case management

S2.SO18 Determine if the cumulative risk of representation to the same health facility with symptomatic *P. vivax* malaria within 6 months differs between SCOPE and non-SCOPE patients in the post-implementation period of the revised case management

2.2 Endpoints

2.2.1. Stage 1 – Preliminary safety and feasibility study

All endpoints refer to patients enrolled into Stage 1 treated with daily or weekly PQ.

Primary endpoints:

S1.PE1 Proportion of patients experiencing at least one SAE during treatment.

S1.PE2 Proportion of patients experiencing at least one AESI, SAE or other AE of at least grade 3 during treatment.

Secondary endpoints:

S1.SE1 Proportion of patients with a gastrointestinal AESI during treatment.

S1.SE2 Proportion of patients with an AESI related to haemolysis during treatment.

S1.SE3 Proportion of patients with an AESI related to methaemoglobinaemia during treatment.

S1.SE4 Proportion of patients permanently stopping PQ before the end of treatment.

- Defined as the proportion of all patients enrolled who are given documented advice to permanently stop PQ during scheduled or ad hoc clinical review by a health professional

S1.SE5 Proportion of patients receiving correct treatment based on G6PD activity.

S1.SE6 Proportion of patients who were reviewed on day 3 and day 7.

Exploratory endpoints:

S1.EE1 Maximum absolute fall in haemoglobin within the first 7 days

S1.EE2 Maximum proportional fall in haemoglobin within the first 7 days

S1.EE3 Reduction of >25% in haemoglobin to a concentration of < 7 g/dl

S1.EE4 *POST HOC:* Level of agreement between SD Biosensor and HemoCue for haemoglobin measurement at study clinics and in the community

S1.EE5 *POST HOC:* Methaemoglobin concentrations on days 3 and 7

2.2.2. Stage 2 – Large-scale implementation and feasibility study

Primary endpoint:

S2.PE1 Proportion of patients with *P. vivax* malaria who correctly receive all components of revised case management (including G6PD testing, correct treatment of PQ according to revised case management and G6PD activity, patient education, supervision of the first dose and community review on day 3)

Secondary endpoints:

Quantitative measures of operational feasibility

S2.SE1 Proportion of health care practitioners who comply with the revised radical cure algorithm as defined below:

- Proportion of health care workers at the SCOPE health facility sites who correctly prescribe PQ (i.e. PQ7, PQ14 or PQ8W) according to the implementation package ≥95% of the time

S2.SE2 Proportion of patients eligible for PQ who are tested for G6PD activity using the SD Biosensor at the time of their diagnosis of malaria.

S2.SE3 Proportion of eligible vivax malaria patients receiving the correct PQ regimen based on the result of the SD Biosensor test.

S2.SE4 Proportion of vivax malaria patients who are ineligible for daily primaquine and are incorrectly prescribed an inappropriate primaquine regimen.

Ineligible being defined as:

- Infant <6 months or <5kg weight (Indonesia) or <1 year (PNG), pregnant women or women breastfeeding infants <6 months of age

(Indonesia) or <1 year of age in PNG. Should not receive any primaquine
- G6PD deficient (<30% enzyme activity). Should not receive daily primaquine.

S2.SE5 Proportion of vivax malaria patients that are reviewed on day 3.

S2.SE6 Proportion of vivax malaria patients that adhere to their prescribed primaquine regimen defined as follows:

- The proportion of patients who self-report that they have taken all prescribed PQ doses between the time of the initial prescription and the follow-up visit.

Pharmacovigilance

S2.SE7 Proportion of CHWs who correctly act on early signs of haemolytic anaemia and GI events defined as follows:

- Proportion of CHWs who refer 100% of patients documented as having a potential severe symptom (‘flag’) at day 3 review for level 2 clinical review
- Proportion of patients with a severe symptom (‘flag’) at day 3 review who are referred by a CHW for level 2 review

S2.SE8 Number of patients with a SAE who are identified by community or health facility staff follow-up and referred to hospital for further management.

Safety

S2.SE9 The proportion of patients eligible to receive PQ who had a serious adverse event (SAE) during treatment.

S2.SE10 The proportion of patients treated with PQ experiencing at least one AESI during treatment.

S2.SE11 The proportion of patients treated with PQ with an AESI related to haemolysis during treatment.

S2.SE12 The proportion of patients treated with PQ with a gastrointestinal AESI during treatment.

S2.SE13 The proportion of patients treated with PQ with AESI related to methaemoglobinaemia during treatment.

S2.SE14 Prevalence of severe anaemia (haemoglobin < 7g/dl) in patients presenting with fever in the Pre-Implementation Survey versus the late Post-implementation Survey (derived from the quantitative surveys).

- Median and distribution of haemoglobin concentrations will also be presented for each survey

Effectiveness

S2.SE15 The monthly incidence of confirmed symptomatic *P. vivax* malaria episodes (monoinfection or mixed) in the Pre-Implementation (12 months) versus Post-Implementation Stage 2 ((a) level change in 6 months post implementation (i.e. period between 6 and 12 months post-implementation), (b) change in trend of incidence immediately post-implementation (i.e. entire 12 month period post-implementation)).

S2.SE16 Prevalence of *P. vivax* parasitaemia in patients presenting with fever in the Pre-Implementation Survey versus the Post-Implementation Survey during Stage 2 (derived from the quantitative surveys).

S2.SE17 Cumulative risk of representation to the same health facility with symptomatic *P. vivax* malaria within 6 months in the Pre- and Post-Implementation Stages.

S2.SE18 Cumulative risk of representation to the same health facility with symptomatic *P. vivax* malaria within 6 months in the Post-Implementation Stage for patients enrolled and not enrolled in SCOPE.

Exploratory endpoints:

S2.EE1 The proportion of patients with a reduction of >25% in haemoglobin to a concentration of < 7 g/dl

3 Study Methods

3.1 Study design

This is a staged, multicentre, implementation, before-and-after study of a revised case management package for patients with vivax malaria presenting to community health facilities in Indonesia and Papua New Guinea.

The study will be conducted at 10 pre-existing health facilities, 4 in Papua New Guinea (two in stage 1) and 6 in Indonesia (two in stage 1), all of which have significant experience managing patients with vivax malaria.

All of the study health facilities will collect surveillance data that are reported as part of national malaria surveillance systems. These data include patient demographics, diagnosis and treatment. Prior to the start of the project, selected sites have undergone several rounds of continuous quality improvement (CQI) to improve the quality of data collection. Further enhancements to malariometric surveillance and pharmacovigilance will be made as part of the revised case management with a plan to roll these changes out on a wider scale if the changes prove feasible and cost-effective.

The study will be divided into multiple stages (Figure 1). Prior to commencement of Stage 1, enhanced continuous malariometric surveillance and pharmacovigilance will be established at all 10 health facilities. These systems have already been established in 4 sites in PNG and 4 in Papua with more

than 12 months of data available. Similar surveillance systems will also be established in North Sumatra and Lampung.

Within the first three months of the SCOPE pre-implementation period, in-depth interviews with key stakeholders and community surveys will be conducted to determine the acceptability, operational feasibility, performance and costs of existing vivax malaria management strategies, as well as baseline *P. vivax* slide or RDT positivity rates. Qualitative, economic and clinical data collection will not necessarily happen simultaneously.

Midway through the Pre-Implementation Stage, consecutive patients (within the bounds of participant accrual limits) presenting to each health facility with fever will be asked to participate in the Pre-Implementation Survey aiming for a total of 200 patients at each facility. Informed consent and assent for participation will be requested. A repeat cross-sectional survey of 200 consecutive patients presenting to each health facility with fever will then be conducted in the final 6 months of the post-implementation period for comparative purposes.

3.1.1. Stage 1 – Preliminary safety and feasibility study

In Stage 1, the feasibility, safety and tolerability of point-of-care G6PD testing and PQ7 in G6PD normal (>70%) patients will be determined in 4 selected health facilities with staff undertaking clinical review on day 3 and also day 7 to ensure patient safety. Stage 2 will only proceed after review of the Stage 1 data by SRT and SMC.

After 3 months, the revised case management will be rolled out at 4 selected sites (2 in Indonesia and 2 in PNG), initiating Stage 1. The remaining sites will continue the Pre-Implementation Stage. Stage 1 is anticipated to last 6 months, during which a total of 800 patients with *P. vivax* malaria (~500 from Indonesia and ~300 from PNG) will be treated (Figure 1).

All patients that are eligible for and consent to participate in the study, will be tested by quantitative G6PD activity assessment using the G6PD STANDARD (SD biosensor, ROK). Patients with G6PD activity >70% (≥6.1 U/gHb) will be prescribed primaquine at a total dose of 7 mg/kg over 7 days (PQ7) and those with 30-70% activity (4.1-6.0 U/gHb) will be prescribed the same total dose of primaquine over 14 days (PQ14). Patients with G6PD activity <30% (≤4.0 U/gHb) will be prescribed primaquine at a dose of 0.75 mg/kg/week for 8 weeks (PQ8w).

The safety review team (SRT) will review all AESI (adverse event of special interest) and SAE (severe adverse event) data after every 100 patients treated with primaquine and may request to review additional data if there are safety concerns. A formal interim analysis is planned after enrolment of 400 patients (~150 patients from PNG, ~250 patient from Indonesia). The SRT will review the interim analysis outputs and share with the Safety Monitoring Committee (SMC) who will conduct an interim review and issue a recommendation regarding continuation of Stage 1.

At the end of Stage 1, once 800 patients (~300 patients from PNG, ~500 patients from Indonesia) have been enrolled, a final analysis will be conducted and reviewed by the SRT and presented to the Safety Monitoring Committee. We anticipate that 700 patients will have been treated with PQ7 and 100 with PQ14. Prior to study start the SMC will be tasked with defining specific *a priori* criteria that will be used to decide whether the study progresses directly to the main feasibility study for wider roll out in Stage 2 without changes in the protocol, whether the implementation package needs to be revised or if the study needs to be discontinued. The final analysis of Stage 1 will be reviewed by the SRT and summarized to support the SMC final review. If the SMC recommends progression to Stage 2, the data

summary, together with the SMC recommendations will be shared with the NDoH and NDA for approval to progress to Stage 2.

3.1.2. Stage 2 – Large-scale implementation and feasibility study

Stage 2 of the study will be a feasibility study conducted at 10 community health-care facilities: 6 sites in Indonesia and 4 sites in Papua New Guinea. The primary purpose of Stage 2 is to determine the feasibility and cost-effectiveness of the complete revised case management initially studied in Stage 1. Robust Pre-Implementation malariometric surveillance for a minimum of 12 months along with quantitative surveys of baseline operational metrics and costs will be followed by coordinated introduction of the revised case management across all study health facilities and a minimum of a further 12 months of surveillance – creating an interrupted time series comparing the endpoints listed in section 2.2.2.

During Stage 2 study procedures will be the same as those for Stage 1, except that the procedures will be performed by routine healthcare staff. All patients will be reviewed by community health workers (CHW) at home 3 days after starting PQ. The review may also take place at the health facility, if the patient presents there. Exceptionally, the follow-up may also take place via a phone call if the patient is not physically reachable at home. The CHW will complete a signs and symptom checklist to detect early indicators of haemolysis or other AESI. Patients with signs of haemolysis or other AESI will have their primaquine ceased and will be referred to the health facility for medical review. Patients tolerating their medication will be encouraged to adhere to a full (7 or 14 days) course of treatment. Safety outcomes occurring between day 3 and end of treatment will be detected passively by community-based health workers, study health facilities and regional hospitals.

Continuous surveillance and pharmacovigilance over the entire study period will enable interrupted time-series analyses of malariometric indices (see section 2.2.2).

Figure 1 presents the study schedule for the baseline data, pre-implementation stage, and stages 1 and 2.

Figure 1. Study overview

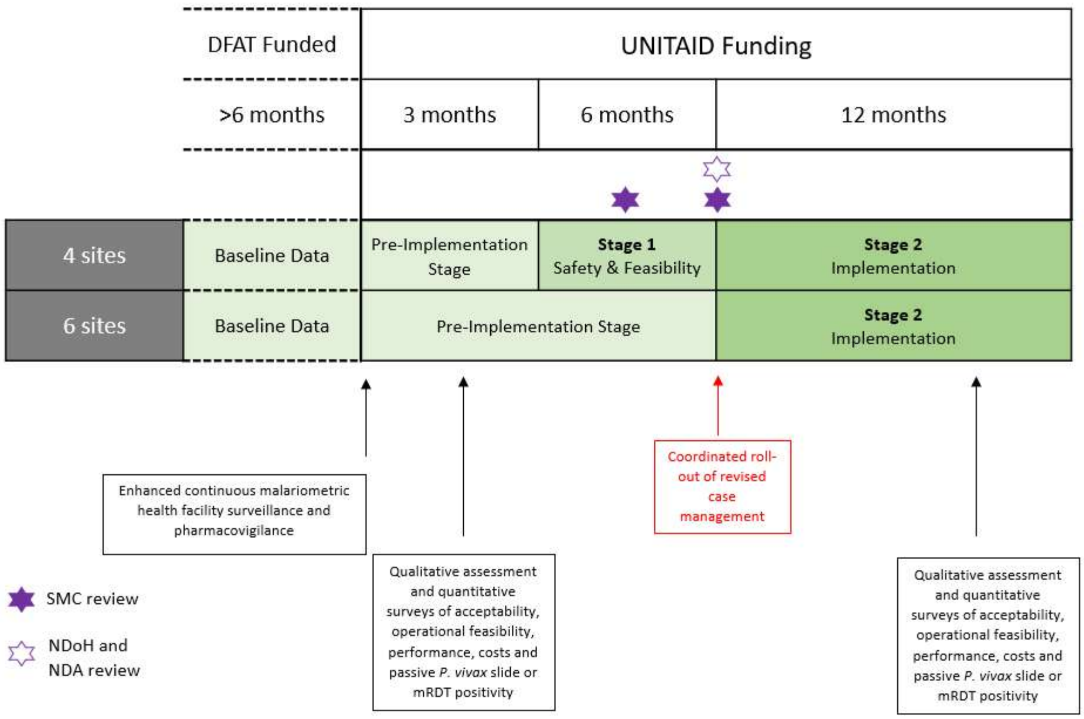

3.2 Inclusion and exclusion criteria

Inclusion criteria Stage 1 of the study (and thus receipt of either PQ7, PQ14 or PQ8w) are: age >1 year (PNG) or ≥6 months (Indonesia), haemoglobin concentration ≥8 g/dl and signed agreement of patient or their carers to participate in the study and comply with all study-specific obligations, including attending follow-up clinical evaluations (at day 3 and day 7). Inclusion criteria for Stage 2 will be the same except that patients will only need to be able to attend a follow-up appointment (or receive a phone call) on day 3.

Exclusion criteria: pregnant and lactating females with infants <6 months in Indonesia and <1 year in PNG and patients with severe manifestations of malaria will not be prescribed primaquine and will not be enrolled in Stage 1. These patients will contribute incidence data to Stage 2 of the study but will not receive primaquine treatment. Patients' ineligible for primaquine treatment will be treated according to current national guidelines.

4 Sample size

4.1 Stage 1

In the Stage 1 preliminary study of the feasibility and safety of the implementation package, 4 health facilities will enrol 800 patients (~500 in Indonesia and ~300 in Papua New Guinea). Assuming 764 of these patients will have a G6PD activity >70% and be eligible for PQ7, and that the proportion of patients receiving short-course primaquine who have to stop therapy within 7 days is 5%, this sample size will produce a 95% confidence interval for the true proportion needing to stop treatment of +/- 1.55%.

4.2 Stage 2

NOTE: *Given slower than expected recruitment in Stage 1, the predicted patient recruitment in Stage 2 was revised down on 5 August 2024 to 70% of eligible patients (n=11,410) across Indonesia and Papua New Guinea. Additionally, an error was detected in the original power calculation for detection of a reduction in incidence of vivax malaria. In protocol vs 5.0, the total number of patients with vivax malaria recruited into the study was erroneously predicted to be 16,300 over 18 months (the initial timeframe for the study) when in reality this was the number of patients predicted over 12 months. This resulted in an underestimate of the monthly case incidence at each clinic and thus an underestimate of the power available for detection of an incidence reduction.*

The revised and corrected power and precision calculations are provided below with the original erroneous calculations retained for reference. Power calculations for detection of an individual-level reduction in risk of vivax malaria recurrence are also provided.

4.2.1 Revised and corrected Stage 2 power and precision calculations

Assumptions underlying the following Stage 2 power calculations include the following:

1. 5% of patients are ineligible for the intervention package (pregnancy, lactation, severe malaria, age <6m (Indonesia) or <1 yr (PNG)).
2. The impact on the risk and rate of infection will be the same across different endemic sites.
3. The mean baseline risk of *P. vivax* recurrence at 6 months is 20%
4. Loss to follow-up in both groups is 10%
5. Alpha = 0.05

During the 12-month pre-implementation period, a total of **17,160** patients with *P. vivax* (mono or mixed infections) presented to the ten study clinics (see Table 1). Assuming 5% of patients are ineligible for enrolment due to pregnancy, lactation or age under 6 months (Indonesia) or 12 months (PNG), this gives an estimated maximum number of patients eligible for recruitment into Stage 2 of **16,300**.

Table 1 Number of patients with vivax malaria attending the ten study clinics in last 12 months

Clinic name	Country	Locality	Estimated total number of *P. vivax* episodes per year	Estimated maximum recruitment in SCOPE Stage 2 (95%)
**Timika**	Indonesia	Timika, Southern Papua	3173	3014
**Bhintuka**	Indonesia	Timika, Southern Papua	1164	1106
**Pasar Sentral**	Indonesia	Timika, Southern Papua	4937	4690
**Wania**	Indonesia	Timika, Southern Papua	3681	3497
**Tanjung Leidong**	Indonesia	North Sumatra	500	475
**Hanura**	Indonesia	Lampung	450	428
**Baro**	PNG	West Sepik	417	396
**Mugil**	PNG	Madang	1044	992
**Napapar**	PNG	East New Britain	412	391
**Wirui**	PNG	East Sepik	1382	1313
		Total	17,160	**16,302**

Footnotes: Estimates based on previous clinic records and number of patients with *P. vivax* recruited over last 12 months. Maximum enrolment assumes 5% of patients are ineligible because they are pregnant, lactating or under 6-12 months of age.

S2.SE15 - Incidence Rate Reduction

The proportion of patients recruited into SCOPE has been lower than expected and this will impact on the predicted reduction in incidence. The revised estimated reduction in incidence and predicted power to show this are provided in Table 2, with varying levels of recruitment (0.5-1).

Table 2 – Power to detect a reduction in the incidence of *P. vivax* malaria

Proportion of eligible *P. vivax* malaria patients recruited	Eligible patients over 12 months period	Predicted mean monthly incidence at each clinic preintervention	Predicted absolute incidence reduction	Predicted mean monthly incidence at each clinic post intervention	Power to detect predicted incidence reduction
1	16,300	135.8	24.44	111.36	99.5%
0.9	14,670	135.8	22.00	113.80	98.4%
0.8	13,040	135.8	19.56	116.24	95.5%
0.7	11,410	135.8	17.11	118.69	**89.3%**
0.6	9,780	135.8	14.67	121.13	78.6%
0.5	8,150	135.8	12.22	123.58	63.2%

Example STATA code: power pairedm 135.8 111.36, sddiff(15) n(10) alpha(0.05).

Based on previous estimates, 60% of cases of *P. vivax* malaria seen at each clinic are due to relapse and therefore preventable with improved radical cure. We predict the intervention package will reduce the individual risk of relapse by 30%. With anticipated recruitment of 70% of eligible patients, the total predicted sample size in Stage 2 would fall to **11,410** patients receiving primaquine over 12 months of enrolment. Based on this lower predicted enrolment and using the assumptions as above, the predicted absolute reduction in incidence at each clinic would be 17.11 cases per month ((135.8*0.6)*0.3*0.7) and resultant power to detect this difference would be **89.3%.**

S2.SE17 - Reduction in individual risk of recurrence

The power estimates for detecting a reduction in the individual risk of recurrence of *P. vivax* within 6 months from an anticipated study enrolment of **11,410 patients** are presented in Table 3. The number of patients included in the pre-implementation and implementation stages are assumed to be equal (ie a total of 22,820 patients pre and post implementation).

Table 3 – Power to detect reduction in the risk of recurrence

Proportion of eligible *P. vivax* malaria patients recruited	Eligible patients over 12 months period	30% Risk Reduction (HR 0.7)	20% Risk Reduction (HR 0.8)	10% Risk Reduction (HR 0.9)
1	16,300	100%	100%	98%
0.9	14,670	100%	100%	97%
0.8	13,040	100%	100%	95%
0.7	11,410	100%	100%	**92%**
0.6	9,780	100%	100%	88%
0.5	8,150	100%	100%	82%

Example STATA code: artsurv, edf0(0.2) hr (1,0.7) fp(1) n(22,820) alpha(0.05) nperiod(1) tunit(2) lg(1 2) ldf(0.1;0.1).

A sample size of 11,410 (70% eligible patients) would provide **92% power** to detect a minimum 10% reduction in risk of recurrence and 100% power to detect a 30% reduction in risk of recurrence.

S2.PE1 Proportion of patients with *P. vivax* malaria who correctly receive all components of revised case management

Assuming 70% of patients enrolled into Stage 2 receive all components of the implementation package correctly, the revised Stage 2 sample size of 11,410 patients will give a 95% confidence interval for the true proportion of +/- 0.84%.

S2.SE9 Safety and protocol adherence outcomes

Assuming 1% of patients have a treatment-limiting haemolytic event, the revised Stage 2 sample size of 11,410 patients will give a 95% confidence interval for the true proportion of +/- 0.18%.

4.2.2 Original power and precision calculations – now superseded

Indonesia and PNG combined study

The Stage 2 implementation study will be conducted at ten health facilities across Indonesia and Papua New Guinea. Based on prior surveillance, we estimate that over 18 months, these health facilities will enrol approximately 16,300 patients with vivax malaria. Improved radical cure of *P. vivax* malaria is expected to reduce the risk of subsequent relapse and therefore reduce the overall incidence of vivax malaria. Across the 10 health facilities, a mean of 91 patients with vivax malaria are expected to be seen each month, 60% (n=54.3) of which are predicted to be relapses and therefore feasibly preventable. Assuming that the implementation package in Stage 2 will reduce relapses by 30%, the monthly incidence of *P. vivax* malaria is expected to fall by 16.3 (17.9%) to 74.7 cases per month. With a two-sided alpha of 0.05 and a predicted standard deviation of the mean variation in monthly numbers of vivax malaria of 15 episodes per month, our study would achieve 86% power to detect an 18% reduction in monthly *P. vivax* malaria incidence. Furthermore, assuming 70% of patients receive all components of the implementation package correctly, this sample size will give a 95% confidence interval for the true proportion of +/- 0.70%. If 1% of patients are predicted to have a treatment-limiting haemolytic event, this sample size will be powered to determine the true proportion within +/- 0.15%.

Indonesia and PNG standalone studies

In the unlikely scenario that either Indonesia or PNG will not proceed, the study will become a standalone country study. In this case the sample size calculations are as follows. In a standalone Indonesia study, Stage 1 will occur in the same two health facilities as outlined above. However, we will enrol 800 patients to retain the sample size of the combined study. Stage 1 will still produce a 95% confidence interval for the true proportion needing to stop treatment of +/- 1.55%. In Stage 2, the implementation study will be conducted at six health facilities in Indonesia. Based on prior surveillance, we estimate that over 12 months, these health facilities will enrol approximately 10,750 patients with vivax malaria. Across the 6 health facilities, a mean of 60 patients with vivax malaria are anticipated each month, 60% (n=36) of which are predicted to be relapses and are therefore preventable. Assuming that the implementation package in Stage 2 will reduce relapses by 30%, the monthly incidence of *P. vivax* malaria is expected to fall by 10.7 (17.9%) to 49.0 cases per month. The Indonesian study alone would retain 80% power to detect a 36% overall reduction in incidence following implementation of the interventions. Furthermore, assuming 70% of patients receive all components of the implementation package correctly, this sample size will give a 95% confidence interval for the true proportion of +/- 0.87%. If 1% of patients are predicted to have a treatment-limiting haemolytic event, this sample size will be powered to determine the true proportion within +/- 0.19%.

In PNG as a standalone study, Stage 1 will occur in two health facilities, as outlined above, however we will enrol 800 patients to retain the sample size of the combined study. Stage 1 will still produce a 95% confidence interval for the true proportion needing to stop treatment of +/- 1.55%. In Stage 2, the implementation study will be conducted at four health facilities in PNG. Based on prior surveillance, we estimate that over 12 months, these health facilities will enrol approximately 5,550 patients with vivax malaria. Improved radical cure of *P. vivax* malaria is expected to reduce the risk of subsequent relapse and therefore reduce the overall incidence of vivax malaria. Across the 4 health

facilities, a mean of 77 patients with vivax malaria are anticipated each month, 60% (n=46.3) of which are predicted to be relapses and are therefore preventable. Assuming that the implementation package in Stage 2 will reduce relapses by 30%, the monthly incidence of *P. vivax* malaria is expected to fall by 13.8 (17.9%) to 63.2 cases per month. The PNG arm of the study would retain 80% power to detect a 42% overall reduction in incidence following implementation of the interventions. Furthermore assuming 70% of patients receive all components of the implementation package correctly, this sample size will give a 95% confidence interval for the true proportion of +/- 1.21%. If 1% of patients are predicted to have a treatment-limiting haemolytic event, this sample size will be powered to determine the true proportion within +/- 0.26%.

Results of the power calculations for the effectiveness endpoints provided above have been confirmed using numerical simulations. A total of 1000 simulated datasets from 10 health facilities, with 18 months of observations and 91 infections per month (in the pre-intervention phase) were generated. Between health-facility variation in the outcome was based on an intra-cluster correlation coefficient of 0.1, and within health-facility variation was assumed to have a standard deviation of the mean variation in monthly numbers of vivax malaria of 15 episodes per month.

5 Analysis Objectives

5.1. Stage 1 – Preliminary safety and feasibility study

The analysis objectives for Stage 1 are to estimate the proportion of patients with the primary and secondary endpoints (i.e. safety, tolerability and clinical review endpoints) provided in section 2.2.1.

Following accrual of anecdotal evidence of some seemingly erratic haemoglobin results from the SD Biosensor during the early phase of the Stage 1 study, a decision was made to test haemoglobin in duplicate using the bedside HemoCue assay, where available, to ensure patient safety and to provide a means of assessing the reliability of the SD Biosensor. A relevant *post hoc* exploratory endpoint (S1.EE4) has been added to this Statistical Analysis Plan and details of analytical methods are provided below.

Methaemoglobin concentration on days 3 and 7 has recently been proposed as a surrogate marker of primaquine anti-hypnozoite activity (Fadilah I. et al. 20204). Following two reports of adverse events in Papua (low oxygen saturation and elevated methaemoglobin), methaemoglobin began to be measured routinely on days 3 and 7 in Papua midway through Stage 1. Methaemoglobin concentration has therefore been added as a relevant *post hoc* exploratory endpoint (S1.EE5).

5.2. Stage 2 – Large-scale implementation and feasibility study

The primary analysis objective for Stage 2 is to estimate the proportion of patients with *P. vivax* malaria who correctly receive all components of revised case management (including G6PD testing, correct treatment with primaquine according to revised case management and G6PD activity, patient education, supervision of the first dose and community review at day 3), as stated in section 2.2.2. The secondary analysis objectives for Stage 2 include estimating the proportion of patients with secondary safety, tolerability and clinical review endpoints. The effectiveness and safety of implementing revised case management for patients with *P. vivax* malaria will also be estimated using an interrupted time series design for the incidence of symptomatic *P. vivax* malaria episodes, survival analysis of the cumulative risk of representation with *P. vivax* malaria and before versus

after analyses of the prevalence of asexual *P. vivax* parasitaemia and severe anaemia in patients presenting to health facilities with fever.

All of the above estimates will be presented with 95% confidence intervals.

6 Analysis sets/Subgroups

6.1. Stage 1 – Preliminary safety and feasibility study

The primary analyses of the endpoints in Stage 1 will include data from all patients enrolled across the four sites in PNG and Indonesia. Secondary analyses of patient-related study outcomes will be conducted overall and for the following prespecified subgroups:

- Patients from Indonesia, Papua New Guinea
- Study site
- Ethnic group
- Patients with G6PD activity >70%, ≥30% to ≤70% and <30%
- Patients prescribed PQ7, PQ14 and PQ8W
- Patients aged <5 years, 5-15 years and >15 years
- Males and females

6.2. Stage 2 – Large-scale implementation and feasibility study

The primary analysis of the endpoints in Stage 2 will include all patients enrolled across the 10 health facilities in Indonesia and Papua New Guinea from the start of the pre-implementation period to the end of the post-implementation period (estimated to be about 16,300 patients). Before versus after analyses of the prevalence of asexual *P. vivax* parasitaemia and severe anaemia from the cross-sectional surveys will be done on the full aggregated dataset of all patients enrolled in the surveys across the 10 study sites (aiming for 200 patients each health facility for each survey = 4,000 patients total).

Secondary analyses of patient-related study outcomes will be conducted for the following pre-specified subgroups:

- Patients from Indonesia & Papua New Guinea
- Patients from PNG, Indonesian Papua and Sumatra
- Study site
- Ethnic group
- Patients with G6PD activity >70%, ≥30% to ≤70% and <30%
- Patients prescribed PQ7, PQ14 and PQ8W
- Patients aged <5 years, 5-15 years and >15 years
- Males and females

All of the health facilities in the study are already collecting *P. vivax* malaria incidence data.

7 Outcomes and Covariates

The primary, secondary and exploratory outcomes for Stage 1 and Stage 2 are defined in Section 2.2 (Endpoints).

Intervention and covariate variables for analyses of the impact of the implementation package on incidence of symptomatic *P. vivax* malaria (effectiveness outcome S2.SE15) will be defined as follows:

a) **Level change** intervention effect (i.e. change in mean monthly incidence) 6 months after the start of stage 2; Figure 2, Section 9.
b) **Slope change** intervention effect (i.e. change in the rate of change of monthly incidence) immediately after the start of stage 2; Figure 2, Section 9.

Intervention will take the value of 1 for all time points following the start of stage 2 implementation at each health facility site, and a value of 0 for all other timepoints for both the level and slope changes.

The time month covariate will be defined as the number of months after the start of stage 2 implementation (Appendix 4: Figure 1), i.e. negative values for pre-implementation and positive values for post-implementation.

Seasonality will be captured using spline terms for the Indonesian health facility sites and the PNG health facility sites.

Any unexpected stock outages or major, discrete malaria control interventions external to the SCOPE project will also be captured and reported by health facility site and calendar time month.

The key covariates that will be included in the analyses of the safety and exploratory outcomes will be the haemoglobin concentration at enrolment, the mg/kg dose of primaquine and the G6PD enzyme activity. Key covariates to be adjusted for in the analysis of patient-level effectiveness outcomes will be baseline age, weight and sex, except in the case that these variables have already been considered in a propensity score matching process.

8 Handling of Missing Values

We do not expect there to be any reason that calendar time month will be missing for any observations over the study period (pre-implementation, Stage 1 and Stage 2) at each of the 10 sites. This is based on our experience of routine data collection during the baseline data period (i.e. prior to pre-implementation stage). We will descriptively report the patterns of missingness for the outcome measures and other participant variables over time in each site. For all analyses, missing data will be handled using an available case analysis, i.e. missing data on any variable included in the analysis where the observation for an individual at that time point is missing is excluded from the analysis.

9 Statistical Methodology

Continuous surveillance and pharmacovigilance data will be summarised and presented to study staff in graphical and descriptive text format on a monthly basis to enable real-time feedback on case numbers and distributions, data integrity, clinical performance and revised case management adherence.

9.1. Stage 1 – Preliminary safety and feasibility study

For Stage 1, the number and proportion of patients experiencing at least one SAE (S1.PE1) or at least one AESI (S1.PE2) will be computed by treatment arm (PQ7, PQ14, PQ8w), and described in summary tables which will include breakdown by type of event, severity, relatedness to treatment, and timing of occurrence (see Appendix 4 for example Tables 1-4). The number and proportion of patients permanently discontinuing treatment (S1.SE4) will be computed by treatment arm and presented in a summary table, with breakdown of reason for discontinuation. Absolute numbers of AESIs with denominators (S1.PE1-3) and proportions with 95% confidence intervals (calculated using a binomial distribution) will be presented in tabular and graphical format. A full tabulation of all SAEs will also be presented.

For S1.EE3, the number and proportion (and 95% Confidence Interval) of patients observed to have a >25% reduction in haemoglobin to a concentration below 7 g/dl will be computed by treatment arm (PQ7, PQ14, PQ8w), and described in summary tables.

For S1.EE1 and S1.EE2, the unadjusted and adjusted mean absolute and proportional maximum change in haemoglobin within 7 days (95% CI) respectively will be presented by treatment arm (PQ7, PQ14, PQ8w). The adjusted means will be estimated from a linear regression model that adjusts for baseline haemoglobin level and G6PD enzyme activity level. The association between change in haemoglobin from baseline to day 2 and G6PD enzyme activity level will be estimated from a linear regression model that allows for a nonlinear association using fractional polynomials and adjusts for baseline haemoglobin level, age, sex and baseline parasitaemia. A scatterplot of the data will be presented with observations labelled by treatment arm (PQ7, PQ14, PQ8w).

For S1.EE4, the mean difference, standard deviation of the difference and 95% limits of agreement between Biosensor and HemoCue haemoglobin measurements will be computed using Bland-Altman plots (Bland JM & Altman DG 1995), stratified by day of measurement (day 0, 3, 7) and country. The data will be presented in tabular and graphical formats.

For S1.EE5, the distribution of methaemoglobin at baseline, day 3 and day 7 will be presented by treatment arm (PQ7, PQ14, Pq8w) using box and whisker plots. The distribution of methaemoglobin levels at day 7 will be compared between the PQ7 (daily PQ dose of 1mg/kg) and PQ14 (daily PQ dose of 0.5 mg/kg) treatment arms using linear regression after adjusting for baseline haemoglobin concentration, parasitaemia, age, sex, and G6PD enzyme activity level.

9.2. Stage 2 – Large-scale implementation and feasibility study

For Stage 2, the proportion of eligible patients receiving all elements of the implementation package correctly, and other related outcomes, will be presented for all patients, as well as stratified by study country and health facility. Corresponding 95% confidence intervals of these outcomes will be calculated assuming a binomial distribution.

The change in the incidence of symptomatic *P. vivax* malaria episodes (S2.SE15 - effectiveness endpoint) between the pre-Implementation Stage and post-implementation stage (Stage 2) will be shown graphically using an interrupted time series graph. Following the recommendations of Turner et al. (2020) the following components will be displayed: the health facility site-level (aggregated) data points versus time point of measurement (e.g. monthly for primary outcome) as a scatter plot; the interruption time will be displayed with a vertical dashed line; and the pre-interruption and post-

interruption trend lines; and the counterfactual trend line (an example is given in Appendix 3, Figure 2).

The estimate of the effect of the implementation package will be determined by fitting a segmented regression model to the monthly health facility level data (Bernal J.L. et al. 2017). For the segmented regression model, we will estimate the impact of the revised case management as:

a) a level change comparing six months post-implementation to pre-implementation stage (Figure 2).

b) the difference in trends before and after introduction of the implementation package (i.e. slope change comparing post-implementation to pre-implementation stage) (Bernal J.L. et al. 2017) (Figure 2).

A binary indicator variable will take the value of 1 for all time points following the start of the stage 2 implementation package within each health facility site, and a value of 0 for all other timepoints. Health facilities also participating in stage 1 will have this data excluded from analysis as seen in Appendix 4: Figure 1. A sensitivity analysis will be performed where the binary indicator variable takes the value of 1 for all timepoints following the start of implementation, including both stage 1 and 2.

See Appendix 4: Figure 1 for the data to be included in the pre- and post-implementation periods for S2.SE15.

The model will include time (month) as a continuous variable, a binary variable for the implementation intervention (see (a) and (b) above), an interaction between elapsed time (since implementation) and the implementation, seasonal variation in malaria (captured using splines), adjusted for potential time-varying confounding variables including large scale malaria control activities outside of the SCOPE study and unexpected antimalarial stock outages, and allowing for a random effect for intercept to account for clustering by health facility site (Turner SL et al. 2021).

For the effectiveness outcome S2.SE15, monthly incidence of symptomatic *P. vivax* malaria will be calculated using clinic catchment populations provided by health facilities (or if not available, the most recent census). Incidence rate ratios (IRRs) (95% CIs) for the intervention effect will be estimated from a negative binomial regression model fitted to the monthly aggregated data.

Figure 2. Example impact model for the implementation package on the effectiveness outcome measure S2.SE15 (incidence of symptomatic *P. vivax* malaria infections). An impact model with an immediate change in level and slope. While immediate level change is allowed to occur in this model at the time of implementation, the estimand of interest with regards to level change will be calculated 6 months after implementation (at 12 months) relative to the implementation timepoint (at 6 months).

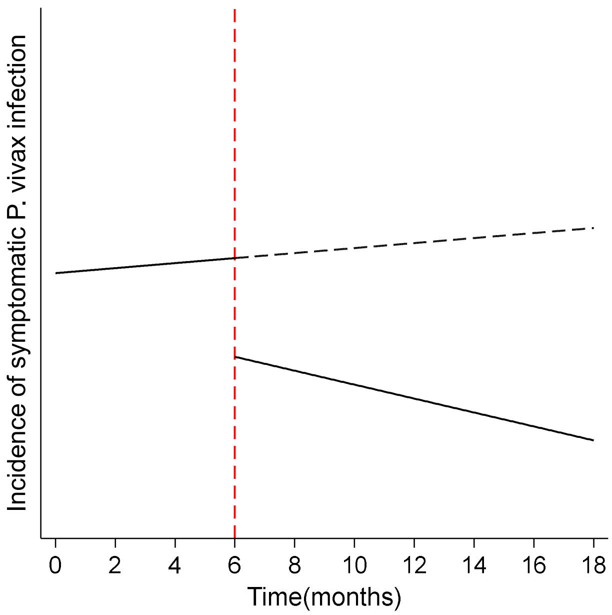

For comparison of outcomes S2.SE16 (the proportion of febrile patients with asexual *P. vivax* parasitaemia by microscopy) and S2.SE14 (the proportion of patients with severe anaemia (haemoglobin < 7 g/dl), determined from cross-sectional surveys in the pre- and post-implementation periods), prevalence ratios (95% CIs) for the intervention effect will be estimated from log-binomial regression models, and if there are convergence issues, Poisson regression with robust variance will be performed (Barros AJD & Hirakata VN 2003).

Individual patient data on the risk of representation with vivax malaria within 6 months to the same health facility site (S2.SE17) will be analysed using survival analysis and visualised with Kaplan-Meier curves. The risk of recurrence will be determined for all patients eligible for PQ treatment and compared between the pre- and post-implementation stages. Multivariable Cox proportional hazards regression will be used to compare these same groups, controlling for age, sex, body weight, calendar time (month), seasonality (captured using splines) and shared frailty of health facility. The validity of the proportional hazards assumption will be assessed visually using Schoenfeld residuals and stratified analyses will be conducted where the assumption is violated.

For S2.EE1, the number and proportion (and 95% Confidence Interval) of patients observed to have a >25% reduction in haemoglobin to a concentration below 7 g/dl will be computed by treatment arm (PQ7, PQ14, PQ8w), and described in summary tables.

Clustering

The relevant hierarchy of observations is that observations over time are clustered within health-facility. This clustering will be explored in analyses of the effectiveness outcomes using either random effects or robust standard errors for clustering by health facility.

9.3 Measures to Adjust for Confounders, Heterogeneity and Autocorrelation

Confounders

Both pre- and post-implementation surveillance periods will be of sufficient duration to minimise the risk of outlier seasonal and social events on the incidence of vivax malaria. Multivariable models, controlling for known potential confounders such as sex, age, weight, calendar month and seasonality will be used for comparisons of malaria incidence, slide positivity and other indices. For the exploratory outcomes, S1.EE1 and S1.EE2 multivariable linear regression models will be adjusted for known potential confounders, baseline haemoglobin levels and G6PD enzyme activity level.

Heterogeneity

The mixed effects models allow for some heterogeneity in slope and intercept at the health facility level. A visual assessment of heterogeneity of intervention effect according to site characteristics will be performed.

Autocorrelation

Autocorrelation for the segmented mixed-effects regression analyses estimating the effectiveness of the implementation package will be assessed visually by plotting the residuals (difference between observed and model predicted outcome) over time for each model.

10 Sensitivity Analyses

The following sensitivity analyses will be performed for the analysis of the S2.SE15 effectiveness outcome (incidence symptomatic *P. vivax* malaria episodes);

- The analysis will include incidence data from stage 1 implementation in addition to stage 2 in the post-implementation period for the ITS analysis (Appendix 4: Figure 2).
- The analysis will be performed with alternative adjustments for seasonality
    - Adjustment with a categorical rainy/dry seasonality variable derived specifically for each site

The following sensitivity analyses will be performed for the analysis of the S2.SE18 effectiveness outcome (cumulative risk of recurrence);

- The analysis will be restricted to Indonesian patients who have a malaria card number recorded. It is thought that patients without a malaria card no. in Indonesia tend to be part of a more transient population with different health-seeking behaviours.

11 Exploratory analyses comparing patients enrolled and not enrolled in SCOPE

The impact of the intervention on the risk of recurrence is assessed by Endpoint S2.SE17. At the start of Stage 2, it was apparent that the total sample size enrolled into the SCOPE study would be lower than anticipated. Additional analyses were added to the SAP to explore this endpoint with new endpoint S2.SE18.

S2.SE18: Cumulative risk of representation to the same health facility with symptomatic *P. vivax* malaria within 6 months in patients enrolled and not enrolled into the SCOPE Study. Comparisons made in the Post-Implementation Stage only.

Dataset for Analysis

Endpoint S2.SE18 will include all SCOPE patients eligible for PQ treatment and a selected dataset of non-SCOPE patients who were treated with PQ treatment for *P. vivax* malaria according to national guidelines. SCOPE patients from both stage 1 and stage 2 will be included. The non-SCOPE patients will be eligible for selection if they were not enrolled in SCOPE at any stage (based on malaria card number and other identifying factors) and were administered the PQ dose according to national guidelines and met the SCOPE eligibility criteria: age >1 year (PNG) or ≥6 months (Indonesia, haemoglobin concentration ≥8 g/dl, and not pregnant or lactating and did not have severe malaria. Day 0 for non-SCOPE patients will be taken as the first visit by a patient following the stage 1 implementation date.

In the scenario that the baseline characteristics differ substantially between the SCOPE and non-SCOPE patients, a subgroup of these non-SCOPE patients will be selected for analysis using propensity score matching with the SCOPE patients. This will be done separately by country (PNG and Indonesia) and will be based on the following variables:- age, sex, body weight. Propensity score matching is a valuable way to control for bias and achieve pseudo randomisation in this exploratory analysis which is comparing SCOPE patients to routinely collected patient data.

Statistical Analysis

A flowchart will be provided for the non-SCOPE patients, presenting the number of patient data available, eligible/non-eligible for selection, the reason for being non-eligible, and if applicable, the numbers selected/not-selected based on propensity score matching with SCOPE patients.

The number (proportion) of SCOPE patients and non-enrolled patients (if applicable 'propensity-score matched (PSM) selected') will be presented stratified by country and health facility. The distribution of age, sex, body weight and primaquine dose will be presented in a Table for SCOPE patients (enrolled in post-implementation stage), and non-enrolled eligible patients (and if applicable, non-enrolled PSM-selected patients) to assess the balance of the distribution of age, sex and body weight.

If applicable, to determine the PSM selected patient dataset, the propensity score for SCOPE versus non-SCOPE eligible patients will be estimated using logistic regression with covariates age, sex, and body weight. This regression analysis will be conducted separately by country. We will then use the estimated propensity scores from each of these analyses separately for matching without replacement 'm' non-enrolled eligible patients to each SCOPE patient based on a distance that corresponds to <=0.2 standard deviations of the estimated log odds of the estimated propensity score.

The risk that patients represent with *P. vivax* malaria within 6 months to the same health facility site (S2.SE18) will be analysed using survival analysis and visualised with Kaplan-Meier curves. The risk of recurrence will be determined for all SCOPE patients eligible for PQ treatment and non-SCOPE patients (and if applicable non-SCOPE PSM selected patients) administered PQ treatment. Multivariable Cox proportional hazards regression will be used to compare the SCOPE and non-SCOPE patients in the post-implementation period, controlling for calendar time at first visit (month) and seasonality (captured using splines) and a shared frailty of health facility. The validity of the proportional hazards assumption will be assessed visually using Schoenfeld residuals and stratified analyses will be conducted where the assumption is violated.

In the scenario that there are no substiantial differences in the baseline characteristics between patients enrolled and not enrolled in SCOPE, propensity score matching will not be used for pseudo-randomisation and the Cox proportional hazards regression will adjust for age, sex and body weight, with shared frailty of health facility.

12 Quality Control Plans

Details of data cleaning and verification, including consistency and range checks, will be specified in a Data Management Plan.

12 References

1. Taylor WRJ, Thriemer K, von Seidlein L, Yuentrakul P, Assawariyathipat T, Assefa A, et al (2019). Short-course primaquine for the radical cure of Plasmodium vivax malaria: a multicentre, randomised, placebo-controlled non-inferiority trial. The Lancet **394**(10202):929-38
2. Fadilah I, Commons RJ, Chau NH, Chu CS, Day NPJ, Koh G, et al (2024). Methaemoglobin as a surrogate marker of primaquine antihypnozoite activity in Plasmodium vivax malaria: A systematic review and individual patient data meta-analysis. PLoS Med **21**(9):e1004411
3. Barros AJD, Hirakata VN (2003). Alternatives for logistic regression in cross-sectional studies: an empirical comparison of models that directly estimate the prevalence ratio. BMC Med Res Methodol **3:** 21.
4. Bland JM, Altman DG (1995). Comparing methods of measurement: why plotting difference against standard method is misleading. The Lancet **346**(8982):1085-7.
5. Bernal, J.L., S. Cummins, and A. Gasparrini (2017). Interrupted time series regression for the evaluation of public health interventions: a tutorial. Int J Epidemiol **46**(1): p. 348-355.
6. Bazo-Alvarez, J. C., T. P. Morris, T. M. Pham, J. R. Carpenter and I. Petersen (2020). Handling Missing Values in Interrupted Time Series Analysis of Longitudinal Individual-Level Data. Clin Epidemiol **12**: 1045-1057.
7. Turner, S. L., A. Karahalios, A. B. Forbes, M. Taljaard, J. M. Grimshaw, E. Korevaar, A. C. Cheng, L. Bero and J. E. McKenzie (2021). Creating effective interrupted time series graphs: Review and recommendations. Research Synthesis Methods **12**(1): 106-117.
8. Turner S.L., et al. (2021). Evaluation of statistical methods used in the analysis of interrupted time series studies: a simulation study. BMC Med Res Methodol **21**(1): p. 181.

Appendix 1

VARIABLES IN THE DATA SET

Variable in data set	Units	Variable label
**Baseline/Enrolment CRF**		
scopeid	N/A	SCOPE Study ID
country	N/A	Country
staffid	N/A	Staff Identification Number
studysite	N/A	Study Site
visitdat	N/A	Visit Date
mcard	N/A	Indonesia: Malaria Card Number<br>PNG: Malaria ID
initials	N/A	Patient Initials
sex	N/A	Sex
age	years	Age (years)
agemonths	months	Age (months)
weight	kg	Weight
pregnant	N/A	Pregnant
lactating	N/A	Indonesia: Lactating < 6m<br>PNG: Lactating (infant younger than 12 months)
village01	NA	Village of residence
village02	NA	Village of residence
ethnic	N/A	Indonesia: Ethnic Group<br>PNG: Ethnicity
ethnichigh	N/A	Highland Papuan
ethniclow	N/A	Lowland Papuan
ethnicoth	N/A	Other Papuan (specify)
ethnicnon	N/A	Non-Papuan (specify)
diagnostic	N/A	Diagnostic
ringform	N/A	Ringform
gametocyte	N/A	Gametocyte
diagnosis_ms	N/A	Diagnosis (Microscopy)
diagnosis_rdt	N/A	Indonesia: Diagnosis (RDT)<br>PNG: RDT test result
g6pdtester	N/A	Indonesia: Staff ID (G6PD Tester)<br>PNG: ID of staff conducting G6PD test
g6pd	U/g Hb	G6PD
g6pdoor	N/A	G6PD Out of Measuring Range
hbhc	U/g Hb	Hb (Hemocue)
hbhcoor	N/A	Hb Hemocue Out of Measuring Range
hb	g/dL	Hb
hboor	N/A	Hb Out of Measuring Range
g6pdcat	N/A	G6PD Category
prescriber	N/A	Indonesia: Staff ID (Prescriber)<br>PNG: ID number of prescriber
pqduration	N/A	PQ Treatment Duration
pq	Tablets	PQ (total tablets)
pqobs	N/A	Indonesia: 1st dose PQ observed?<br>PNG: First dose PQ taken at facility
pqfood	N/A	PQ taken with food?
pqreas	N/A	Indonesia: Reason 1st dose PQ not observed<br>PNG: Reason 1st dose PQ not taken
pqreasoth	N/A	Indonesia: Other reason 1st dose PQ not observed<br>PNG: Other reason 1st dose PQ not taken
dhp	DHP (total tablets)	DHP (total tablets)
dhpobs	1st dose DHP observed?	1st dose DHP observed?
dhpreas	Reason 1st dose DHP not observed	Reason 1st dose DHP not observed
dhpreasoth	Other reason 1st dose DHP not observed	Other reason 1st dose DHP not observed
al	AL (total tablets)	AL (total tablets)
txother	Other Treatment	Other Treatment
comments	Comments	Comments
**Level 2 Review CRF**		
scopeid	*N/A*	*SCOPE Study ID*
studysite	*N/A*	*Study Site*
l2_reviewdat	N/A	Date of Review
l2_reviewday	N/A	Day of Review
l2_reviewdayoth	N/A	Day of Review (other)
l2_location	N/A	Location of Assessment
l2_locationoth	N/A	Location of Assessment (Other)

l2_reviewer	N/A	Indonesia: Staff Identification Number<br>PNG: Staff performing Level 2 Review
initials	*N/A*	*Patient Initials*
mcard	*N/A*	*Indonesia: Malaria Card Number*<br>*PNG: Malaria ID*
sex	*N/A*	*Sex*
age	*years*	*Age (years)*
agemonths	*months*	*Age (months)*
pqduration	*N/A*	*Initial Treatment*
l2_tabs	N/A	Yesterday, did you take your malaria tablets?
l2_tabsfood	N/A	If Yes, did you take them with food?
l2_temp	°C	Temperature
l2_pulse	beats per minute	Pulse
l2_resp	breaths per minute	Respiratory Rate
l2_abdosev	N/A	Abdominal pain - Severity
l2_abdodat	N/A	Abdominal pain - Date of Onset
l2_nauseasev	N/A	Nausea - Severity
l2_nauseadat	N/A	Nausea - Date of Onset
l2_eatsev	N/A	Unable to eat - Severity
l2_eatdat	N/A	Unable to eat - Date of Onset
l2_vomitsev	N/A	Vomiting - Severity
l2_vomitdat	N/A	Vomiting - Date of Onset
l2_backpainsev	N/A	Back pain - Severity
l2_backpaindat	N/A	Back pain - Date of Onset
l2_breathsev	N/A	Breathlessness - Severity
l2_breathdat	N/A	Breathlessness - Date of Onset
l2_dizzysev	N/A	Dizziness - Severity
l2_dizzydat	N/A	Dizziness - Date of Onset
l2_fatigue	N/A	Fatigue
l2_fatiguedat	N/A	Fatigue - Date of Onset
l2_vomit2	N/A	Vomiting > 2 x per day
l2_vomit2dat	N/A	Vomiting > 2 x per day - Date of Onset
l2_jaund	N/A	Jaundice
l2_jaunddat	N/A	Jaundice - Date of Onset
l2_blue	N/A	Blue lips
l2_bluedat	N/A	Blue lips - Date of Onset
l2_dkurine	N/A	Dark urine
l2_dkurinedat	N/A	Dark urine - Date of Onset
l2_othsymp	N/A	Other symptom(s)
l2_othsympdat	N/A	Other symptom(s) - Date of Onset
g6pd	*U/g Hb*	*G6PD*
g6pdoor	*N/A*	*G6PD Out of Measuring Range*
hb	*g/dL*	*Hb*
hboor	*N/A*	*Hb Out of Measuring Range*
l2_g6pdtester	N/A	Indonesia: Staff ID (G6PD Tester)<br>PNG: ID of staff conducting G6PD test
l2_hbhcdat	N/A	Hemocue Date (today)
l2_hbhc	g/dL	Hemocue Hb (today)
l2_hbhcoor	N/A	Hemocue Hb Out of Measuring Range
l2_chghbhc	g/dL	Hemocue Change in Hb (Calculated)
l2_changehbhc	g/dL	Hemocue Change in Hb
l2_g6pddat	N/A	Biosensor Date (today)
l2_g6pd	U/g Hb	G6PD (today)
l2_g6pdoor	N/A	G6PD Out of Measuring Range
l2_hb	g/dL	Hb (today)
l2_hboor	N/A	Hb Out of Measuring Range
l2_changehb	g/dL	Change in Hb (Calculated)
l2_chghb	g/dL	Change in Hb
l2_methbdat	N/A	MetHb Date
l2_methb	%	MetHb %
l2_needref	N/A	Need for Referral?
l2_pq	N/A	Primaquine
l2_pqdose	mg	PQ Daily Dose
l2_pqday	N/A	Frequency of dosing
l2_pqduration	days	PQ Duration
l2_refl3	N/A	Referred for Level 3 Review
l2_refl3dat	N/A	Level 3 Referral Date
l2_refl3tim	N/A	Level 3 Referral Time
l2_l3conf	N/A	Level 3 Confirmation
l2_l3confdat	N/A	Level 3 Confirmation Date
l2_l3conftim	N/A	Level 3 Confirmation Time
l2_whatsapp	N/A	Whatsapp Group
l2_whatsappdat	N/A	Whatsapp Group Date
l2_education	N/A	Education Given
l2_comments	N/A	Comments
**Level 3 Review CRF**		

scopeid	*SCOPE Study ID*	*N/A*
studysite	*Study Site*	*N/A*
l3_location	Location	N/A
l3_locationoth	Location (Other)	N/A
l3_reptype	Report Type	N/A
l3_fupno	Follow-up Number	N/A
l3_reviewdat	Date of Level 3 Review	N/A
l3_reviewtim	Time of Level 3 Review	N/A
l3_reviewer	Indonesia: Staff Identification Number<br>PNG: Staff performing Level 3 Review	N/A
initials	*Patient Initials*	*N/A*
mcard	*Indonesia: Malaria Card Number*<br>*PNG: Malaria ID*	*N/A*
sex	*Sex*	*N/A*
age	*Age (years)*	*years*
agemonths	*Age (months)*	*months*
pqduration	*Initial Treatment*	*N/A*
l3_referdat	Date referred	N/A
l3_temp	Temperature	°C
l3_pulse	Pulse	beats per minute
l3_resp	Respiratory Rate	breaths per minute
l3_abdosev	Abdominal pain - Severity	N/A
l3_abdodat	Abdominal pain - Date of Onset	N/A
l3_nauseasev	Nausea - Severity	N/A
l3_nauseadat	Nausea - Date of Onset	N/A
l3_eatsev	Unable to eat - Severity	N/A
l3_eatdat	Unable to eat - Date of Onset	N/A
l3_vomitsev	Vomiting - Severity	N/A
l3_vomitdat	Vomiting - Date of Onset	N/A
l3_backpainsev	Back pain - Severity	N/A
l3_backpaindat	Back pain - Date of Onset	N/A
l3_breathsev	Breathlessness - Severity	N/A
l3_breathdat	Breathlessness - Date of Onset	N/A
l3_dizzysev	Dizziness - Severity	N/A
l3_dizzydat	Dizziness - Date of Onset	N/A
l3_fatiguesev	Fatigue - Severity	N/A
l3_fatiguedat	Fatigue - Date of Onset	N/A
l3_fever	Fever	N/A
l3_feverdat	Fever - Date of Onset	N/A
l3_pallor	Severe pallor	N/A
l3_pallordat	Severe pallor - Date of Onset	N/A
l3_jaund	Jaundice	N/A
l3_jaunddat	Jaundice - Date of Onset	N/A
l3_blue	Blue lips	N/A
l3_bluedat	Blue lips - Date of Onset	N/A
l3_dkurine	Dark urine	N/A
l3_dkurinedat	Dark urine - Date of Onset	N/A
l3_hillmen	Indonesia: Dark urine colour (Hillmen)<br>PNG: Hillmen Colour Chart #:	N/A
l3_othsymp	Other symptom(s)	N/A
l3_othsympdat	Other symptom(s) - Date of Onset	N/A
l3_narrativecp	Narrative	N/A
hb	*Hb*	*g/dL*
hboor	*Hb Out of Measuring Range*	*N/A*
l3_g6pdtester	Indonesia: Staff ID (G6PD Tester)<br>PNG: ID of staff conducting G6PD test	N/A
l3_hbhcdat	Hemocue Hb Date (Today)	N/A
l3_hbhc	Hemocue Hb (Today)	g/dL
l3_hbhcoor	Hemocue Hb Out of Measuring Hemocue Range	N/A
l3_hbhcmethod	Hemocue Hb Method (Today)	N/A
l3_nadhbhc	Hemocue Hb (Nadir) (Calculated)	g/dL
l3_nadhbhcoor	Hemocue Hb Out of Measuring Range (Nadir) (Calculated)	N/A
l3_hbhcmaxfall	Hemocue Max Fall Hb (Calculated)	g/dL
l3_hbhcpcfall	Hemocue Max % Fall Hb (Calculated)	%
l3_hbdat	Hb Date (Today)	N/A
l3_hb	Hb (Today)	g/dL
l3_hboor	Hb Out of Measuring Range	N/A
l3_hbmethod	Hb Method (Today)	N/A
l3_nadhb	Hb (Nadir) (Calculated)	g/dL
l3_nadhboor	Hb Out of Measuring Range (Nadir) (Calculated)	N/A
l3_hbmaxfall	Max Fall Hb (Calculated)	g/dL
l3_hbpcfall	Max % Fall Hb (Calculated)	%
g6pd	*G6PD*	*U/g Hb*
g6pdoor	*G6PD Out of Measuring Range*	*N/A*

l3_methbdat	MetHb Date	N/A
l3_methb	MetHb %	%
l3_maxgrade	Max Graded Symptom	N/A
l3_maxgradedat	Max Graded Symptom – Date of Onset	N/A
l3_outcome	AE Outcome	N/A
l3_aesihaem	AESI Haemolysis	N/A
l3_aesihaemdat	AESI Haemolysis – Date of Onset	N/A
l3_aesihaemflag	AESI Haemolysis – Flags	N/A
l3_aesigast	AESI Gastrointestinal	N/A
l3_aesigastdat	AESI Gastrointestinal – Date of Onset	N/A
l3_aesigastflag	AESI Gastrointestinal – Flags	N/A
l3_aesimethb	AESI MetHb	N/A
l3_aesimethbdat	AESI MetHb – Date of Onset	N/A
l3_aesimethbflag	AESI MetHb – Flags	N/A
l3_sae	SAE	N/A
l3_saetype	SAE Type	N/A
l3_saetypeoth	Other SAE Type	N/A
l3_pqchange	Changes to PQ	N/A
l3_pqdose	PQ Daily Dose	mg
l3_pqday	Frequency of dosing	N/A
l3_pqduration	PQ Duration	days
l3_narrativeaesi	Narrative for AESI	N/A
l3_review	Planned Review	N/A
l3_reviewrtn	Planned Review Routine	N/A
l3_reviewoth	Other follow-up Day	N/A
l3_reviewothdat	Other follow-up Date	N/A
l3_refhospdat	Referred to Hospital Date	N/A
l3_refhosptim	Referred to Hospital Time	N/A
l3_notify	Notification	N/A
l3_comments	Comments	N/A
**Serious Adverse Event CRF**		
scopeid	*SCOPE Study ID*	*N/A*
studysite	*Study Site*	*N/A*
sae_reviewdat	Date of Assessment	N/A
sae_reviewtim	Time of Assessment	N/A
sae_reptype	Report Type	N/A
sae_fupno	Follow-up Number	N/A
sae_location	Location of Assessment	N/A
initials	*Patient Initials*	*N/A*
mcard	*Indonesia: Malaria Card Number*<br>*PNG: Malaria ID*	*N/A*
sex	*Sex*	*N/A*
age	*Age (years)*	*years*
agemonths	*Age (months)*	*months*
weight	*Weight*	*kg*
country	*Country*	*N/A*
sae_pqdoses	Number of PQ doses taken before AE detected	N/A
sae_pqfood	Did the patient take their last PQ tablets with food?	N/A
sae_cm	Concomitant Medication	N/A
sae_cmname1	Concomitant Medication 1 Name	N/A
sae_cmstdat1	Concomitant Medication 1 Start Date	N/A
sae_cmenddat1	Concomitant Medication 1 Stop Date	N/A
sae_cmstdose1	Concomitant Medication 1 Dose	N/A
sae_cmfreq1	Concomitant Medication 1 Frequency	N/A
sae_cmname2	Concomitant Medication 1 Name	N/A
sae_cmstdat2	Concomitant Medication 1 Start Date	N/A
sae_cmenddat2	Concomitant Medication 1 Stop Date	N/A
sae_cmstdose2	Concomitant Medication 1 Dose	N/A
sae_cmfreq2	Concomitant Medication 1 Frequency	N/A
sae_cmname3	Concomitant Medication 1 Name	N/A
sae_cmstdat3	Concomitant Medication 1 Start Date	N/A
sae_cmenddat3	Concomitant Medication 1 Stop Date	N/A
sae_cmstdose3	Concomitant Medication 1 Dose	N/A
sae_cmfreq3	Concomitant Medication 1 Frequency	N/A
sae_cmname4	Concomitant Medication 1 Name	N/A
sae_cmstdat4	Concomitant Medication 1 Start Date	N/A
sae_cmenddat4	Concomitant Medication 1 Stop Date	N/A
sae_cmstdose4	Concomitant Medication 1 Dose	N/A
sae_cmfreq4	Concomitant Medication 1 Frequency	N/A
sae_cmname5	Concomitant Medication 1 Name	N/A
sae_cmstdat5	Concomitant Medication 1 Start Date	N/A
sae_cmenddat5	Concomitant Medication 1 Stop Date	N/A
sae_cmstdose5	Concomitant Medication 1 Dose	N/A
sae_cmfreq5	Concomitant Medication 1 Frequency	N/A
sae_cmname6	Concomitant Medication 1 Name	N/A

sae_cmstdat6	Concomitant Medication 1 Start Date	N/A
sae_cmenddat6	Concomitant Medication 1 Stop Date	N/A
sae_cmstdose6	Concomitant Medication 1 Dose	N/A
sae_cmfreq6	Concomitant Medication 1 Frequency	N/A
sae_maindx	AE Main Diagnosis	N/A
sae_stdat	Date AE Started	N/A
sae_serdat	Date AE met Serious Criteria	N/A
sae_detdat	Date SAE detected	N/A
sae_hospadmdat	Date of Hospitalisation	N/A
sae_hospna	Hospitalisation Not Applicable	N/A
sae_hospdisdat	Date of Discharge	N/A
sae_hosp	Still in Hospital	
sae_narrativecp	Narrative	
sae_med	Relevant Medical History	N/A
sae_medname1	Medical Condition 1 Name	N/A
sae_medstdat1	Medical Condition 1 Start Date	N/A
sae_medenddat1	Medical Condition 1 End Date	N/A
sae_medong1	Medical Condition 1 Ongoing	N/A
sae_medname2	Medical Condition 2 Name	N/A
sae_medstdat2	Medical Condition 2 Start Date	N/A
sae_medenddat2	Medical Condition 2 End Date	N/A
sae_medong2	Medical Condition 2 Ongoing	N/A
sae_medname3	Medical Condition 3 Name	N/A
sae_medstdat3	Medical Condition 3 Start Date	N/A
sae_medenddat3	Medical Condition 3 End Date	N/A
sae_medong3	Medical Condition 3 Ongoing	N/A
sae_medname4	Medical Condition 4 Name	N/A
sae_medstdat4	Medical Condition 4 Start Date	N/A
sae_medenddat4	Medical Condition 4 End Date	N/A
sae_medong4	Medical Condition 4 Ongoing	N/A
sae_hbhcdetdat	Hemocue Hb Date (At time of Detection)	N/A
sae_hbhcdet	Hemocue Hb (At time of Detection)	g/dL
sae_hbhcdetoor	Hemocue Hb Out of Measuring Range	N/A
sae_hbhcdetmeth	Hemocue Hb Method (At time of Detection)	N/A
sae_hbhcnaddat	Hemocue Hb Date (Nadir)	N/A
sae_hbhcnad	Hemocue Hb (Nadir)	g/dL
sae_hbhcnadmeth	Hemocue Hb Method (Nadir)	N/A
sae_g6pdtester	Indonesia: Staff ID (G6PD Tester)<br>PNG: ID of staff conducting G6PD test	N/A
sae_hbhc	Hemocue Hb (Current)	g/dL
sae_hbhcdat	Hemocue Hb Date (Current)	N/A
sae_hbhcmeth	Hemocue Hb Method (Current)	N/A
sae_hbhcoor	Hemocue Hb Out of Measuring Range	N/A
sae_hbhcmaxfall	Hemocue Max Fall Hb	g/dL
sae_hbhcpcfall	Hemocue Max % Fall Hb	%
sae_hbdetdat	Hb Date (At time of Detection)	N/A
sae_hbdet	Hb (At time of Detection)	g/dL
sae_hboor	Hb Out of Measuring Range	N/A
sae_hbdetmeth	Hb Method (At time of Detection)	N/A
sae_hbnaddat	Hb Date (Nadir)	N/A
sae_hbnad	Hb (Nadir)	g/dL
sae_hbnadmeth	Hb Method (Nadir)	N/A
sae_hbcur	Hb (Current)	g/dL
sae_hbcurdat	Hb Date (Current)	N/A
sae_hbcurmeth	Hb Method (Current)	N/A
sae_hbmaxfall	Max Fall Hb	g/dL
sae_hbpcfall	Max % Fall Hb	%
sae_curg6pddat	G6PD Date	N/A
sae_curg6pd	G6PD Result	U/g Hb
sae_g6pdoor	G6PD (Out of Measuring Range)	N/A
sae_g6pdcat	G6PD Category	N/A
sae_g6pdgen	G6PD Genotyping	N/A
sae_g6pdvariant	Genotyping Variant	N/A
sae_wbcdat	WBC Date	N/A
sae_wbc	WBC Result	$x10^3/\mu L$
sae_pltdat	Plt Date	N/A
sae_plt	Plt Result	$x10^3/\mu L$
sae_methbdat	MetHb Date	N/A
sae_methb	MetHb Result	%
sae_nadat	Na Date	N/A
sae_na	Na Result	mmol/L
sae_kdat	K Date	N/A
sae_k	K Result	mmol/L
sae_ureadat	Urea Date	N/A
sae_urea	Urea Result	mg/dL
sae_creatdat	Creatinine Date	N/A
sae_creat	Creatinine Result	mg/dL

sae_totbilidat	Total Bili Date	N/A
sae_totbili	Total Bili Result	µmol/L
sae_unconjbilidat	Unconj Bili Date	N/A
sae_unconjbili	Unconj Bili Result	µmol/L
sae_alpdat	ALP Date	N/A
sae_alp	ALP Result	µmol/L
sae_altdat	ALT Date	N/A
sae_alt	ALT Result	µmol/L
sae_ldhdat	LDH Date	N/A
sae_ldh	LDH Result	µmol/L
sae_testoth	Other Results	N/A
sae_saetype	SAE Type	N/A
sae_organs	Organ Systems Involved	N/A
sae_relpq	Relationship (causality) to PQ	N/A
sae_iv	IV Fluids	N/A
sae_bltr	Blood Transfusion	N/A
sae_bltrunits	Blood Transfusion – Number of units	N/A
sae_bltrdat	Blood Transfusion – Date	N/A
sae_dial	Dialysis	N/A
sae_dialtype	Dialysis Type	N/A
sae_pqchange	Changes to PQ	N/A
sae_pqdat	PQ Date Restarted	N/A
sae_pqdose	PQ Daily Dose	mg
sae_pqday	Frequency of dosing	N/A
sae_pqduration	PQ Duration	days
sae_narrativecm	Narrative	N/A
sae_outcome	Outcome	N/A
sae_rrs	Recovered/Resolved with Sequelae – Specify	N/A
sae_dod	Date of Death	N/A
sae_cod	Cause of Death	N/A
sae_lastdat	AE Stopped – Last date AE was present	N/A
sae_notify	Notification	N/A
sae_comments	Comments	N/A

Appendix 2

LIST OF TABLES/FIGURES/LISTINGS

Number	Title
Table 1	Baseline characteristics of patients enrolled in Stage 1 safety and feasibility study by health facility site
Table 2	Baseline characteristics of patients enrolled in Stage 1 safety and feasibility study by treatment arm
Table 3	Number of events for patients enrolled in Stage 1 safety and feasibility study by health facility site
Table 4	Number of patients with at least one event enrolled in Stage 1 safety and feasibility study by health facility site
Table 5	Number of patients with at least one event enrolled in Stage 1 safety and feasibility study by treatment arm
Table 6	Baseline characteristics of individuals presenting to health facilities by pre-implementation and post-implementation (Stage 2) phases of study
Table 7	Estimates of the effect of the implementation package on the incidence of symptomatic *P. vivax* malaria episodes
Table 8	Baseline characteristics of individuals included in the cross-sectional surveys in the pre-implementation and post-implementation (Stage 2) phases of study
Table 9	Estimates of the effect of the implementation package on the prevalence of *P. vivax* parasitaemia and severe anaemia (haemoglobin < 7g/dl) in patients presenting with fever in the cross-sectional surveys in the pre-implementation and post-implementation (Stage 2) phases of study
Table 10	Descriptive statistics and 95% limits of agreement for haemoglobin measurements obtained by using Biosensor and HemoCue, stratified by day of measurement (day 0, 3, 7) and country
Table S1	Protocol deviations for patients enrolled in Stage 1 safety and feasibility study by health facility site
Table S2	Baseline characteristics of individuals presenting to health facilities by pre-implementation and post-implementation (Stage 2) phases of study for each health facility site
Table S3	Estimates of the effect of the implementation package on the incidence of symptomatic *P. vivax* malaria episodes – alternate seasonality specification (adjustment for rainy/dry season)
Figure 1	Study overview
Figure 2	Incidence of symptomatic *P. vivax* malaria episodes over time. The introduction of the implementation package at 6 months is indicated by the dashed red vertical line. The trend lines for the pre- and post-implementation stages are presented as solid blue lines, and the counterfactual to the post-implementation trend presented as a dashed blue line. *[Note: the example figure is given for a single health facility site]*
Figure 3	Bland–Altman plots comparing the measurements haemoglobin obtained using HemoCue and SD Biosensor.

Appendix 3 – Dummy tables and figures

Table 1. Baseline characteristics of patients enrolled in Stage 1 safety and feasibility study by health facility site

	**Overall**	**HF1**	**HF2**	**HF3**	**HF4**
	N=	N=	N=	N=	
**Age (mean and range)**					
<5yrs					
5-15					
>15yrs					
Unknown					
**Sex n (%)**					
Male					
Female					
Unknown					
**Malaria diagnosis n (%)**					
Pv mono infection (microscopy)					
Pv mixed infection (microscopy)					
Pv mono/mixed (RDT)					
Total Pv (mono or mixed)					
Unknown					
**G6PD value**					
Normal					
Intermediate All					
Intermediate Females					
Intermediate Males					
Deficient					
Unknown					
**Treatment**					
PQ7					
PQ14					
PQ8W					
None					
Unknown					

Table 2. Baseline characteristics of patients enrolled in Stage 1 safety and feasibility study by treatment arm

	**Overall**	**PQ7**	**PQ14**	**PQ8W**
	N=	N=	N=	N=
**Age (mean and range)**				
<5yrs				
5-15				
>15yrs				
**Sex n (%)**				
Male				
Female				
Unknown				
**Malaria diagnosis n (%)**				
Pv mono infection (microscopy)				
Pv mixed infection (microscopy)				
Pv mono/mixed (RDT)				
Total Pv (mono or mixed)				
Unknown				

Table 3. Number of events for patients enrolled in Stage 1 safety and feasibility study by health facility site

	**Overall**	**HF1**	**HF2**	**HF3**	**HF4**
**Total Enrolled N**					
Flagged Day 3-5 N(%) *					
Flagged Day 6-10 N(%) *					
Other Day Flagged N(%) *					
**AEs n(%)**					
Total Grade 3					
Total Grade 4					
**Total**					
**AESI**					
**Haemolysis**					
AESI (SAE)					
AESI (NonSAE)					
**Total**					
**Gastrointestinal**					
AESI (SAE)					
AESI (NonSAE)					
**Total**					
**Methaemoglobinaemia**					
AESI (SAE)					
AESI (NonSAE)					
**Total**					
**SAEs**					
SAE – definitely related					
SAE – probably related					
SAE – possibly related					
SAE – unlikely related					
SAE – not related					
**Total**					
Related SAE which is also an AESI					
Related SAE Other					

Table 4. Number of patients with at least one event who are enrolled in Stage 1 safety and feasibility study by health facility site

	**Overall**	**HF1**	**HF2**	**HF3**	**HF4**
**Total Enrolled N**					
Flagged Day 3-5 N(%) *					
Flagged Day 6-10 N(%) *					
Other Day Flagged N(%) *					
**Aes n(%)**					
Total Grade 3					
Total Grade 4					
**Total**					
**AESI**					
**Haemolysis**					
AESI (SAE)					
AESI (NonSAE)					
**Total**					
**Gastrointestinal**					
AESI (SAE)					
AESI (NonSAE)					
**Total**					
**Methaemoglobinaemia**					
AESI (SAE)					
AESI (NonSAE)					
**Total**					
**SAEs**					
SAE – definitely related					
SAE – probably related					
SAE – possibly related					
SAE – unlikely related					
SAE – not related					
**Total**					
Related SAE which is also an AESI					
Related SAE Other					

Table 5. Number of patients with at least one event enrolled in Stage 1 safety and feasibility study by treatment arm

	Overall	PQ7	PQ14	PQ8W
**Total Enrolled N**				
Flagged Day 3-5 N(%) *				
Flagged Day 6-10 N(%) *				
Other Day Flagged N(%) *				
**Aes n(%)**				
Total Grade 3				
Total Grade 4				
**Total**				
**AESI**				
**Haemolysis**				
AESI (SAE)				
AESI (NonSAE)				
**Total**				
**Gastrointestinal**				
AESI (SAE)				
AESI (NonSAE)				
**Total**				
**Methaemoglobinaemia**				
AESI (SAE)				
AESI (NonSAE)				
**Total**				
**SAEs**				
SAE – definitely related				
SAE – probably related				
SAE – possibly related				
SAE – unlikely related				
SAE – not related				
**Total**				
Related SAE which is also an AESI				
Related SAE Other				

Table 6. Baseline characteristics of individuals presenting to health facilities by pre-implementation and post-implementation (Stage 2) phases of study

	Pre-implementation	Post-implementation (Stage 2)
**Number of individuals**	N	N
**Number of individuals per health facility site**	x (x, x) [min, max]	x (x, x) [min, max]
**Age, years (mean and range)**		
<5yrs		
5-15		
>15yrs		
Unknown		
**Sex n (%)**		
Male		
Female		
Unknown		
**Malaria diagnosis n (%)**		
Pv mono infection (microscopy)		
Pv mixed infection (microscopy)		
Pv mono/mixed (RDT)		
Total Pv (mono or mixed)		
Unknown		
**G6PD value**		
Normal		
Intermediate All		
Intermediate Females		
Intermediate Males		
Deficient		
Unknown		
**Treatment**		
PQ7		
PQ14		
PQ8W		
None		
Unknown		

N = Total number; x (x, x) = median (lower quartile, upper quartile).

Table 7. Estimates of the effect of the implementation package on the incidence of symptomatic *P. vivax* malaria episodes

	Pre-implementation	Post-implementation	Post- versus pre-implementation
**Effect of intervention 6 months after date of implementation**	Est of IR (lb, ub)	Est of IR (lb, ub)	Est of IRR (lb, ub)
**Effect of intervention on slope after date of implementation**	Est of change in IR per month (lb, ub)	Est of change in IR per month (lb, ub)	Est of slope change (lb, ub)

Est (lb, ub) = Estimate (lower bound of 95% CI, upper bound of 95% CI), IR – Incidence Rate, IRR – Incidence Rate Ratio

Table 8. Baseline characteristics of individuals included in the cross-sectional surveys in the pre-implementation and post-implementation (Stage 2) phases of study

	Pre-implementation survey	Post-implementation (Stage 2) survey
**Number of individuals**	N	N
**Number of individuals per site**	x (x, x) [min, max]	x (x, x) [min, max]
**Age, years (mean and range)**		
<5yrs		
5-15		
>15yrs		
Unknown		
**Sex n (%)**		
Male		
Female		
Unknown		
**Malaria diagnostic method n (%)**		
Film		
RDT		
Both		
**Malaria diagnosis n (%)**		
Pv		
Pf		
Po		
Pm		
Mixed species		
Unknown		
**Parasitaemia (geometric mean and range)**		
Pv		
Pf		
Po		
Pm		
Mixed species		
**Gametocytaemia n (%)**		
Pv		
Pf		
Po		
Pm		
Mixed species		
**G6PD value**		
Normal		
Intermediate All		
Intermediate Females		
Intermediate Males		
Deficient		
Unknown		
**Haemoglobin (median, IQR, range)**		

**Anaemia <7g/dL (n (%)**		

Table 9. Estimates of the effect of the implementation package on the prevalence of *P. vivax* parasitaemia and severe anaemia (haemoglobin < 7g/dl) in patients presenting with fever in the cross-sectional surveys in the pre-implementation and post-implementation (Stage 2) phases of study

	**Pre-implementation**	**Post-implementation**	**Post- versus pre-implementation**
***P. vivax* parasitaemia**	Est of P (lb, ub)	Est of P (lb, ub)	Est of PR (lb, ub)
**Severe anaemia**	Est of P (lb, ub)	Est of P (lb, ub)	Est of PR (lb, ub)

Est (lb, ub) = Estimate (lower bound of 95% CI, upper bound of 95% CI), P – Prevalence, IRR – Prevalence Ratio

Table 10. Descriptive statistics and 95% limits of agreement for haemoglobin measurements obtained by using Biosensor and HemoCue, stratified by day of measurement (day 0, 3, 7) and country

	HemoCue haemoglobin (g/dL)	SD Biosensor haemoglobin (g/dL)	Difference between two measurements (g/dL)	95% limits of agreement (g/dL)
	Mean (SD); N	Mean (SD); N	Mean (SD) [95% CI]	
**Indonesia**				
Day 0				
Day 3				
Day 7				
**PNG**				
Day 0				
Day 3				
Day 7				

Table S1. Protocol deviations for patients enrolled in Stage 1 safety and feasibility study by site

	**Overall**	**HF1**	**HF2**	**HF3**	**HF4**
	N=	N=	N=	N=	N=
**PRESCRIBING DEVIATIONS**					
**Pregnancy n (% of all female patients)**					
Treated with PQ					
**Breast Feeding infants (<6 months Indonesia, <12 months PNG) n (% of all female patients)**					
Treated with PQ					
**Age <1year (PNG), <6 months (Indonesia) n (% of all enrolled pts)**					
Treated with PQ					
**Patients with Pf (Mono) n (% of all enrolled pts)**					
Treated with PQ					
**G6PD normal >70% N(% of all enrolled pts)**					
Treated with PQ14 n(%)					
Treated with PQ8W n(%)					
**G6PD Intermediate 30-70% N(% of all enrolled pts)**					
Treated with PQ7 n(%)					
Treated with PQ8W n(%)					
**G6PD Deficient <30% N(% of all enrolled pts)**					
Treated with PQ7 n(%)					
Treated with PQ14 n(%)					
**DOSING DEVIATIONS**					
**PQ7 n(%)**					
daily dose > 1.3mg/kg					
daily dose < 0.5mg/kg					
**PQ14 n(%)**					
daily dose > 0.83mg/kg					
daily dose < 0.25mg/kg					
**PQ8W n(%)**					
daily dose > 1.25mg/kg					
daily dose < 0.38mg/kg					
**FOLLOW UP DEVIATIONS**					
Proportion of Patients without Day3 Review (Day 3-5) N(% **of all enrolled pts**)					
Proportion of Patients without Day7 Review (Day 6-10) N(% **of all enrolled pts**)					

Table S1a. Protocol deviations for patients enrolled in Stage 2 study by site

	Overall	HF1	HF2	HF3	HF4	HF5	HF6	HF7	HF8	HF9	HF10
	N=	N=	N=	N=	N=	N=	N=	N=	N=	N=	N=
**PRESCRIBING DEVIATIONS**											
**Pregnancy n (% of all female patients)**											
Treated with PQ											
**Breast Feeding infants (<6 months Indonesia, <12 months PNG) n (% of all female patients)**											
Treated with PQ											
**Age <1year (PNG), <6 months (Indonesia) n (% of all enrolled pts)**											
Treated with PQ											
**Patients with Pf (Mono) n (% of all enrolled pts)**											
Treated with PQ											
**G6PD normal >70% N(% of all enrolled pts)**											
Treated with PQ14 n(%)											
Treated with PQ8W n(%)											
**G6PD Intermediate 30-70% N(% of all enrolled pts)**											
Treated with PQ7 n(%)											
Treated with PQ8W n(%)											
**G6PD Deficient <30% N(% of all enrolled pts)**											

Treated with PQ7 n(%)											
Treated with PQ14 n(%)											
**DOSING DEVIATIONS**											
**PQ7 n(%)**											
daily dose > 1.3mg/kg											
daily dose < 0.5mg/kg											
**PQ14 n(%)**											
daily dose > 0.83mg/kg											
daily dose < 0.25mg/kg											
**PQ8W n(%)**											
daily dose > 1.25mg/kg											
daily dose < 0.38mg/kg											
**FOLLOW UP DEVIATIONS**											
Proportion of Patients without Day3 Review (Day 3-5) N(% **of all enrolled pts**)											
Proportion of Patients without Day7 Review (Day 6-10) N(% **of all enrolled pts**)											

Table S2. Baseline characteristics of individuals presenting to health facilities by pre-implementation and post-implementation (Stage 2) phases of study for each health facility site

Example table give for 2 health facility sites from PNG

	**PNG health facility site 1**		**PNG health facility site 2**	
	Pre-implementation	Post-implementation (Stage 2)	Pre-implementation	Post-implementation (Stage 2)
**Number of individuals**	N	N	N	N
**Number of individuals per**	x (x, x) [min, max]	x (x, x) [min, max]	x (x, x) [min, max]	x (x, x) [min, max]

**health facility site**				
**Age, years (mean and range)**				
<5yrs				
5-15				
>15yrs				
Unknown				
**Sex n (%)**				
Male				
Female				
Unknown				
**Malaria diagnosis n (%)**				
Pv mono infection (microscopy)				
Pv mixed infection (microscopy)				
Pv mono/mixed (RDT)				
Total Pv (mono or mixed)				
Unknown				
**G6PD value**				
Normal				
Intermediate All				
Intermediate Females				
Intermediate Males				
Deficient				
Unknown				
**Treatment**				
PQ7				
PQ14				
PQ8W				
None				
Unknown				

Table S3. Estimates of the effect of the implementation package on the incidence of symptomatic *P. vivax* malaria episodes (adjusted for seasonality – rainy / dry season)

	**Pre-implementation**	**Post-implementation**	**Post- versus pre-implementation**
**Effect of intervention 6 months after date of implementation**	Est of IR (lb, ub)	Est of IR (lb, ub)	Est of IRR (lb, ub)
**Effect of intervention on slope after date of implementation**	Est of change in IR per month (lb, ub)	Est of change in IR per month (lb, ub)	Est of slope change (lb, ub)

Est (lb, ub) = Estimate (lower bound of 95% CI, upper bound of 95% CI), IR – Incidence Rate, IRR – Incidence Rate Ratio

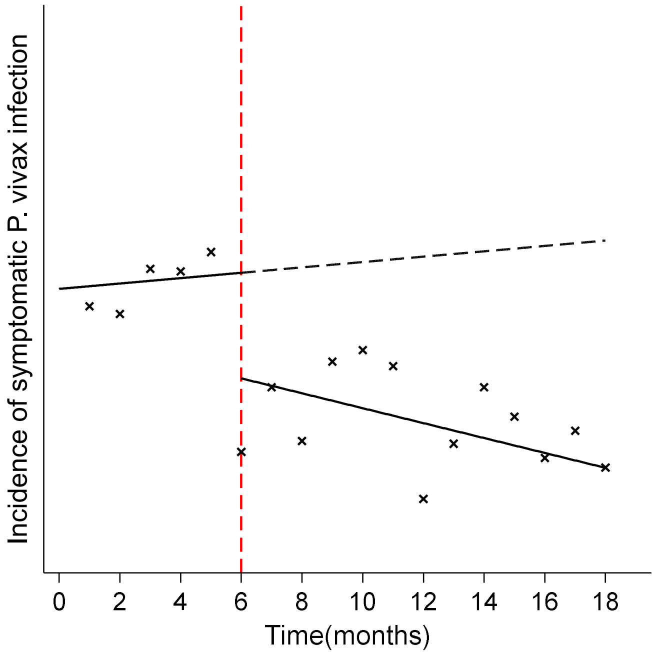

Figure 2. Incidence of symptomatic *P. vivax* malaria episodes over time. The introduction of the implementation package occurs at 6 months (dashed red vertical line). The trend lines for the pre- and post-implementation stages are presented as solid black lines, and the counterfactual to the post-implementation trend presented as a dashed black line.

Appendix 4 – Implementation definitions for S2.SO15

PRIMARY ANALYSIS

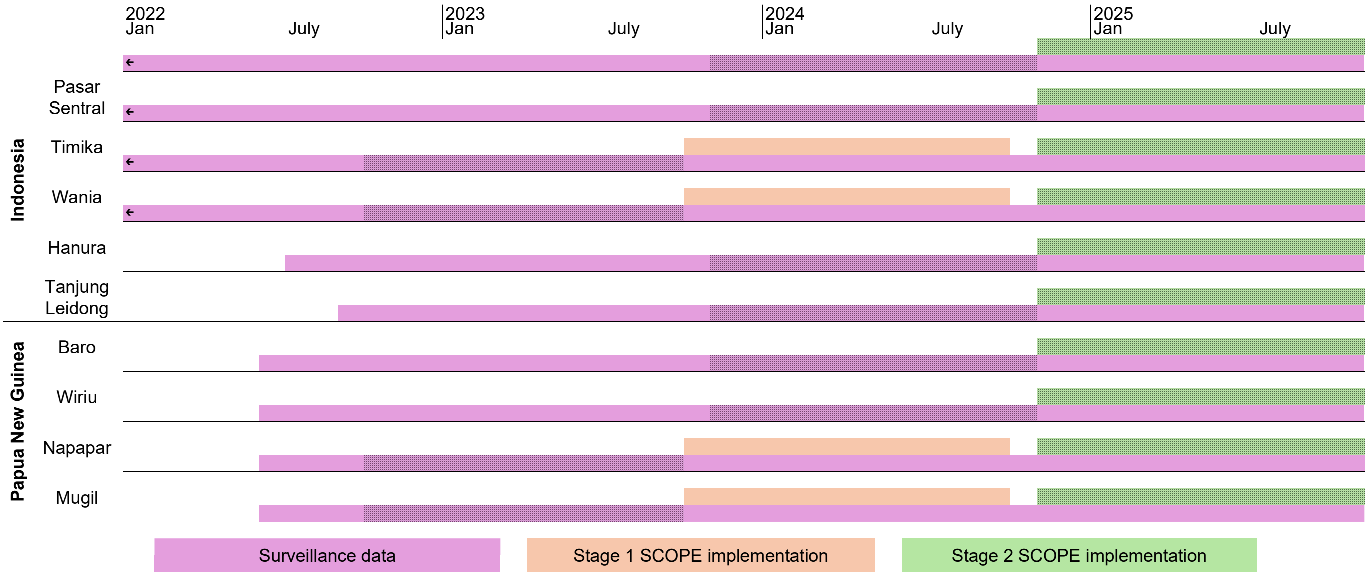

Figure 1: Pre- and post-implementation periods for S2.SO15 displayed by areas with shading. This includes stage 2 implementation only within the post-implementation period as seen by areas of green with shading overlay. The pre-implementation period for each health facility is shown by the shaded pink area.

SENSITIVITY ANALYSIS - Pools Stage 1 and Stage 2 and includes the one month downtime in between

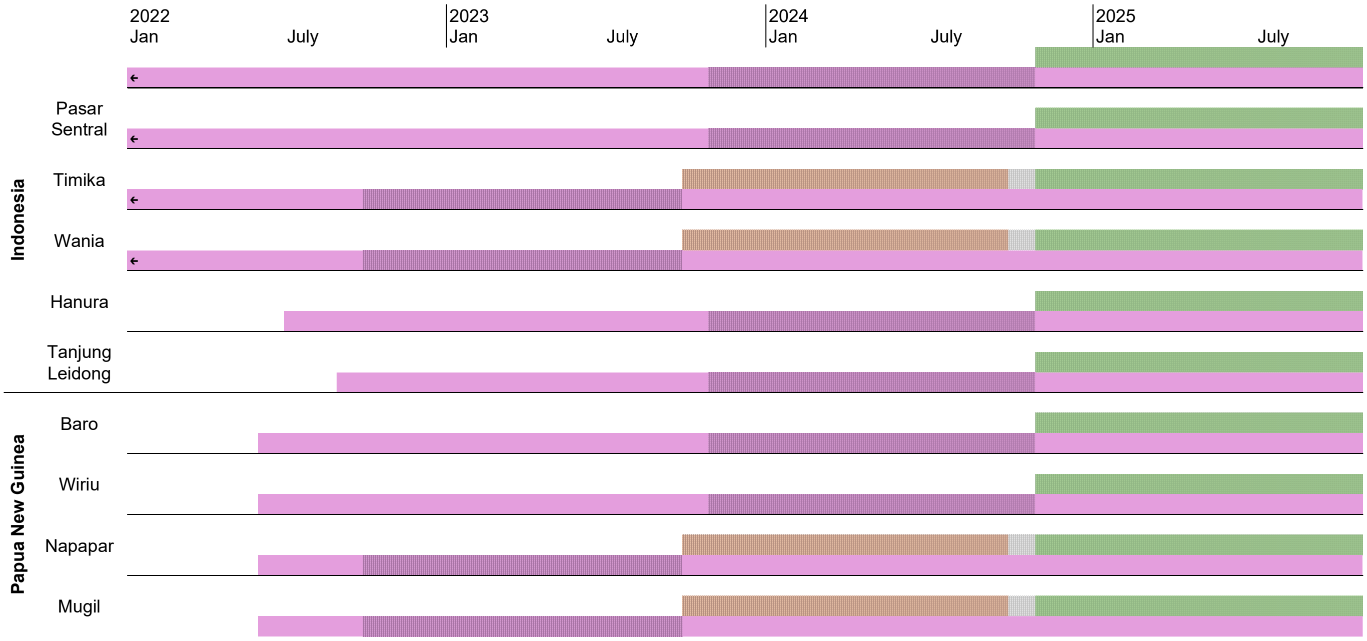

Figure 2: Pre- and post-implementation periods for S2.SO15 sensitivity analysis displayed by areas with shading. This includes stage 1 & 2 implementation within the post-implementation period as seen with areas of orange and green with shading overlay. The pre-implementation period for each health facility is shown by the shaded pink area.
