# Supplementary material for: High-dose, short-course primaquine after point-of-care G6PD testing for the radical cure of Plasmodium vivax malaria: a safety study in Papua New Guinea and Indonesia
Source: Lancet Reg Health West Pac. 2026 Jun 11;71:101903. doi: 10.1016/j.lanwpc.2026.101903 (PMC13276568; doi:10.1016/j.lanwpc.2026.101903)
Supplement: Supplementary File 5 [file mmc5.pdf]

SCOPE SERIOUS ADVERSE EVENT FORM

Staff Identification Number: FC/JA Study Site: Timika SCOPE ID: 1102

Summary of findings of the Safety Review Team

This documents outlines the key facts of the events associated with SAE _1102, the subsequent discussions of the SRT, and a review of the literature.

Description of the event

SCOPE participant 1102, a 13-year-old female of Moni ethnicity from Timika, Papua, was reported to have become acutely unwell within hours of her first dose of primaquine (17-Feb-24). Her family had taken her to the Mimika District hospital where she had been pronounced dead. We received the news from the family over the phone when the clinic staff were arranging the Day 3 review.

On enrolment, the patient had uncomplicated malaria without signs or symptoms of concern. She had been able to carry out normal day-to-day activities. Her G6PD test was normal (7.7 U/g Hb = >70% activity) and her haemoglobin was 10.2 g/dL (Biosensor). She was allocated to the high daily dose (1mg/kg/day) primaquine regimen (PQ7). She took the first doses of dihydroartemisinin-piperaquine (DP) and primaquine at 10:48am, supervised by the study team. The patient remained in the clinic for post-medication supervision and to collect other prescribed medications (DP, primaquine (0.99 mg/kg/day) and domperidone (10mg tds for nausea) from the clinic pharmacy. She did not take domperidone at the clinic prior to departure. The study team last saw her just before she went home, 45-60 minutes after the first antimalarial dose. She appeared well at the time of leaving the clinic. The only information regarding her symptoms before death were that she reported dizziness/headache. She was pronounced dead at the district hospital at 06:30pm on the same day.

Further history from the family is not possible due to local cultural sensitivities.

A recheck of her blood film confirmed monoinfection with *P. vivax* with a parasitaemia of 14,400 μl^{-1} (moderate parasitaemia).
A manual count of her blood film estimated her white cell count to be 4,467 per mm^3. The white cells were predominantly monocytes and there was no neutrophilia.

Further details are available in the SEA Report < SCOPE_1102_SAE_id_20240220.pdf>

Immediate Action - Tuesday 20th February 2024

All further enrolment in the SCOPE study was ceased, pending further review.

SCOPE SERIOUS ADVERSE EVENT FORM

Staff Identification Number: FC/JA Study Site: Timika SCOPE ID: 1102

The Safety Review Team (SRT) Convened on Thursday 22nd February 2024.

Minutes are attached < SRT meeting 22Feb2024_draft minutes>

The SRT stated sincere condolecences to the patient's family.

The review of data revealed notable lack of detail in the final hours before death. Further gathering of information from the family was not regarded as culturally appropriate or possible. Written informed consent for the study was provided by the guardian (sister), who signed the consent form, and by the patient, who signed the assent form.

The SRT regarded that the timing and nature of the event were not in keeping with known serious adverse events associated with primaquine (notably severe haemolysis, gastrointestinal intolerance or methaemoglobinaemia).

Possible causes of death considered included:

- acute cardia arrhythmia
- acute cerebral haemorrhage
- anaphylaxis
- acute sepsis
- hypoglycaemia

Anaphylaxis, acute sepsis and hypoglycaemia, were considered unlikely.
The differential between cardiac arrhythmia and cerebral haemorrhage would depend on the final event, the former associated with torsades de point is usually rapid with few symptoms. The latter maybe more prolonged and associated with symptoms (such as headache and dizziness).

Whilst the contribution of primaquine to the death was thought to be unlikely, the SRT did consider the potential of high dose primaquine compounding QT prolongation in a patient with congenital long QT syndrome in the context of piperaquine and domperidone administration (both of which have known proarrhythmic potential). To explore this further a systematic review of the literature of primaquine was conducted and expert advice was sought from pharmacologists.

Notification of SAE to Ethics Boards

Menzies HREC was notified on the 23/02/2024 and University of Gadjah Mada Ethics on the 24/02/2024. The WHO Ethics Committee will be informed as part of the annual reporting in March 2024.

SCOPE SERIOUS ADVERSE EVENT FORM

Staff Identification Number: FC/JA Study Site: Timika SCOPE ID: 1102

Review of the cardiac proarrhythmic potential of primaquine

Proarrhythmic Potential of Primaquine, Piperaquine and Domperidone

Primaquine: The relationship between ECG QT prolongation and risk of lethal Torsades de Point is complex[1]. Primaquine, like many quinoline antimalarial drugs, affects cation channels in muscle. The Sanofi Primaquine labels reports potential cardiac arrhythmia and QT interval prolongation in overdose. [2] It's unclear where these cautionary data come from. The only hERG channel study we can find is that of Kim et al which reported an IC_{50} in HEK 293 cells of 21.5 µM [3]. Based on dose linearity, this concentration corresponds with an approximately 20-fold higher plasma concentration than the peak concentrations predicted following a 1mg/kg dose (1 µM), even taking into account the piperaquine-primaquine interaction [6].

Piperaquine: In comparison the IC_{50} for piperaquine in HEK 293 cells reported by Borsini et al was 87nmol/L which is about 10-fold higher than the predicted free peak plasma concentration with standard dosing[4].

Domperidone has been associated with sudden death. The domperidone IC_{50} in HEK 293 cells was reported as 57nmol/L[5]. This is only 3-8.5 times higher than the maximum plasma concentrations achieved with standard dosing. Metoclopramide is much safer (10X) in this respect.

Clinical Cardiotoxicity and Primaquine

A comprehensive literature review of patients with *P. vivax* malaria hospitalised after primaquine therapy (publications 1960-2023) revealed 7 reported deaths associated with primaquine[6]. Four deaths occurred in Sri Lanka in the 1960s[7] and three were from Brazil[89]. None of the cases were suggestive of cardiac arrhythmia.

Seven clinical studies have reported ECG findings following primaquine. None of the studies demonstrated QT prolongation associated with primaquine administration (See Appendix for details of the trials). The studies included a randomised clinical trial of high daily dose primaquine (1mg/kg/day) administered over 3.5 days[10]. There was no evidence of cardiotoxic interaction between primaquine or piperaquine[11].

---

[1] Chan et al PlosMed 2020; https://www.ncbi.nlm.nih.gov/pubmed/32134952
[2] https://products.sanofi.us/primaquine/primaquine.pdf.
[3] Kim et al Arch Pharm Res 2010. https://www.ncbi.nlm.nih.gov/pubmed/20512476
[4] Borsini et al AAC 2012: https://doi.org/10.1128/AAC.05688-11
[5] CHassan and Zunkler Parmacology 2005: https://doi.org/10.1159/000083234
[6] Yilma et al. American Journal of Tropical Medicine and Hygiene, 109(4):761-769, 4 Oct, 2023. https://doi.org/10.4269/ajtmh.23-0280
[7] Abeyaratne et al. Ceylon Medical Journal. 1968
[8] Lacerda et al, Clinical Infectious Diseases 2012. https://doi.org/10.1093/cid/cis615
[9] Brito-Sousa et al, Clinical Infectious Diseases 2019. https://doi.org/10.1093/cid/ciz122
[10] Moore et al et al. Int J Infect Dis 2023; https://doi.org/10.1016/j.ijid.2023.05.063
[11] Hanboonkunupakarn et al. Antimicrob Agents Chemother 2014; https://doi.org/10.1128/AAC.03704-14

SCOPE SERIOUS ADVERSE EVENT FORM

Staff Identification Number: FC/JA Study Site: Timika SCOPE ID: 1102

Summary

The SRT concluded that the most likely differential diagnosis of the cause of death was lethal cardiac arrhythmia or cerebral vascular haemorrhage.

Main key risk factors for lethal arrhythmia are domperidone (exacerbated by *vomiting and hypokalaemia*), piperaquine and female sex. Primaquine might have had a small contributory effect but, if this event is iatrogenic, the domperidone and piperaquine contributors are likely to be much more relevant.

Action

Based on the nature of this event and the literature review described above, the SRT proposes that the SCOPE study should recommence but recommends that henceforth, that in Stage 1 coadministration of domperidone for antiemesis, with DHA-piperaquine should avoided.

In view of the sensitivity of the SRT also requested that the SMC be informed of the case and decision to proceed with the study.

Appendix

Review of the literature on cardiotoxicity associated with use of primaquine

7 Studies identified (search terms "primaquine", "ECG", "arrhythmia", sudden death" - no date restriction) with information on ECGs following primaquine administration.

Study 1. Short-course, high-dose primaquine regimens for the treatment of liver stage vivax malaria in children

Moore et al et al. Short-course, high-dose primaquine regimens for the treatment of liver-stage vivax malaria in children. Int J Infect Dis 2023; ***134****: 114-22.*

- Participants were between the age of 5 and 10 years with vivax monoinfection in PNG
- The primaquine dosing groups were **0.5mg/kg/day** (n=15) (A), **1mg/kg/day** (n=40) (B) and **2mg/kg/day** (divided into 1mg/kg BD) (n=16) (C)
- Primaquine was given AFTER the last dose of artemether-lumefantrine.
- The formula used for calculation of QTc is unknown (possibly Bazett's).
- QTc was measured at baseline in all groups, before the 4th dose in groups B and C and before the 5th dose, 7th dose and final dose in group C. In group C (BD dosing), ECGs will have been taken approximately 12 hours after the previous dose. In group B (OD dosing), ECGs will have been taken approximately 24 hours after the previous dose.
- The median QTc was lower at all follow-up time points than at baseline in group B and group C
- The 75th centile (ie upper bound of the interquartile range) QTc was lower than the baseline median QTc at all follow-up timepoints in group B and group C
- Overall range of QTc at baseline and follow-up timepoints was not given so outliers are not represented.

Summary interpretation: In this small but well-designed study with intensive safety follow-up, there was no evidence to suggest that primaquine monotherapy prolongs QTc in 5-10 year-old children with vivax malaria, even at a dose of 2mg/kg/day.

Study 2. Pharmacokinetic Interactions between Primaquine and Chloroquine

Pukrittayakamee et al. Pharmacokinetic interactions between primaquine and chloroquine. Antimicrob Agents Chemother 2014; ***58****(6): 3354-9.*

- 16 healthy volunteer patients between the ages of 20 and 47 years. Primaquine **30mg as a STAT** dose and then repeated in combination with chloroquine at a later date
- Used Fridericia's correction for QTc
- ECGs done at 0, 1, 2, 4, 8, 12 and 24 hours after administration
- The mean of the maximum QTc change from baseline within 24 hours after primaquine alone was 1.20ms (SD +/- 2.33ms)
- The mean of the maximum QTc change from baseline within 24 hours after chloroquine alone was 6.1ms (SD +/- 3.67ms)

- The mean of the maximum QTc change from baseline within 24 hours after chloroquine + primaquine was 6.14ms (SD +/- 2.48ms)

Summary interpretation: No evidence of QTc prolongation in healthy adult volunteers after a single 30mg dose of primaquine. Addition of primaquine to chloroquine did not prolong the QTc any more than chloroquine monotherapy.

Study 3. A randomized, parallel study of the safety and efficacy of 45 mg primaquine versus 75 mg bulaquine as gametocytocidal agents in adults with blood schizonticide-responsive uncomplicated falciparum malaria

Gogtay NJ, Kamtekar KD, Dalvi SS, et al. A randomized, parallel study of the safety and efficacy of 45 mg primaquine versus 75 mg bulaquine as gametocytocidal agents in adults with blood schizonticide-responsive uncomplicated falciparum malaria [ISCRTN50134587]. BMC Infect Dis 2006; ***6****: 16.*

- 90 hospitalised adults with falciparum malaria treated with **45mg single dose** of primaquine on day 4 (n=31)
- Received quinine 10mg/kg TDS plus doxycycline 100mg BD for 7 days
- Stated that all electrocardiograms were within normal limits. No additional information given

Study 4. Open-label crossover study of primaquine and dihydroartemisinin-piperaquine pharmacokinetics in healthy adult Thai subjects

Hanboonkunupakarn et al. Open-label crossover study of primaquine and dihydroartemisinin-piperaquine pharmacokinetics in healthy adult thai subjects. Antimicrob Agents Chemother 2014; ***58****(12): 7340-6.*

- Randomised cross-over design study
- 16 healthy Thai adult volunteers received a single **30mg** primaquine dose, 3 tablets of DP (120/960mg) or both drugs together during three separate hospitalisations
- ECGs taken at hours 0, 1, 2, 4, 8, 12 and 24 hours after dose
- Fridericia's formula for QT correction
- Mean QTc was lower than baseline at all timepoints post primaquine dose
- Mean QTc changes after combination DP and primaquine treatment were as follows (-3.55ms at 1 hour, 0.06ms at 2 hours, 8.28ms at 4 hours, 3.79ms at 8 hours, -0.31ms at 12 hours and -0.95ms at 24 hours)
- There was no statistically significant difference in QTc change at any time point between DP alone and DP + primaquine

Summary interpretation: In healthy Thai adult volunteers, there was no evidence of QTc prolongation after a single dose of 30mg of primaquine and primaquine when added to DP did not appear to prolong the QTc any more than DP alone.

Study 5. Pharmacokinetic Interactions between Primaquine and Pyronaridine-Artesunate in Healthy Adult Thai Subjects

Jittamala et al. Pharmacokinetic interactions between primaquine and pyronaridine-artesunate in healthy adult Thai subjects. Antimicrob Agents Chemother 2015; ***59****(1): 505-13.*

- Randomised cross-over design
- 7 healthy adults aged between 18 and 60 years in Thailand received **30mg** primaquine alone and then were randomised to receive pyronaridine-artesunate alone or pyronaridine-artesunate plus primaquine in combination with intervening washout periods
- QT correction using Bazett's formula
- ECGs performed 2, 4, 8, 12 and 24 hours after dosing
- No clinically relevant effects on ECGs were observed. No additional information given.

Study 6. Pharmacokinetics of mefloquine in the presence of primaquine

Karbwang et al. Pharmacokinetic interactions between primaquine and pyronaridine-artesunate in healthy adult Thai subjects. Antimicrob Agents Chemother 2015; ***59****(1): 505-13.primaquine. Eur J Clin Pharmacol 1992;* ***42****(5): 559-60*

- 8 healthy Thai men aged 25-52 years
- Randomised cross-over design
- Received either primaquine 45mg plus mefloquine 750mg or mefloquine 750mg alone with subsequent cross-over after washout period
- ECGs done at 0, 1, 2, 4, 6, 8 and 24 hours
- Sinus arrhythmia found in the ECG of one volunteer on day 21 after mefloquine alone.
- No other reports on ECGs

Study 7. The arrhythmogenic cardiotoxicity of the quinoline and structurally related antimalarial drugs: a systematic review

Haeusler et al. The arrhythmogenic cardiotoxicity of the quinoline and structurally related antimalarial drugs: a systematic review. BMC Med 2018; ***16****(1): 200.*

- Systematic review of cardiotoxicity of quinoline antimalarials
- Philippe Guerin and Nick White were the senior authors
- 7 studies with safety data for 150 patients treated with primaquine found (variable dose), all 150 of whom had ECGs
- 71% of patients from Thailand
- Age range of patients 16-74 years
- No deaths in the 35,448 patients treated with a quinoline antimalarial in the included studies
- No arrhythmias detected in patients treated with primaquine
