# Supplementary material for: High-dose, short-course primaquine after point-of-care G6PD testing for the radical cure of Plasmodium vivax malaria: a safety study in Papua New Guinea and Indonesia
Source: Lancet Reg Health West Pac. 2026 Jun 11;71:101903. doi: 10.1016/j.lanwpc.2026.101903 (PMC13276568; doi:10.1016/j.lanwpc.2026.101903)
Supplement: Supplementary Fig. S1 and Tables S1–S7 [file mmc6.pdf]

High-dose, short-course primaquine after point-of-care G6PD testing for the radical cure of *Plasmodium vivax* malaria: a safety study in Papua New Guinea and Indonesia

SCOPE Study Group

Table of Contents

Table S1. Site Details 2

Table S2. Study procedures and data collection 3

Table S3. Protocol deviations for patients by site 4

Table S4. Adverse events in Stage 1, by treatment arm and country 5

Figure S1. Change in haemoglobin (g/dL) from baseline at Day 3 review (A), Day 7 review (A) and the maximum change in Hb during the first 10 days (C) 7

Table S5. Listing of haemolytic Adverse Events of Special Interest (AESI) 8

Table S6. Listing of gastrointestinal Adverse Events of Special Interest (AESI) 9

Table S7. Listing of All Serious Adverse Events (SAEs) 10

SCOPE Stage 1 – Supplementary Data

Table S1. Site Details

	Indonesia		Papua New Guinea	
Clinic name	**Timika**	**Wania**	**Mugil**	**Napapar**
Clinic coordinates	4°32'31"S 136°53'34"E	4°35'44"S 136°52'08"E	4°50'10"S 145°46'42"E	4°20'14"S 152°07'06"E
Altitude above sea level	24.1 meters	14 meters	41.8 meters	298.9 meters
Schizontocidal Treatment Policy	DHA-Piperaquine	DHA-Piperaquine	Artemether-Lumefantrine	Artemether-Lumefantrine
Standard Hypnozontocidal Treatment Policy	Primaquine (0.25mg/kg for 14 days)	Primaquine (0.25mg/kg for 14 days)	Primaquine (0.25mg/kg for 14 days)	Primaquine (0.25mg/kg for 14 days)
G6PD prevalence[a]				
Normal Activity	93.3% (180/193)	82.1% (160/195)	89.3% (175/196)	95.0% (190/200)
Intermediate Activity	6.2% (12/193)	14.9% (29/195)	6.12% (12/196)	3.0% (6/200)
Deficient	0.5% (1/193)	3.1% (6/195)	4.59% (9/196)	2.0% (4/200)
Seasonality of malaria	Perennial	Perennial	Perennial	Perennial
Total malaria case in previous year	6873	7388	1008	558
P. vivax (Pv)	3220	3257	-[b]	-[b]
P. falciparum (Pf)	3313	4063	-[b]	-[b]
Pf:Pv ratio	1.03	1.25	-[b]	-[b]
Relapse periodicity				
Vectors	*A. farauti, A. punctulatus, A. koliensis*	*A. farauti, A. punctulatus, A. koliensis*	*A. farauti, A. punctulatus, A. koliensis*	*A. farauti, A. punctulatus, A. koliensis*

[a]Data derived from pre-implementation survey at the clinic of 200 febrile patients. Normal defined as >70% G6PD activity, intermediate defined as 30-70% activity, and deficient defined as <30% activity.
[b] Diagnosis made with Rapid Diagnostic Tests and species breakdown not available

Table S2. Study procedures and data collection

	Day 0	Day 3	Day 7	Unplanned Visit
**Location**	Clinic	Community or clinic by a nurse	Community or clinic by a nurse	Clinic
**Routine review and diagnosis**	X			
**Consent**	X			
**Capillary blood sample**				
Hb	X	X	X	+/- X
G6PD	X			
Microscopy				+/- X
**Treatment/Prescription**	X			
**Education**	X			
**Supervision of first dose**	X			
**Drug supply**	X			
**Adverse event data collection**	X	X	X	X
**Community pharmacovigilance[1]**	X	X	X	X

[1] Reporting of any AESI/SAE identified during study participants' primaquine treatment

SCOPE Stage 1 – Supplementary Data

Table S3. Protocol deviations for patients by site

	Overall	Timika	Wania	Napapar	Mugil
**Total Enrolled**	N=800	N=258	N=242	N=150	N=150
**PRESCRIBING DEVIATIONS Incorrectly treated with primaquinw**					
**Pregnant women - N** (% of all female pts)	0 (0.0%)	0 (0.0%)	0 (0.0%)	0 (0.0%)	0 (0.0%)
**Breast Feeding infants** - N (% of all female pts)	0 (0.0%)	0 (0.0%)	0 (0.0%)	0 (0.0%)	0 (0.0%)
**Infants** - N (% of all enrolled pts)	0 (0.0%)	0 (0.0%)	0 (0.0%)	0 (0.0%)	0 (0.0%)
**Patients with Pf (Mono) -** N (% of all enrolled pts)	0 (0.0%)	0 (0.0%)	0 (0.0%)	0 (0.0%)	0 (0.0%)
**G6PD normal >70% N (% of all enrolled pts)**					
Treated with PQ14	0 (0.0%)	0 (0.0%)	0 (0.0%)	0 (0.0%)	0 (0.0%)
Treated with PQ8W	0 (0.0%)	0 (0.0%)	0 (0.0%)	0 (0.0%)	0 (0.0%)
**G6PD Intermediate 30-70% N (% of all enrolled pts)**					
Treated with PQ7	0 (0.0%)	0 (0.0%)	0 (0.0%)	0 (0.0%)	0 (0.0%)
Treated with PQ8W	0 (0.0%)	0 (0.0%)	0 (0.0%)	0 (0.0%)	0 (0.0%)
**G6PD Deficient <30% N (% of all enrolled pts)**					
Treated with PQ7	0 (0.0%)	0 (0.0%)	0 (0.0%)	0 (0.0%)	0 (0.0%)
Treated with PQ14	0 (0.0%)	0 (0.0%)	0 (0.0%)	0 (0.0%)	0 (0.0%)
**Hb (Biosensor) < 8 g/dL N (% of all enrolled pts)**	3 (0.4%)	0 (0.0%)	1 (0.4%)	0 (0.0%)	2 (1.3%)
**DOSING DEVIATIONS**					
**PQ7 N (%)**					
daily dose > 1.25mg/kg	0 (0.0%)	0 (0.0%)	0 (0.0%)	0 (0.0%)	0 (0.0%)
daily dose < 0.5mg/kg	0 (0.0%)	0 (0.0%)	0 (0.0%)	0 (0.0%)	0 (0.0%)
**PQ14 N (%)**					
daily dose > 0.83mg/kg	0 (0.0%)	0 (0.0%)	0 (0.0%)	0 (0.0%)	0 (0.0%)
daily dose < 0.25mg/kg	0 (0.0%)	0 (0.0%)	0 (0.0%)	0 (0.0%)	0 (0.0%)
**PQ8W N (%)**					
daily dose > 1.25mg/kg	0 (0.0%)	0 (0.0%)	0 (0.0%)	0 (0.0%)	0 (0.0%)
daily dose < 0.38mg/kg	0 (0.0%)	0 (0.0%)	0 (0.0%)	0 (0.0%)	0 (0.0%)
**FOLLOW UP DEVIATIONS (including withdrawn patients)** N (**% of all enrolled pts**)					
Patients without Day-3 Review (D3-5)	27 (3.4%)	7 (2.7%)	8 (3.3%)	9 (6.0%)	3 (2.0%)
Patients without Day-7 Review (D6-10)	35 (4.4%)	11 (4.3%)	10 (4.1%)	8 (5.3%)	6 (4.0%)
Patients without a practitioner-led review (where flagged by study nurse)	5[b] (0.6%)	1 (0.4%)	2 (0.8%)	1 (0.7%)	1 (0.7%)

[a] Criteria: <6 months in Indonesia and <12 months in PNG)

[b] Four of the patients flagged by the study nurse who did not have practioner-led review, met the criteria for AESIs

SCOPE Stage 1 – Supplementary Data

Table S4. Adverse events in Stage 1, by treatment arm and country

		Indonesia			PNG		
	**Overall**	**PQ7**	**PQ14**	**PQ8W**	**PQ7**	**PQ14**	**PQ8W**
**Total Enrolled**	N=800	N=347	N=134	N=19	N=279	N=14	N=7
**Patients with Day 3 (D3) Review**[b]	773 (96.6%)	337 (97.1%)	130 (97.0%)	18 (94.7%)	269 (96.4%)	14 (100.0%)	5 (71.4%)
Flagged Day 3-5 N (%)[a]	22 (2.8%)	5 (1.5%)	4 (3.1%)	1 (5.6%)	11 (4.0%)	1 (7.1%)	0 (0.0%)
**Patients with Day 7 (D7) Review**[b]	765 (95.6%)	333 (96.0%)	129 (96.3%)	17 (89.5%)	266 (95.3%)	14 (100.0%)	6 (85.7%)
Flagged Day 6-10 N (%)[a]	12 (1.5%)	1 (0.3%)	2 (1.5%)	1 (5.6%)	6 (2.2%)	1 (7.1%)	1 (16.7%)
Other Day Flagged N (%)[a]	1 (0.1%)	0 (0.0%)	0 (0.0%)	0 (0.0%)	0 (0.0%)	0 (0.0%)	1 (16.7%)
**Adverse Event of Special Interest (AESI)** N (%)[a]							
**Haemolysis N (%)***							
AESI (SAE)	4 (0.5%)	1 (0.3%)	0 (0.0%)	0 (0.0%)	3 (1.1%)	0 (0.0%)	0 (0.0%)
AESI (non-SAE)	20 (2.6%)	3 (0.9%)	5 (3.8%)	2 (11.1%)	8 (2.9%)	1 (7.1%)	1 (16.7%)
**Total**	24 (3.1%)	4 (1.2%)	5 (3.8%)	2 (11.1%)	11 (4.0%)	1 (7.1%)	1 (16.7%)
**Gastrointestinal N (%)***							
AESI (SAE)	3 (0.4%)	1 (0.3%)	0 (0.0%)	0 (0.0%)	2 (0.7%)	0 (0.0%)	0 (0.0%)
AESI (non-SAE)	0 (0.0%)	0 (0.0%)	0 (0.0%)	0 (0.0%)	0 (0.0%)	0 (0.0%)	0 (0.0%)
**Total**	3 (0.4%)	1 (0.3%)	0 (0.0%)	0 (0.0%)	2 (0.7%)	0 (0.0%)	0 (0.0%)
**Methaemoglobinaemia N (%)***							
AESI (SAE)	0 (0.0%)	0 (0.0%)	0 (0.0%)	0 (0.0%)	0 (0.0%)	0 (0.0%)	0 (0.0%)
AESI (non-SAE)	0 (0.0%)	0 (0.0%)	0 (0.0%)	0 (0.0%)	0 (0.0%)	0 (0.0%)	0 (0.0%)
**Total**	0 (0.0%)	0 (0.0%)	0 (0.0%)	0 (0.0%)	0 (0.0%)	0 (0.0%)	0 (0.0%)

**Severe Adverse Events (SAEs)** N (%) [b]							
SAE – Related (possibly, probably, definitely)	9 (1.1%)	2 (0.6%)	0 (0.0%)	0 (0.0%)	7 (2.5%)	0 (0.0%)	0 (0.0%)
SAU – Unrelated (not related, unlikely)	7 (0.9%)	2 (0.6%)	0 (0.0%)	0 (0.0%)	5 (1.8%)	0 (0.0%)	0 (0.0%)
**Total**	16 (2.0%)	4 (1.2%)	0 (0.0%)	0 (0.0%)	12 (4.3%)	0 (0.0%)	0 (0.0%)
Related SAE, which is also an AESI	2 (0.3%)	0 (0.0%)	0 (0.0%)	0 (0.0%)	2 (0.7%)	0 (0.0%)	0 (0.0%)
Related SAE Other	7 (0.9%)	2 (0.6%)	0 (0.0%)	0 (0.0%)	5 (1.8%)	0 (0.0%)	0 (0.0%)

PQ7: Primaquine 1.0 mg/kg/day for 7 days; PQ14: Primaquine 0.5 mg/kg/day for 14 days; PQ8W: Primaquine 0.75 mg/kg once a week for 8 weeks

[a] Denominator = number of patients reviewed by the study team at any day (**Overall** N=783. **Indonesia**: PQ7 N=338, PQ14 N=130, PQ8W N=18. **PNG**: PQ7 N=277, PQ14 N=14, PQ8W N=6.

[b] Denominator = number of patients enrolled and treated with the PQ regimen (**Overall** N=800. **Indonesia**: PQ7 N=347, PQ14 N=134, PQ8W N=19. **PNG**: PQ7 N=279, PQ14 N=14, PQ8W N=7.

AESIs are defined as per protocol.

SCOPE Stage 1 – Supplementary Data

Figure S1. Change in haemoglobin (g/dL) from baseline at Day 3 review (A), Day 7 review (A) and the maximum change in Hb during the first 10 days (C)

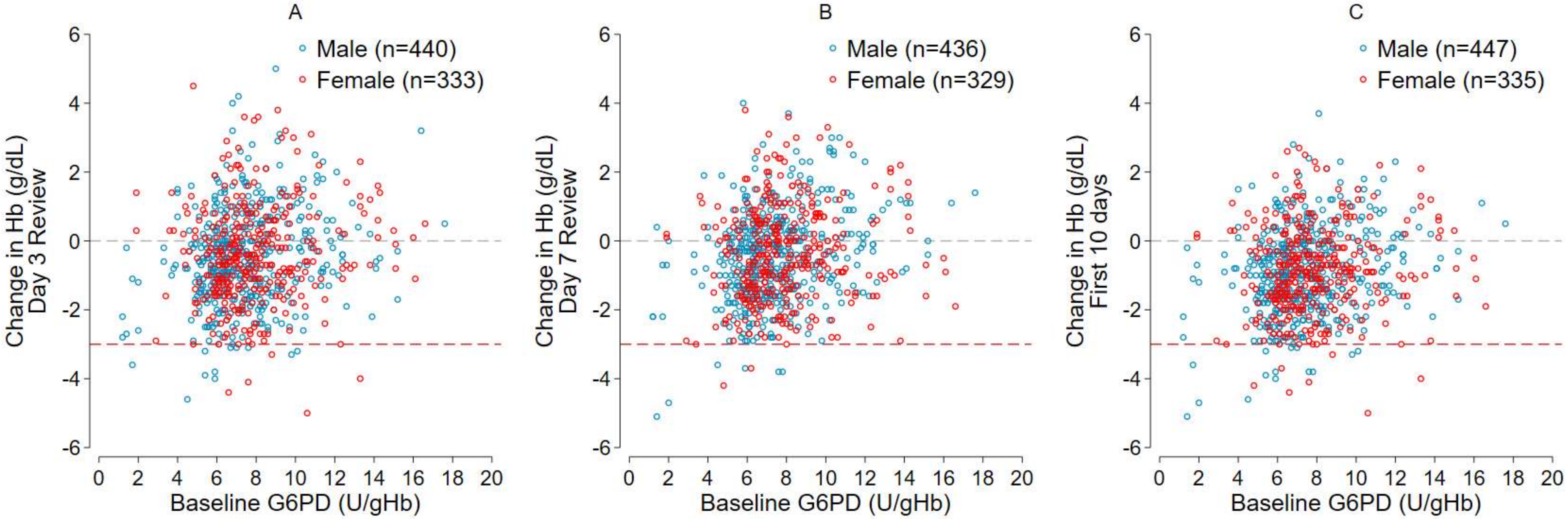

Footnote: Haemoglobin readings for the day 3 timepoint were taken as the minimum recorded haemoglobin between day 3 and day 5, and for the day 7 timepoint were the minimum recorded haemoglobin per patient between days 6 and 10. Positive change indicates an increase in haemoglobin. Horizontal red dotted lines reflect the threshold use to define haemolysis – a reduction in Hb >3 g/dL.

SCOPE Stage 1 – Supplementary Data

Table S5. Listing of haemolytic Adverse Events of Special Interest (AESI)

	Country	Age	Sex	PQ	Hb 0	Hb 3	Hb 4	Hb 5	Hb 6	Hb 7	Hb 8	Hb 9	Hb 10	Hb nadir	Hb max drop	AESI	Start Day	PQ Status
1005	Indonesia	23	Male	PQ7	15.0	11.9	14.3			13.1				11.9	3.1	Hb fall >3.0 g/dL	3	No modification
1007	Indonesia	15	Female	PQ14	14.5	11.5				10.3				10.3	4.2	Hb fall >3.0 g/dL	7	No modification
1240	Indonesia	30	Male	PQ8W	14.7	11.4	11.1	11		12.5				11.1	3.6	Hb fall >3.0 g/dL	3	No modification
2001	Indonesia	52	Male	PQ14	16.7	12.1	13.2			13.1				12.1	4.6	Hb fall >3.0 g/dL	3	No modification
2002	Indonesia	39	Male	PQ14	15.5	12.4	12.3			15.2				12.3	3.2	Hb fall >3.0 g/dL	3	No modification
2016	Indonesia	12	Female	PQ7	12.0	8.7				12.9				8.7	3.3	Hb fall >3.0 g/dL	3	No modification
2019	Indonesia	35	Male	PQ14	16.8	12.9					14.7			12.9	3.9	Hb fall >3.0 g/dL	3	No modification
2022	Indonesia	10	Female	PQ7	10.7	6.6	13.8	7.1		10.6				6.6	4.1	Hb fall >3.0 g/dL, Min Hb <7.0 g/dL	3	No modification
2038	Indonesia	44	Male	PQ7	14.6	15.1							13.9	13.9	0.7	Grade 3/4 symptoms	3	No modification
2043	Indonesia	24	Male	PQ14	15.2	11.2		12.8		13.0				11.2	4.0	Hb fall >3.0 g/dL	3	No modification
2221	Indonesia	30	Male	PQ8W	15.8	13.1				10.7	12.4			10.7	5.1	Hb fall >3.0 g/dL	7	No modification
8002	PNG	15	Female	PQ7	11.1	6.7		11.5		11.4				6.7	4.4	Hb fall >3.0 g/dL, Min Hb <7.0 g/dL	3	No modification
8056	PNG	1	Female	PQ7	9.3	8.6					6.4			6.4	2.9	Min Hb <7.0 g/dL	8	No modification
8061	PNG	4	Female	PQ7	11.1	7.1	7.9	8.1					10.2	7.1	4.0	Hb fall >3.0 g/dL	3	No modification
8077	PNG	21	Female	PQ7	9.2	8.1				5.5				5.5	3.7	Hb fall >3.0 g/dL, Min Hb <7.0 g/dL	7	No modification
8078	PNG	11	Male	PQ7	11.6	8.5				7.8			9.2	7.8	3.8	Hb fall >3.0 g/dL	3	No modification
8082	PNG	6	Male	PQ7	11.6	8.3	8.7			8.7				8.3	3.3	Hb fall >3.0 g/dL	3	No modification
8105	PNG	11	Male	PQ8W	11.8	9.2				7.1	8.5			7.1	4.7	Hb fall >3.0 g/dL	7	No modification
8107[ab]	PNG	7	Male	PQ7	9.8	7.4	6.6	7.2				8.8		6.6	3.2	Hb fall >3.0 g/dL, Min Hb <7.0 g/dL	4	Ceased
9001	PNG	9	Male	PQ14	12.2	9.6		8.4	9.4	9.4		8.5		8.4	3.8	Hb fall >3.0 g/dL	5	Ceased
9034	PNG	4	Female	PQ7	13.2	10.0	8.2				10.4			8.2	5.0	Severe pallor/jaundice, Hb fall >3.0 g/dL	3	No modification
9078	PNG	18	Male	PQ7	14.8	11.7	12.9			14.7				11.7	3.1	Hb fall >3.0 g/dL	3	No modification
9121[a]	PNG	12	Male	PQ7	11.8	10.2				8.0	10.5		10.2	8.0	3.8	Hb fall >3.0 g/dL	7	No modification
9137[a]	PNG	5	Female	PQ7	10.7	12.1				12.2		11.0		10.7	n/a	Grade 3/4 symptoms	9	Ceased

[a] Haemolysis AESI also classified as SAE; [b] Withdrew from the study

SCOPE Stage 1 – Supplementary Data

Table S6. Listing of gastrointestinal Adverse Events of Special Interest (AESI)

ID	Country	Site	Age	Sex	PQ	AESI	Start Day	PQ Status
2093 [ab]	Indonesia	Wania	24	Male	PQ7	Nausea and vomiting	0	Modified
9004 [a]	PNG	Napapar	13	Male	PQ7	Abdominal pain grade 4	1	Ceased
9137 [a]	PNG	Napapar	5	Female	PQ7	Nausea, vomiting and fatigue grade 3	8	Ceased

[a] Gastrointestinal AESI also classified as SAE
[b] Withdrew from study

SCOPE Stage 1 – Supplementary Data

Table S7. Listing of All Serious Adverse Events (SAEs)

ID	Country	Age	Sex	PQ	Daily dose	SAE Diagnosis	Relatedness	Day Adverse Event Started	PQ Status
2038	Indonesia	44	Male	PQ7	0.72	Dengue fever with pneumonia	Not related	0	No modification
9004	Papua New Guinea	13	Male	PQ7	1.05	Appendicitis	Not related	1	Ceased
9070	Papua New Guinea	11	Female	PQ7	1	Cellulitis	Not related	1	No modification
1102	Indonesia	13	Female	PQ7	0.99	Death on arrival	Unlikely related	0	N/A
8098	Papua New Guinea	15	Male	PQ7	0.96	Acute gastroenteritis	Unlikely related	4	N/A
8094	Papua New Guinea	23	Female	PQ7	1.18	Acute gastroenteritis	Unlikely related	5	N/A
8107	Papua New Guinea	7	Male	PQ7	0.89	Acute gastroenteritis	Unlikely related	9	Ceased
9089	Papua New Guinea	24	Female	PQ7	1.15	Acute gastroenteritis/food poisoning	Possibly related	1	No modification
8124	Papua New Guinea	18	Female	PQ7	1.13	Acute gastroenteritis	Possibly related	3	Ceased
8084	Papua New Guinea	15	Male	PQ7	1.11	Acute gastroenteritis	Possibly related	4	N/A
9121	Papua New Guinea	12	Male	PQ7	1.15	Acute gastroenteritis	Possibly related	5	No modification
8118	Papua New Guinea	12	Male	PQ7	0.79	Acute gastroenteritis	Possibly related	8	N/A
8119	Papua New Guinea	16	Female	PQ7	1.13	Acute gastroenteritis	Possibly related	8	N/A
9137	Papua New Guinea	5	Female	PQ7	0.94	Acute gastroenteritis	Possibly related	8	Ceased
2093	Indonesia	24	Male	PQ7	0.83	Acute gastroenteritis<br>Hospitalised due to vomiting and nausea,<br>Uncomplicated malaria with hypokalemia	Probably related	0	Modified
1176	Indonesia	31	Female	PQ7	1.03	O2 desaturation caused by methaemoglobinemia	Probably related	7	No modification
